# Transannular Enantioselective (3 + 2) Cycloaddition of Cycloalkenone Hydrazones under Brønsted Acid Catalysis

**DOI:** 10.1021/acs.orglett.1c03190

**Published:** 2021-11-02

**Authors:** Jana Sendra, Efraim Reyes, Liher Prieto, Elena Fernández, Jose L. Vicario

**Affiliations:** † Department of Organic and Inorganic Chemistry, University of the Basque Country (UPV/EHU). P.O. Box 644, 48080 Bilbao, Spain; ‡ Departament Química Física i Inorgànica, Universidad Rovira i Virgilli, C/Marcel·lí Domingo s/n, 50009 Tarragona, Spain

## Abstract

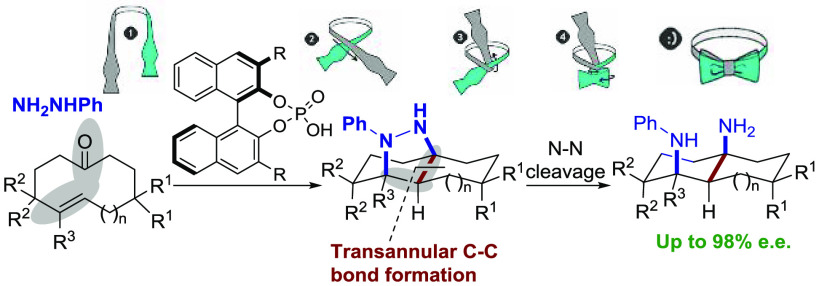

Hydrazones derived from cycloalkenones undergo an enantioselective transannular formal (3 + 2) cycloaddition catalyzed by a chiral phosphoric acid. The reaction provides high yields and excellent stereocontrol in the formation of complex adducts with one or two α-tertiary amine moieties at the ring fusion, and these can be converted into very versatile stereodefined decalin- or octahydro-1*H*-indene-derived 1,3-diamines through simple reductive N–N cleavage.

Transannular reactions, in which two reacting sites are connected to each other as part of a medium- or large-size cyclic starting material, represent an unconventional strategic decision in organic synthesis that enables the rapid construction of complex polycyclic molecular scaffolds.^1^ In fact, there are many reports of elegant total syntheses that make use of transannular reactions to build up the key structural framework of the final target,^2^ including several examples of biomimetic approaches that show that this type of reactivity is also operating as part of the portfolio of chemical reactions in the secondary metabolism of living cells. Despite all of the advances in the area, the majority of the reports still rely on the use of chiral cyclic substrates as starting materials, therefore involving the diastereoselective generation of new stereogenic centers during the transannular process.^2^ This implies that the stereochemical outcome of the reaction is strictly under substrate control, and consequently, it is largely conditioned by the innate asymmetric induction profile of the chiral starting material. In contrast, enantioselective versions of transannular reactions have received very little attention, and only a few limited reports can be found in the literature that comprise a couple of examples in which stoichiometric amounts of a chiral ligand or promoter are used in transannular aldol^3^ or Rauhult–Currier reactions.^4^ Catalytic and enantioselective transannular reactions are limited to three cases of transformations under Lewis acid catalysis, such as transannular Diels–Alder,^5^ ketone-ene,^6^ and Claisen rearrangement,^7^ and to one example of a transannular aldol reaction under enamine catalysis.^8^ On the contrary, and very recently, we also demonstrated the excellent performance of catalytic transannular reactions in the enantioselective synthesis of complex polycyclic systems with the development of a transannular Morita–Baylis–Hillman reaction under chiral phosphine catalysis,^9^ a Michael-initiated cascade reaction under bifunctional tertiary amine/squaramide catalysis,^10^ and a copper-catalyzed transannular borylative ring-closing process.^11^

We present herein the use of hydrazones derived from cycloalkenones as substrates that undergo enantioselective transannular (3 + 2) cycloaddition^12^ under catalysis by a BINOL-based chiral Brønsted acid (Scheme 1, bottom).

**Scheme 1:**
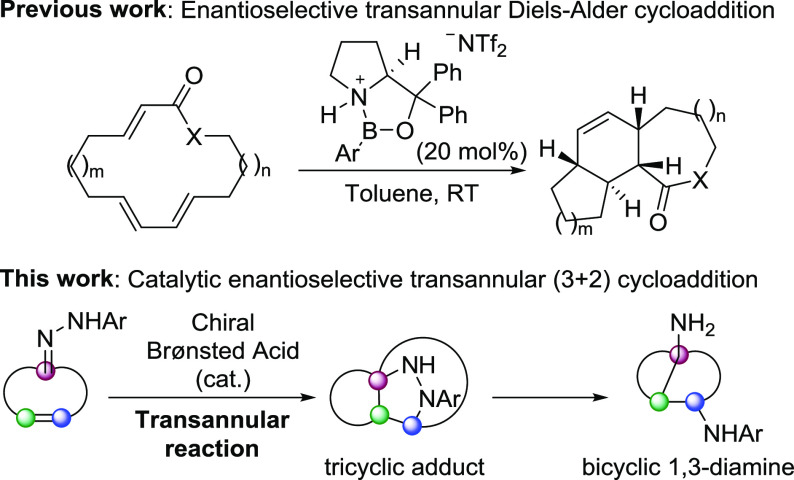
Enantioselective Transannular Diels-Alder Reaction and the Brønsted-Acid-Catalyzed Transannular (3 + 2) Cycloaddition of Cycloalkenone Hydrazones

In comparison with the only existing literature precedent of an enantioselective transannular cycloaddition (the transannular Diels–Alder cycloaddition developed by Jacobsen and coworkers shown in Scheme 1, top),^5^ this new reaction leads to tricyclic scaffolds with a bridging hydrazine moiety, therefore providing a direct alternative entry to compounds whose structures resemble the type of adducts that can be accessed through type-II intramolecular cycloadditions.^13^ Remarkably, the adducts obtained through this transannular (3 + 2) cycloaddition are direct precursors to orthogonal and stereodefined bicyclic 1,3-diamines, which are key structural motifs in many natural products and also serve as highly versatile chiral building blocks in synthetic organic chemistry.^14^ Finally, it should also be pointed out that the number of examples of catalytic and enantioselective (3 + 2) cycloadditions between hydrazones and alkenes is very scarce, in most cases involving electron-poor *N*-acyl hydrazones together with electronically biased alkenes as dipolarophiles, such as enol ethers and thioethers, styrenes, or cyclopentadiene.^15^

We first started our work by evaluating the viability of the reaction using ketone **1a** as a model substrate and phenylhydrazine, envisaging the *in situ* formation of the hydrazine intermediate that would subsequently undergo the transannular (3 + 2) cycloaddition (Table 1).

**Table 1:**
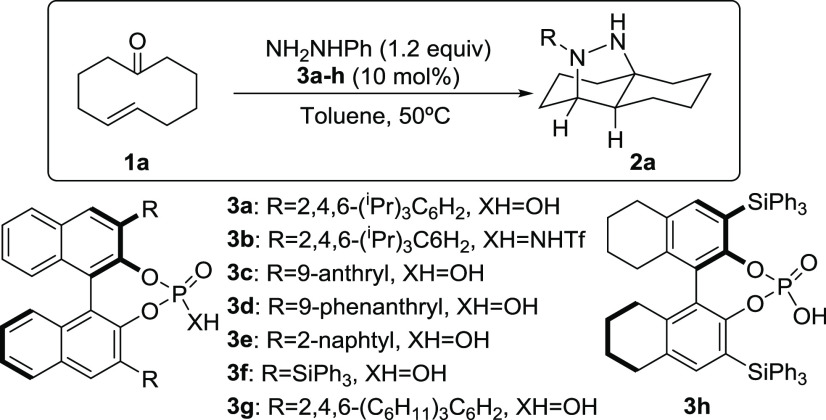
Optimization of the Reaction^a^

entry	catalyst	*T* (°C)	conv (%)^b^	e.e. (%)^c^
1	(PhO)_2_P(O)OH	r.t.	<5^d^	
2	(PhO)_2_P(O)OH	50	99 (72)	
3	**3a**	50	99	85
4	**3b**	50	55	25
5	**3c**	50	99	33
6	**3d**	50	99	23
7	**3e**	50	99	17
8	**3f**	50	99 (92)	88
9	**3g**	50	99 (99)	98
10	**3h**	50	99 (90)	90
11	**3g**	r.t.	<5^d^	n.d.
12^e^	**3g**	50	99 (99)	96

a Reactions were performed with 0.15 mmol of **1a**, NH_2_NHPh (1.2 equiv), catalyst (10 mol %), and toluene (0.1 M) as the solvent.

b Conversion was calculated by ^1^H NMR using 1,3,5-trimethoxybenzene as the internal standard. Isolated yield after flash column chromatography purification is given in parentheses.

c e.e. was calculated by HPLC in the chiral stationary phase after derivatization into the corresponding benzoyl hydrazide. (See the Supporting Information.) n.d., not determined.

d Starting material was recovered as the corresponding hydrazone.

e 5 mol % of catalyst was used.

As can be seen in this table, the reaction using diphenylphosphoric acid as the catalyst at room temperature (r.t.) was unsuccessful (entry 1), but heating the mixture to 50 °C resulted in the complete conversion of the starting material and a good isolated yield of the desired cycloaddition product (entry 2). We next moved to the archetypical chiral BINOL-based phosphoric acid TRIP,^16^ which also was demonstrated to be a good catalyst for the transformation of **1a** into **2a**, the latter being formed with 85% e.e. (entry 3). We also surveyed the corresponding *N*-Tf sulfonamide **3b** as a more acidic and potentially more active catalyst but with poorer results (entry 4). Next, phosphoric acid catalysts with different substituents at the 3- and 3′-positions of the BINOL core were surveyed (entries 5–10).^17^ We observed that placing extended aryl moieties led to a significant decrease in the enantioselectivity (entries 5–7), whereas moving to the SiPh_3_-containing catalyst **3f** resulted in the formation of adduct **2a** with a high 88% e.e. (entry 8). Improved enantioselectivity was obtained using either the bulkier analogue of TRIP (catalyst **3g**, entry 9) or its partially hydrogenated version (catalyst **3h**, entry 10). We observed the best result with the former. We also tested the reaction with this catalyst at lower temperature, verifying the need for 50 °C for quantitative cycloaddition (entry 11). Finally, we also observed that the reaction performed excellently using a 5 mol % catalyst loading (entry 12).

With a robust experimental protocol in hand, we next focused on studying the scope and limitations of the reaction, starting with the role played by the nature of the hydrazine substituent (Table 2).

**Table 2:**
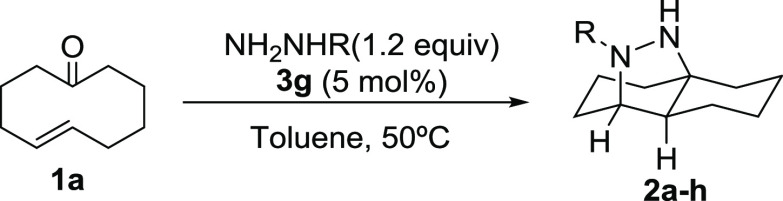
Scope of the Reaction: Hydrazine Component^a^

entry	R	yield (%)^b^	e.e. (%)^c^
1	C_6_H_5_ (**2a**)	99	96
2	C_6_F_5_ (**2b**)	96	90
3	4-CF_3_C_6_H_4_ (**2c**)	95	83
4	4-BrC_6_H_4_ (**2d**)	84	96
5	3,5-(CF_3_)_2_C_6_H_3_ (**2e**)	90	72
6	4-MeC_6_H_4_ (**2f**)	99	94
7	4-MeOC_6_H_4_ (**2g**)	<5^d^	n.d.
8	C(O)C_6_H_5_ (**2h**)	85	0
9^e^	C(O)C_6_H_5_ (**2h**)	40	0
10^e^	Ts	<5	n.d.
11	Bn	<5^d^	n.d.

a Reactions were performed with 0.15 mmol of **1a**, NH_2_NHR (1.2 equiv), **3g** (5 mol %), and toluene (0.1 M) at 50 °C.

b Isolated yield after flash column chromatography purification.

c e.e. was calculated by HPLC in the chiral stationary phase after derivatization into the corresponding benzoyl or acetyl hydrazide. (See the Supporting Information.)

d Starting material was recovered as the corresponding hydrazone.

e Reaction carried out at r.t.

Arylhydrazines with electron-withdrawing substituents performed excellently, providing the transannular cycloaddition products **2a**–**e** in excellent yields with excellent enantioselectivities (entries 1–5), with the only exception being the use of *meta*-bis-CF_3_-substituted hydrazine (entry 5), which provided adduct **2e** with somewhat lower e.e. When tolylhydrazine was used, the reaction also took place very efficiently (entry 6), but when the more electron-donating *para*-methoxyphenylhydrazine was tested, the reaction was completely suppressed, isolating the hydrazone formed upon condensation of the hydrazide with the starting material (entry 7). *N*-Benzoylhydrazine was also tested, and we observed a remarkably fast reaction and the quantitative conversion to the cycloaddition product **2h**, albeit as a completely racemic material (entry 8). We tested the reaction at a lower temperature to favor the enantioselective pathway but without any improvement and with a remarkable drop in the yield (entry 9). Alkyl hydrazones were also unreactive under these conditions. (See one example in entry 10.)

Several cycloalkenones were also surveyed in the transformation in combination with phenylhydrazine (Table 3). Initially, we tested the reaction on a higher 1.0 mmol scale of model substrate **1a** to guarantee its viability for preparative purposes. As can be seen in Table 3, adduct **2a** was obtained in good yield (83%) and with the same enantioselectivity (96% e.e.) as before. We also evaluated cycloalkenones **1a**–**f** with different sizes and substitution patterns. As can also be seen in Table 3, in all cases, the reaction provided the corresponding tricyclic adducts in excellent yields with excellent enantioselectivities. This transformation enables the preparation of adducts with an octahydro-2*H*-1,4*a*-epidiazanonaphthalene core, including the possibility of incorporating different substituents at the carbon scaffold (compounds **2a**, **2i**, **2j**, and **2k**). Moreover, the reaction leading to adducts with an octahydro-3*a*,7-epidiazanoindene core (compounds **2l** and **2m**) was also very efficient. Remarkably, this transformation also allows the generation of challenging structures such **2k** and **2m**, in which two α-tertiary hydrazine stereogenic centers are simultaneously generated in excellent yield with high stereocontrol.

**Table 3:**
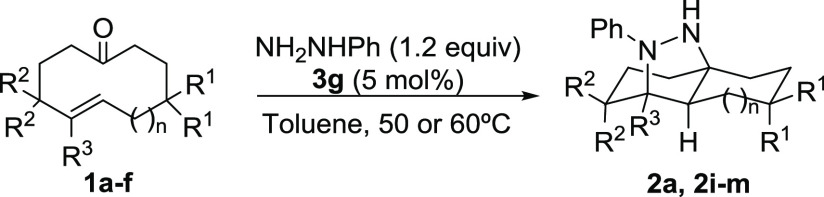

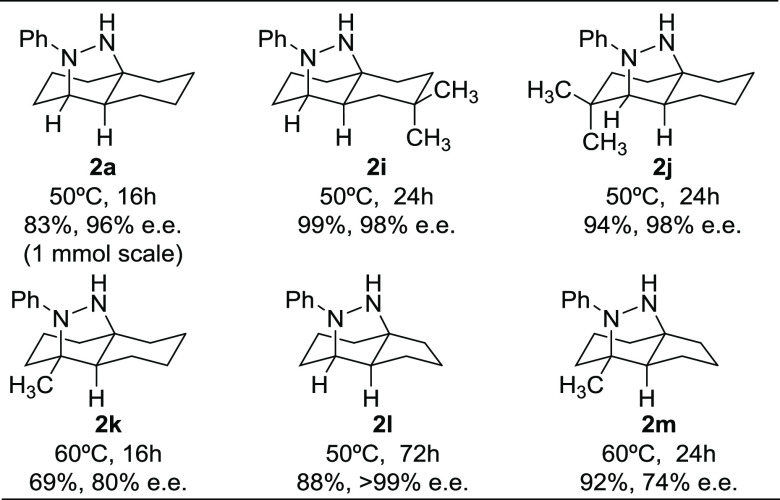
Scope of the Reaction: Cyclic Ketone Reagent^a^

a Reactions were performed with 0.15 mmol of **1a**–**f**, NH_2_NHPh (1.2 equiv), **3g** (5 mol %), and toluene (0.1 M) at the indicated temperature. Isolated yields after flash column chromatography purification are given. e.e. was calculated by HPLC in the chiral stationary phase after derivatization into the corresponding benzoyl or acetyl hydrazide. (See the Supporting Information.)

The absolute configuration of **2j** was determined by X-ray analysis of the corresponding *N*-benzoyl derivative (see the Supporting Information for details), and the configurations of all other adducts **2a**–**m** were established based on mechanistic analogy. This configuration is also in agreement with the reported stereochemical model for the intermolecular addition of activated alkenes to hydrazones under phosphoric acid catalysis.^15a^

We also evaluated the performance of chiral substrate **1g** to get further insight into the natural reactivity trend of this type of cycloalkenones toward the transannular cycloaddition reaction (Scheme 2). In fact, the reaction of **1g** under activation by the achiral catalyst diphenylphosphoric acid cleanly furnished adduct **2n** as a single diastereosiomer, although in a rather low yield, even after a prolonged reaction time. On the contrary, the reaction catalyzed by **3g** proceeded smoothly to form the same compound in a much higher yield, whereas the reaction performed using its enantiomer **(*****R*****)-3g** as a catalyst also provided the same diastereoisomer but, again, in a much lower yield. These experiments indicate a strong stereochemical bias exerted by the chiral information of the starting material, although with a very important matched/mismatched situation when incorporating a chiral Brønsted acid to promote the reaction.

**Scheme 2:**
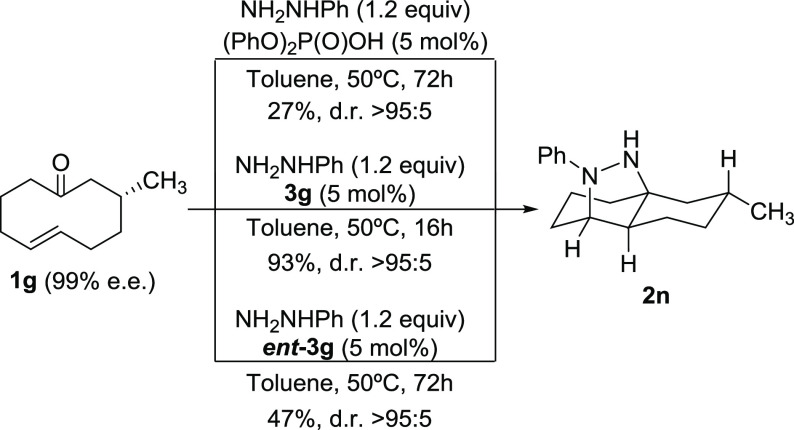
Use of Chiral Ketone **1g** as the Substrate

Finally, we decided to unmask the latent 1,3-diamine functionality present on adducts **2**, which are obtained through the enantioselective transannular cycloaddition process (Table 4). The particular arrangement of nitrogen atoms in these adducts would lead to the formation of compounds with a decaline or octahydro-1*H*-indene molecular architecture that would contain two amine substituents located in pseudoaxial positions, which is a molecular arrangement that is difficult to obtain through conventional approaches. This was accomplished by carrying out the hydrogenolytic cleavage of the N–N bond by reacting these adducts with hydrogen under Raney nickel catalysis. We initially optimized the reaction conditions using compound **2a** as a model substrate and obtained diamine **4a** in excellent yield when the reaction was carried out in ethanol at 80 °C (entry 1). We also verified that there was no loss of optical purity during the process by measuring the enantiomeric excess of the final product **4a** by high-performance liquid chromatography (HPLC) on a chiral stationary phase under conditions optimized for a racemic standard. With the optimized reductive cleavage conditions in hand, we generalized this reaction to the other adducts **2i**–**j**, obtaining in all cases the expected bicyclic 1,3-diamines **4b**–**g** in almost quantitative yields in most cases. As can be seen in Table 4, this approach enables the synthesis of octahydronaphthalene-1,4*a*(2*H*)-diamines (entries 1–4 and 7) or octahydro-3*aH*-indene-3*a*,7-diamines (entries 5 and 6) in excellent overall yields and as highly enantioenriched materials. This also includes the possibility of generating scaffolds containing two α-tertiary amine moieties that are challenging structures that cannot be accessed through conventional methodologies.^18^ In this case, these types of compounds were cleanly obtained from adducts **2k** and **2m** with high enantio- and diastereocontrol. (See entries 4 and 6.)

**Table 4:**
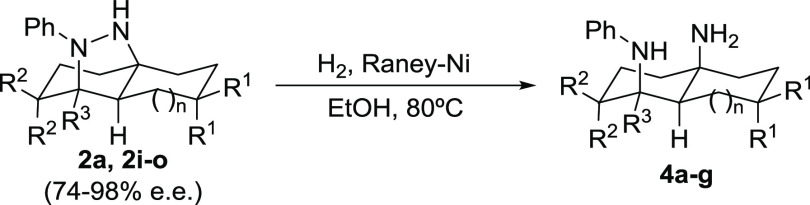

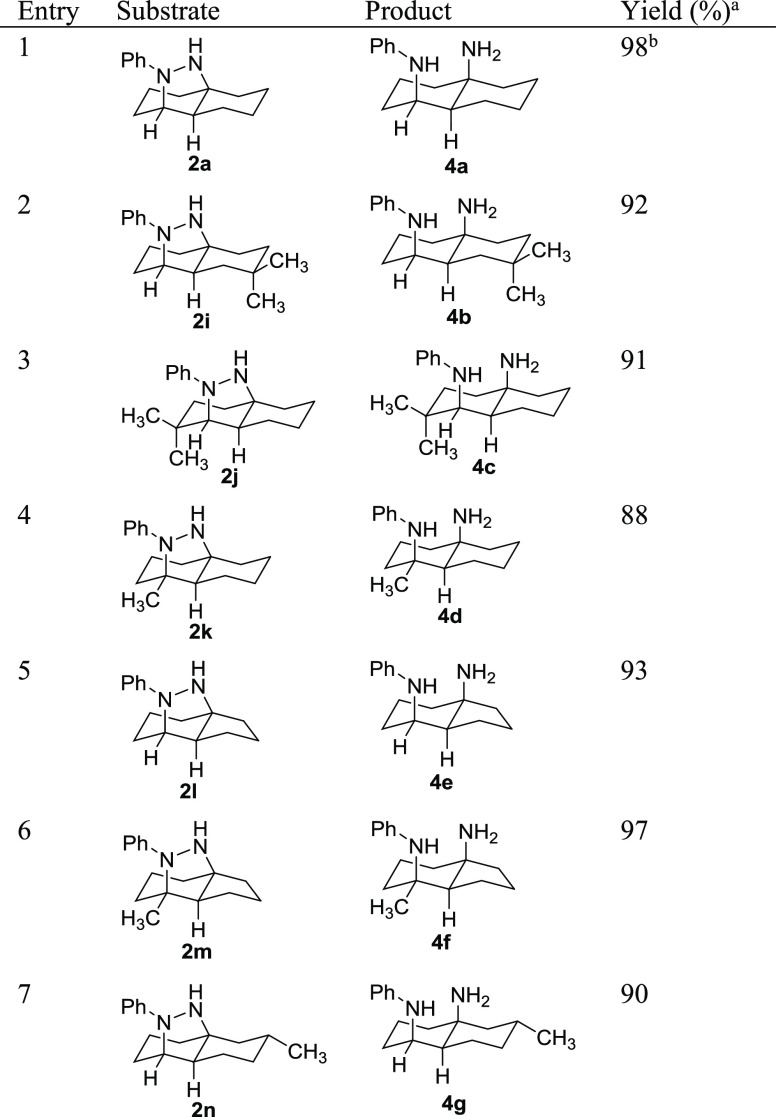
Reductive Cleavage of the Hydrazine Moiety: Synthesis of Enantioenriched 1,3-Diamines

a Isolated yield after flash column chromatography.

b e.e. 92%

In conclusion, we have demonstrated the ability of hydrazones derived from cycloalkenones to undergo enantioselective transannular formal (3 + 2) cycloaddition under catalysis by a chiral Brønsted acid derived from BINOL. This simple reaction provides stereodefined tricyclic adducts in high yields with high enantioselectivities, and these can be used as an ideal platform for the preparation of decaline- or octahydro-1*H*-indene- derived 1,3-diamines with great potential to be used as synthetic intermediates or chiral ligands and that are otherwise challenging compounds to access through conventional methodologies. This type of enantioselective transannular reactivity can be established as an alternative and less conventional disconnection when planning the total synthesis of complex molecules.

## Supporting Information Available

Experimental procedures, characterization of all new compounds, and copies of ^1^H and ^13^C NMR spectra. HPLC traces of all adducts prepared and crystallographic data (PDF)

## Accession Codes

CCDC 2091628 contains the supplementary crystallographic data for this paper. These data can be obtained free of charge via www.ccdc.cam.ac.uk/data_request/cif, or by emailing data_request@ccdc.cam.ac.uk, or by contacting The Cambridge Crystallographic Data Centre, 12 Union Road, Cambridge CB2 1EZ, UK; fax: +44 1223 336033.

## Dedication

This work is dedicated to the memory of our colleague and friend Prof. Kilian Muñiz.
