# Supplementary material for: Transannular Enantioselective (3 + 2) Cycloaddition of Cycloalkenone Hydrazones under Brønsted Acid Catalysis
Source: Org Lett. 2021 Nov 2;23(22):8738–43. doi: 10.1021/acs.orglett.1c03190 (PMC8609578; doi:10.1021/acs.orglett.1c03190)
Supplement: Supplementary file 1 — ol1c03190_si_001.pdf [file ol1c03190_si_001.pdf]

Supporting information

Transannular Enantioselective (3+2) Cycloaddition of Cycloalkenone Hydrazones under Brønsted Acid Catalysis.

Jana Sendra,[a,b] Efraim Reyes,[a] Liher Prieto,[a] Elena Fernández,*[b] Jose L. Vicario*[a]

[a] Department of Organic and Inorganic Chemistry
University of the Basque Country (UPV/EHU)
P.O Box 644, 48080 Bilbao (Spain)
E-mail: joseluis.vicario@ehu.eus

[b] Department of Physical and Inorganic Chemistry
University Rovira i Virgili
C/Marcel·lí Domingo s/n, 43007 Tarragona (Spain)
E-mail: mariaelena.fernandez@urv.cat

Contents

1.	General information	SI-3
2.	Experimental procedures and spectral data	SI-4
2.1.	Optimization of the reaction conditions	SI-4
2.2.	Synthesis of starting materials	SI-5
2.3.	*General Procedure I* for the enantioselective transannular (3+2) cycloaddition	SI-9
2.4.	*General Procedure II* for the benzoylation of **2a**, **2b**, **2d**, **2i**, **2j**, **2k**, **2l** and **2m**	SI-9
2.5.	*General Procedure III* for the acetylation of **2c**, **2e** and **2f**	SI-9
2.6.	Synthesis of enantioenriched 1,3-diamines*.*	
	General Procedure for the reductive cleavage of **4a**-**g**	SI-9
3.	NMR spectra	SI-22
4.	HPLC traces	SI-66
5.	X-ray details	SI-79

1. General information

Analytical grade solvents and *commercially available reagents* were used without further purification. *Anhydrous solvents* were purified and dried with activated molecular sieves prior to use.[1] For reactions carried out under inert conditions, the argon was previously dried through a column of P_2O_5 and $CaCl_2$. All the glassware was dried for 12 hours prior to use in an oven at 140ºC, and allowed to cool under a dehumidified atmosphere. Reactions were monitored using analytical thin layer chromatography (TLC), in pre-coated silica-backed plates (Merck Kiesegel 60 F_{254}). *Flash column chromatography* was performed on standard silica gel (Silicycle 40-63, 230-400 mesh) using standard visualizing agents: UV fluorescence (254 and 366 nm), potassium permanganate/Δ and phosphomolybdic acid stains (PMA)/Δ. For the removal of the solvents under reduced pressure Büchi R series rotatory evaporators were used. For precision weighting Sartorius Analytical Balance Practum 224-1S was used (±0.1 mg). *NMR spectra* were recorded at 25ºC on a Bruker AC-300 spectrometer (300 MHz for ^{1}H and 75.5 MHz for ^{13}C). ^{1}H NMR and $^{13}C\{^{1}H\}$ NMR chemical shifts (δ) are reported in ppm with the solvent (or TMS) resonance as the internal standard ($CHCl_3$: 7.26 ppm (^{1}H)) and ($CDCl_3$: 77.16 ppm (^{13}C). Data are reported as follows: chemical shift, multiplicity (d = doublet, t = triplet, q = quartet, br = broad, m = multiplet), coupling constants (Hz) and integration. *High resolution mass spectra (HRMS)* were recorded using an Aquity UPLC coupled to a QTOF mass spectrometer (SYNAPT G2 HDMS) using electrospray ionization (ESI^+ or ESI^-). *GC-MS analyses* were performed on an Agilent 7890A gas chromatograph coupled to an Agilent 5975 quadrupole mass spectrometer under electronic impact ionization (EI) 70 eV. *Melting points (M.p.)* were measured in a Stuart SMP30 apparatus in open capillary tubes and are uncorrected. The *enantiomeric excess (e.e.)* of the products was determined by High Performance Liquid Chromatography on a chiral stationary phase in a Waters chromatograph coupled to a Waters photodiode array detector. Daicel Chiralpak IA, IC, AD-H, AD-3 and OD-3 columns (0.46 x 25 cm) were used; specific conditions are indicated for each case. *Specific optical rotations* ($[\alpha]_D^{20}$) were measured at 20ºC on a Jasco P-2000 polarimeter with sodium lamp at 589 nm and a path of length of 1 dm. Solvent and concentration are specified in each case. *Infrared spectra (IR)* were measured in a Jasco FT/IR 4100 (ATR) in the interval between 4000 and 600 cm^{-1} with a 4 cm^{-1} resolution. X-ray data collections were performed in an Agilent Supernova diffractometer equipped with an Atlas CCD area detector, and a CuKα micro-focus source with multilayer optics (λ = 1.54184 Å, 250 µm FWHM beam size). The sample was kept at 150 K with an Oxford Cryosystems Cryostream 700 cooler. The quality of the crystals was checked under a polarizing miscroscope, and a suitable crystal or fragment was mounted on a Mitegen MicromountTM using Paratone N inert oil and transferred to the diffractometer.

[1](a) W. L. F. Armarego, C. L. L. Chai. *Purification of Laboratory Chemicals*, 7th ed.; Elsevier: Oxford, 2012 (b) D. B. G. Williams, M. Lawton. *J. Org. Chem*. **2010**, *75*, 8351.

2. Experimental procedures and spectral data

2.1. Optimization of the reaction conditions

Table SI-1. Optimization of the acid-catalyzed transannular (3+2) cycloaddition using **1a** as model substrate.[a]

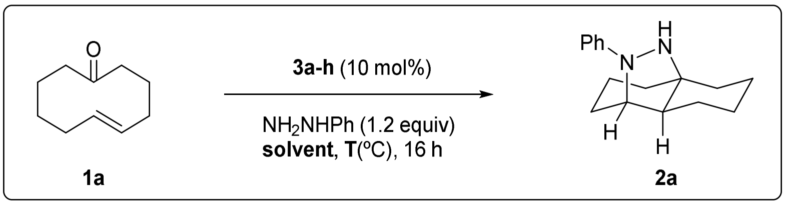

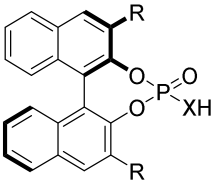

3a: R=2,4,6-(iPr)$_3$C$_6$H$_2$, XH=OH
3b: R=2,4,6-(iPr)$_3$C$_6$H$_2$, XH=NHTf
3c: R=9-anthryl, XH=OH
3d: R=9-phenanthryl, XH=OH
3e: R=2-naphtyl, XH=OH
3f: R=SiPh$_3$, XH=OH
3g: R=2,4,6-(C$_6$H$_{11}$)$_3$C$_6$H$_2$, XH=OH

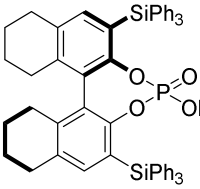

3h

Entry	Catalyst	Solvent	Additives	Conc. (*M*)	T (ºC)	Conv (%) [IY (%)][b]	e.e. (%)[c]
1	**(PhO)$_2$P(O)OH**	toluene	-	0.1	rt	<5[d]	--
2	**(PhO)$_2$P(O)OH**	toluene	-	0.1	50	99 [72]	--
3	**3a**	C_6H_5Cl	-	0.1	50	99	0
4	**3c**	C_6H_5Cl	-	0.1	50	61	45
5	**3f**	C_6H_5Cl	-	0.1	50	99	21
6	**3c**	C_6H_5Cl	4Å MS	0.1	50	81	17
7	**3f**	toluene	-	0.1	50	99 [92]	88
8	**3a**	toluene	-	0.1	50	99	85
9	**3b**	toluene	-	0.1	50	55 [39]	25
10	**3c**	toluene	-	0.1	50	99	33
11	**3d**	toluene	-	0.1	50	99	23
12	**3e**	toluene	-	0.1	50	99	17
13	**3f**	THF	-	0.1	50	42	53
14	**3f**	$CHCl_3$	-	0.1	50	33	75
15	**3f**	EtOH	-	0.1	50	68	16
16	**3f**	CH_2Cl_2	-	0.1	50	99 [91]	89
17	**3f**	toluene	-	0.1	40	46	79
18	**3f**	toluene	-	0.1	30	25	n.d.[e]
19	**3f**	toluene	-	0.05	50	99	71
20	**3f**	toluene	-	1	50	95	49
21	**3h**	toluene	-	0.1	50	99 [90]	90
22	**3g**	toluene	-	0.1	rt	<5[d]	n.d.[e]
23	**3g**	toluene	-	0.1	50	99 [99]	98
24[f]	**3g**	toluene	-	0.1	50	99 [99]	96

[a] Reactions were performed with 0.15 mmol of **1a**, NH$_2$NHPh (1.2 equiv), **3a**-**h** (10 mol%) for 16 h. [b] Conversion was calculated by ^{1}H-NMR using 1,3,5-trimethoxybenzene as internal standard; [IY (%)] = isolated yield after flash column chromatography purification. [c] e.e. was calculated by HPLC on chiral stationary phase after derivatization into the corresponding benzoyl hydrazide. [d] Starting material was recovered as the corresponding hydrazone. [e] n.d. = not determined. [f] 5 mol% of catalyst was used.

2.2. Synthesis of starting materials

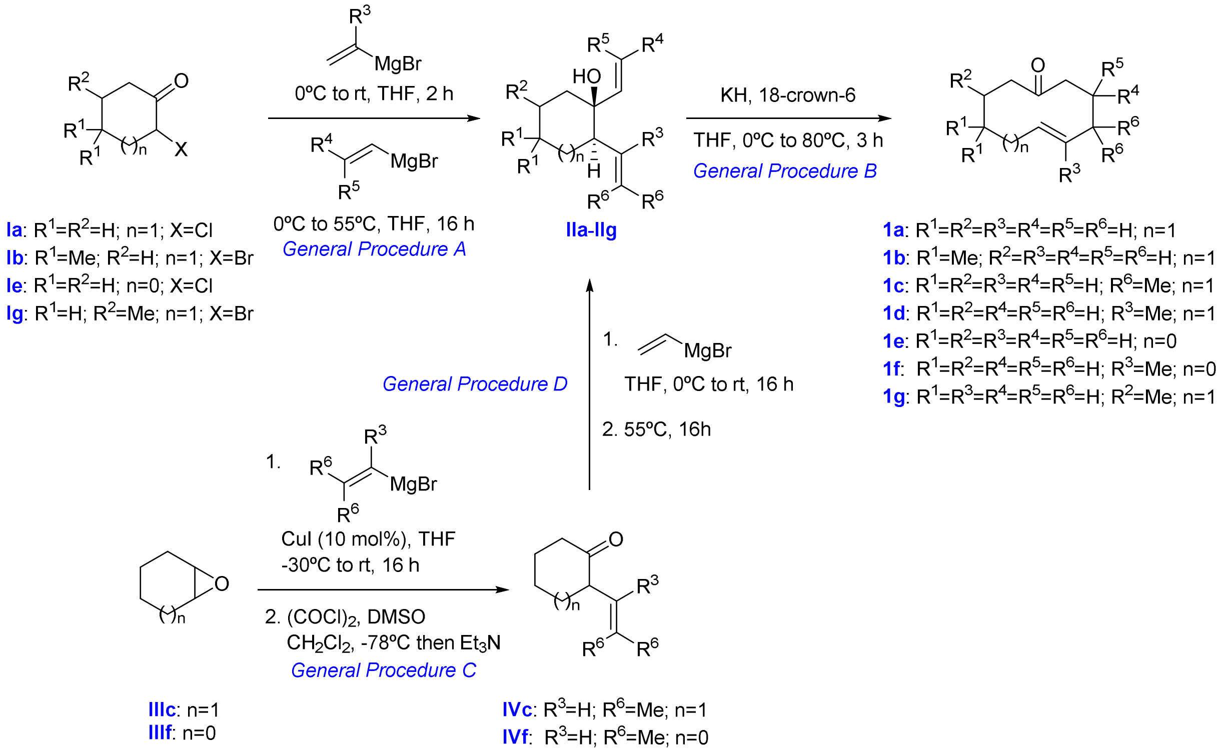

Scheme SI-1. General overview of the synthesis of starting materials **1a-1g**.

Ia, **Ie**, **IIIc** and **IIIf** were commercially available and used without further purification. Compounds **Ib** and **Ig** were synthetized according to procedures previously described in the literature and used without further purification.[2]

General Procedure A for the synthesis of substrates **IIa, IIb, IId, IIe** and **IIg**: These compounds were prepared following the procedure described in the literature. Spectroscopic data of substrates **IIa** and **IId** were consistent with those reported in the literature.[3] A solution of 2-chlorocycloalkanone (1 equiv) in THF and vinylmagnesium bromide solution (0.5-1 *M* in THF, 1.25 equiv) were added dropwise to THF at 0ºC over 1 h under argon atmosphere. After that time, the reaction was left stirring for 1 h additional before the addition of the Grignard reagent in THF (0.5-1 *M* in THF, 1.25 equiv) over a period of 10 minutes. The reaction was allowed to warm up to 55ºC, using an aluminium heating block, and kept stirring overnight. Upon completion, saturated aqueous NH_4Cl was added, the phases separated and the aqueous phase was extracted with Et_2O (3 x 10 mL). The combined organic layers were washed with brine, dried over Na_2SO_4, filtered and the solvent evaporated under reduced pressure. The obtained crude was purified by column chromatography.

[2] (a) B. Riss, M. Garreau, P. Fricero, P. Podsiadly, N. Berton, S. Buchter. *Tetrahedron* **2017**, *73*, 3202. (b) C. Faeh, H. Kuehne, T. Luebbers, P. Mattei, C. Maugeais, P. Pflieger. (2007). Preparation of heteroaryl and benzyl amide compounds as CETP inhibitors for treating dyslipidemia and other diseases. (U.S. Pat. Appl. Publ. No. 20070185113).

[3] (a) K. Tomooka, T. Ezawa, H. Inoue, K. Uehara, K. Igawa. *J. Am. Chem. Soc*. **2011**, *133*, 1754. (b) G. A. Molander, B. Czakó, M. Rheam. *J. Org. Chem.* **2007**, *72*, 1755. (c) T. Kato, H. Kondo, M. Nishino, M. Tanaka, G. Hata, A. Miyake. *Bull. Chem. Soc. Jpn*. **1980**, *53*, 2958.

General Procedure B for the synthesis of substrates **1a**-**1g**: These compounds were prepared following the procedure described in the literature. Spectroscopic data of substrates **1a**, **1d** and **1f** were consistent with those reported in the literature.[3,4a] An oven-dried round-bottom flask was charged with KH (1.3 equiv), dissolved in THF and cooled to 0ºC under argon atmosphere. Then, a solution of 18-crown-6 (2 equiv) and a solution of divinyl alcohol (1 equiv) were added dropwise *via* cannula. The reaction was allowed to warm up to 80ºC, using an aluminium heating block, and it was left under reflux for 2-3 h. Upon completion, saturated aqueous NH_4Cl was added, the phases separated and the aqueous phase was extracted with Et_2O (3 x 10 mL). The combined organic layers were washed with brine, dried over Na_2SO_4, filtered and the solvent evaporated under reduced pressure. The obtained crude was purified by column chromatography.

General Procedure C for the synthesis of substrates **IVc** and **IVf**: These compounds were prepared following the procedure described in the literature. Spectroscopic data of **IVf** were consistent with those reported in the literature.[4] An oven-dried round-bottom flask was charged with CuI (0.15 equiv), dissolved in THF and cooled to -30ºC under argon atmosphere. To the formed suspension, the corresponding Grignard reagent in THF was added (1.5 equiv) over a period of 30 min and left stirring for additional 10 min at -30ºC. After the mentioned time, *meso*-epoxide (1 equiv) was added dropwise over 10 min and the reaction mixture was allowed to gradually warm up to room temperature and left stirring overnight. Upon reaction completion, the crude solution was cooled to 0ºC and quenched with saturated aqueous NH_4Cl and diluted with H_2O, the phases separated and the aqueous phase was extracted with Et_2O (3 x 10 mL). The combined organic layers were washed with brine, dried over Na_2SO_4, filtered and the solvent evaporated under reduced pressure. The crude was used without further purification as follows. To a solution of oxalyl chloride (1.2 equiv) in CH_2Cl_2 at -78ºC was added dropwise a solution of DMSO (2.5 equiv) in CH_2Cl_2 and stirred at the mentioned temperature for 10 min. Then, a solution of the crude alcohol (1 equiv) in CH_2Cl_2 was added dropwise and the crude solution was stirred for 1 h at -78ºC. The reaction was quenched by the addition of Et_3N (5 equiv) and immediately warmed up to room temperature. The contents were diluted with H_2O, the phases separated and the aqueous phase was extracted with Et_2O (3 x 10 mL). The combined organic layers were washed with brine, dried over Na_2SO_4, filtered and the solvent evaporated under reduced pressure. The obtained crude was purified by column chromatography.

General Procedure D for the synthesis of substrates **IIc and IIf**: These compounds were prepared following the procedure described in the literature. Spectroscopic data of **IIf** were consistent with those reported in the literature.[4] A solution of the unsaturated ketone (1 equiv) in THF was cooled down to 0ºC before the dropwise addition of the Grignard reagent in THF (0.5-1 *M* in THF, 1.25 equiv) under argon atmosphere. The reaction was allowed to warm up to room temperature and kept stirring overnight. Upon completion, saturated aqueous NH_4Cl was added, the phases separated and the aqueous phase was extracted with Et_2O (3 x 10 mL). The combined organic layers were washed with brine, dried over Na_2SO_4, filtered and the solvent evaporated under reduced pressure. The obtained crude was purified by column chromatography.

[4] (a) N. S. Rajapaksa, E. N. Jacobsen. *Org. Lett*. **2013**, *15*, 4238. (b) K. Tomooka, T. Ezawa, H. Inoue, K. Uehara, K. Igawa. *J. Am. Chem. Soc*. **2011**, *133*, 1754. (c) G. A. Molander, B. Czakó, M. Rheam. *J. Org. Chem.* **2007**, *72*, 1755.

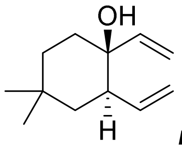

***rac*-(1*S*,2*R*)-4,4-dimethyl-1,2-divinylcyclohexan-1-ol (IIb)**

Following the *General Procedure A*, compound **IIb** (0.38 g, 2.08 mmol) was isolated after 16 h at 55ºC by flash chromatography (petroleum ether/EtOAc 9:1) in 36% yield as a colourless oil starting from **Ib** (1.19 g, 5.80 mmol), vinylmagnesium bromide solution (0.6 *M* in THF, 14.50 mmol) and THF (10 mL). ^{1}H-NMR (δ, ppm) (300 MHz, $CDCl_3$): 5.97-5.77 (m, 2H), 5.22 (dd, *J* = 17.3, 1.4 Hz, 1H), 5.13-4.98 (m, 3H), 2.34-2.23 (m, 1H), 1.73-1.43 (m, 4H), 1.21 (dt, *J* = 12.4, 2.5 Hz, 2H), 0.98 (s, 3H), 0.94 (s, 3H). ^{13}C-NMR (δ, ppm) (75.5 MHz, $CDCl_3$): 145.9, 138.7, 116.1, 111.9, 72.8, 43.9, 38.5, 34.1, 33.7, 33.0, 30.0, 24.0. IR (ATR) cm^{-1}: 3486, 2950, 2927, 1364, 1219, 913, 772. MS (EI, 70 eV) m/z (%): 195 (4), 181 (15), 180 (M^+, 11), 179 (22), 178 (10), 177 (11), 176 (5), 174 (4), 164 (12), 163 (87), 162 (100), 161 (23), 160 (15), 159 (6), 158 (11), 156 (5). HRMS (ESI) m/z: $[M-H_2O+H]^+$ Calcd for $C_{12}H_{19}$ 163.1487; Found 163.1490.

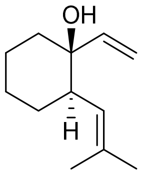

***rac*-(1*S*,2*R*)-2-(2-methylprop-1-en-1-yl)-1-vinylcyclohexan-1-ol (IIc)**

Following the *General Procedure D*, compound **IIc** (0.56 g, 3.11 mmol) was isolated after 16 h at room temperature by flash chromatography (petroleum ether/EtOAc 19:1) in 38% yield as a pale green oil starting from **IVc** (1.23 g, 8.08 mmol), vinylmagnesium bromide solution (0.7 *M* in THF, 9.70 mmol) and THF (15 mL). ^{1}H-NMR (δ, ppm) (300 MHz, $CDCl_3$): 5.80 (dd, *J* = 17.4, 10.8 Hz, 1H), 5.15-4.86 (m, 3H), 2.26-2.09 (m, 1H), 1.63 (s, 3H), 1.53 (s, 3H), 1.50-1.46 (m, 3H), 1.40 (ddd, *J* = 10.5, 6.1, 2.4 Hz, 4H), 1.24 (ddt, *J* = 12.3, 8.3, 3.7 Hz, 1H). ^{13}C-NMR (δ, ppm) (75.5 MHz, $CDCl_3$): 146.0, 132.0, 124.9, 111.1, 73.7, 44.2, 37.7, 28.3, 25.8, 25.3, 21.3, 18.2. IR (ATR) cm^{-1}: 3480, 2927, 2853, 1444, 1375, 966, 916. MS (EI, 70 eV) m/z (%): 179 (10), 178 (6), 177 (14), 176 (4), 175 (4), 164 (16), 163 (100), 162 (63), 161 (26), 160 (15), 159 (5), 158 (6), 156 (4), 119 (6). HRMS (ESI) m/z: $[M+HCOOH-H]^-$ Calcd for $C_{13}H_{21}O_3$ 225.1491; Found 225.1488.

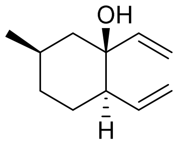

(1*S*,2*R*,5*R*)-5-methyl-1,2-divinylcyclohexan-1-ol (IIg)

Following the *General Procedure A*, compound **IIg** (315.70 mg, 1.90 mmol) was isolated after 3 h at 80ºC by flash chromatography (petroleum ether/EtOAc 8:2) in 34% yield as a pale brown oil starting from **Ig** (0.94 g, 5.65 mmol), vinylmagnesium bromide solution (0.8 *M* in THF, 13.00 mmol) and THF (7.8 mL). ^{1}H-NMR (δ, ppm) (300 MHz, $CDCl_3$): 5.88 (ddd, *J* = 17.3, 10.7, 5.4 Hz, 2H), 5.26-4.94 (m, 4H), 2.11-2.01 (m, 1H), 1.89-1.59 (m, 5H), 1.45 (s, 1H), 1.13 (dd, *J* = 13.7, 12.1 Hz, 1H), 1.04-0.92 (m, 1H), 0.89 (d, *J* = 6.4 Hz, 3H). ^{13}C-NMR (δ, ppm) (75.5 MHz, $CDCl_3$): 145.9, 138.7, 116.2, 111.7, 73.8, 47.7, 46.5, 34.2, 27.3, 25.7, 22.1. $[\alpha]_D^{20}$: +173.6 (*c* 0.9, CH_2Cl_2). IR (ATR) cm^{-1}: 3502, 2989, 1275, 1260, 763, 750. MS (EI, 70 eV) m/z (%): 164 (5), 150 (12), 149 (97), 148 (100), 147 (15), 146 (9), 144 (3), 66 (6), 55 (9), 54 (6), 53 (7), 51 (6). HRMS (ESI) m/z: $[M+H]^+$ Calcd for $C_{11}H_{19}O$ 167.1436; Found 167.1432.

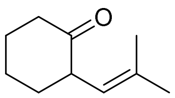

2-(2-methylprop-1-en-1-yl)cyclohexan-1-one (IVc)

Following the *General Procedure C*, compound **IVc** (1.23 g, 8.08 mmol) was isolated after 2 h at

room temperature by flash chromatography (petroleum ether/EtOAc 8:2) in 82% yield as a yellow oil starting from **IIIc** (1.52 g, 9.89 mmol), oxalyl chloride (1.01 mL, 11.87 mmol), DMSO (1.76 mL, 24.73 mmol) and CH_2Cl_2 (38 mL). ^{1}H-NMR (δ, ppm) (300 MHz, $CDCl_3$): 5.28-5.19 (m, 1H), 3.11 (td, *J* = 9.5, 6.1 Hz, 1H), 2.37 (dt, *J* = 9.5, 3.1 Hz, 1H), 2.22 (dt, *J* = 14.4, 3.6 Hz, 1H), 1.99-1.88 (m, 2H), 1.79 (dd, *J* = 7.8, 3.3 Hz, 1H), 1.68 (s, 3H), 1.65-1.61 (m, 1H), 1.58-1.51 (m, 1H), 1.52 (s, 3H), 1.49-1.45 (m, 1H). ^{13}C-NMR (δ, ppm) (75.5 MHz, $CDCl_3$): 211.5, 134.1, 121.6, 50.2, 41.5, 34.7, 27.7, 25.8, 24.2, 17.9. IR (ATR) cm^{-1}: 2925, 1275, 1260, 764, 750. MS (EI, 70 eV) m/z (%): 153 (3), 138 (3), 137 (100), 136 (12), 135 (6), 134 (3), 117 (3), 92 (3). HRMS (ESI) m/z: $[M+H]^+$ Calcd for $C_{10}H_{17}O$ 153.1279; Found 153.1282.

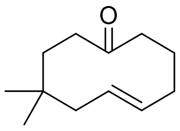

(*E*)-8,8-dimethylcyclodec-5-en-1-one (1b)

Following the *General Procedure B*, compound **1b** (226.50 mg, 1.26 mmol) was isolated after 3 h at 80ºC by flash chromatography (petroleum ether/EtOAc 19:1) in 63% yield as a white crystalline solid starting from **IIb** (360.0 g, 2.00 mmol), KH (347 mg, 2.60 mmol), 18-crown-6 (1.06 g, 4.00 mmol) and THF (31 mL). ^{1}H-NMR (δ, ppm) (300 MHz, $CDCl_3$): 5.51-5.40 (m, 1H), 5.09 (dd, *J* = 15.0, 7.4 Hz, 1H), 2.79-2.68 (m, 1H), 2.29-2.02 (m, 6H), 1.87-1.67 (m, 4H). ^{13}C-NMR (δ, ppm) (75.5 MHz, $CDCl_3$): 212.6, 135.6, 128.1, 46.2, 43.0, 40.5, 34.2, 34.1, 34.0, 33.6, 28.3, 24.3. IR (ATR) cm^{-1}: 2942, 2925, 1705, 1364, 1106, 986, 772. MS (EI, 70 eV) m/z (%): 181 (5), 180 (M^+, 11), 179 (10), 178 (5), 177 (4), 164 (14), 163 (100), 162 (65), 161 (18), 160 (16), 158 (5). HRMS (ESI) m/z: $[M-H_2O+H]^+$ Calcd for $C_{12}H_{19}$ 163.1487; Found 163.1485. M.p.: 41-42 ºC (EtOAc/hexane).

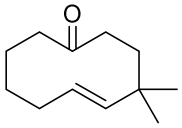

(*E*)-4,4-dimethylcyclodec-5-en-1-one (1c)

Following the *General Procedure B*, compound **1c** (368.00 mg, 2.04 mmol) was isolated after 3 h at 80ºC by flash chromatography (petroleum ether/EtOAc 9:1) in 66% yield as a colourless oil starting from **IIc** (560.00 mg, 3.11 mmol), KH (540 mg, 4.04 mmol), 18-crown-6 (1.64 g, 6.22 mmol) and THF (48 mL). ^{1}H-NMR (δ, ppm) (300 MHz, $CDCl_3$): 5.31-5.04 (m, 2H), 2.53-1.25 (m, 12H), 1.01 (s, 6H). ^{13}C-NMR (δ, ppm) (75.5 MHz, $CDCl_3$): 213.0, 143.0, 126.5, 45.7, 41.8, 39.2, 36.8, 33.3, 28.6, 22.1. IR (ATR) cm^{-1}: 2954, 2924, 1707, 1445, 1364, 1195, 989. MS (EI, 70 eV) m/z (%): 181 (4), 180 (M^+, 7), 179 (7), 178 (5), 177 (6), 164 (9), 163 (70), 162 (100), 161 (32), 160 (17), 158 (7), 156 (4). HRMS (ESI) m/z: $[M-H_2O+H]^+$ Calcd for $C_{12}H_{19}$ 163.1487; Found: 163.1491.

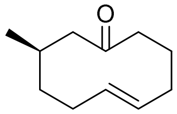

(*R*,*E*)-9-methylcyclodec-5-en-1-one (1g)

Following the *General Procedure B*, compound **1g** (158.90 mg, 0.96 mmol) was isolated after 2 h at 80ºC by flash chromatography (petroleum ether/EtOAc 9:1) in 51% yield as a colourless oil starting from **IIg** (315.7 mg, 1.90 mmol), KH (330 mg, 2.50 mmol), 18-crown-6 (1.0 g, 3.80 mmol) and THF (33 mL). ^{1}H-NMR (δ, ppm) (300 MHz, $CDCl_3$): 5.29 (ddd, *J* = 14.0, 10.2, 3.3 Hz, 1H), 5.10 (ddd, *J* = 15.1, 10.5, 3.5 Hz, 1H), 2.53 (dd, *J* = 16.7, 8.6 Hz, 1H), 2.17 (tt, *J* = 14.8, 6.0 Hz, 7H), 1.93 (dtd, *J* = 13.5, 11.2, 10.1, 2.8 Hz, 1H), 1.81-1.70 (m, 2H), 1.63 (ddt, *J* = 14.2, 5.6, 2.6 Hz, 1H), 1.17 (q, *J* = 12.0, 11.4 Hz, 1H), 0.92 (d, *J* = 7.1 Hz, 3H). ^{13}C-NMR (δ, ppm) (75.5 MHz, $CDCl_3$): 212.0, 134.8, 130.8, 53.8, 42.8, 37.3, 34.1, 32.2, 28.4, 27.8, 24.9. $[\alpha]_D^{20}$: +522.7 (*c* 0.3, CH_2Cl_2). IR (ATR) cm^{-1}: 2989, 1275, 1260, 763, 750. MS (EI, 70 eV) m/z (%): 167 (17),

166 (M^+, 14), 165 (23), 150 (15), 149 (100), 148 (86), 147 (14), 146 (8), 145 (10), 143 (6). HRMS (ESI) m/z: $[M+H]^+$ Calcd for $C_{11}H_{19}O$ 167.1436; Found 167.1436.

2.3. *General Procedure I* for the enantioselective transannular (3+2) cycloaddition. To an oven-dried screw-top vial, equipped with a magnetic stirring bar, the enone **1a-g** (1 equiv, 0.15 mmol), catalyst (*S*)-**3g** (5 mol%, 0.008 mmol) and the corresponding hydrazine (1.2 equiv, 0.18 mmol) were added followed by dry toluene (1.5 mL). The vial was sealed with a plastic cap and the resulting mixture was left stirring at the indicated temperature in each case, using an aluminium heating block, until consumption of the starting material was observed by TLC. The solvent was evaporated under reduced pressure and the NMR yield was calculated through comparison to an internal standard (1,3,5-trimethoxybenzene). Purification by column chromatography afforded the desired products. Racemic standards for HPLC separation were prepared under the same reaction conditions, using diphenylphosphoric acid (DPP) (0.015 mmol) as catalyst.

2.4. *General Procedure II* for the benzoylation of 2a, **2b**, **2d**, **2i**, **2j**, **2k**, **2l** and **2m.** To a cooled solution of **2a**, **2b**, **2d**, **2i**, **2j**, **2k**, **2l** or **2m** (1.1 equiv) in CH_2Cl_2 (2 mL/mmol) at 0ºC, triethylamine (1.5 equiv) was added dropwise followed by the addition of benzoyl chloride (1 equiv). The reaction mixture was left overnight stirring at room temperature. Upon reaction completion, the crude was diluted with CH_2Cl_2, washed with a solution of HCl 1 *N*, dried over Na_2SO_4, filtered and the solvent was evaporated under reduced pressure. Purification by column chromatography afforded the desired products.

2.5. *General Procedure III* for the acetylation of 2c, **2e** and **2f.** To a cooled solution of **2c**, **2e** or **2f** (1 equiv) and DMAP (20 mol%) in DCM (0.1 *M*) at 0ºC, pyridine (10 equiv) was added dropwise followed by the addition of acetyl chloride (10 equiv). The reaction mixture was left 2 h stirring at room temperature. Upon reaction completion, the crude was diluted with CH_2Cl_2, washed with a solution of HCl 1 *N*, dried over Na_2SO_4, filtered and the solvent was evaporated under reduced pressure. Purification by column chromatography afforded the desired products.

2.6. Synthesis of enantioenriched 1,3-diamines. *General Procedure* for the reductive cleavage of 4a-g. To a two-neck round-bottom flask equipped with a magnetic stir bar, **4a-g** (15.0 mg, 0.062 mmol), Raney Ni (~200 mg, washed three times with EtOH before addition) and EtOH (4 mL). The system was filled with H_2 (balloon, 1 atm), purged (vacuum/H_2 cycles) and it was refluxed for 40 min at 80ºC, using an aluminium heating block. After reaction completion, the mixture was filtered through a short pad of Celite® and extensively washed with EtOH. Purification by column chromatography afforded the desired products.[5]

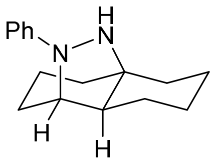

(1*S*,4a*R*,8a*S*)-10-phenyloctahydro-2*H*-1,4a-epidiazanonaphthalene (2a)

Following the *General Procedure I*, **2a** (36.10 mg, 0.15 mmol) was isolated after 16 h at 50ºC by flash chromatography (petroleum ether/EtOAc 19:1) in 99% yield as a colourless oil starting from **1a** (22.80 mg, 0.15 mmol), phenylhydrazine (18 µL, 0.18 mmol), (*S*)-**3g** (7.50 mg, 0.008 mmol) and toluene (1.5 mL, 0.1 *M*). Reaction performed at 1.00 mmol scale: Following the *General Procedure I*, **2a** (201.5 mg, 0.83 mmol) was isolated after 10 h at 50ºC by flash chromatography (petroleum ether/EtOAc 19:1) in 83% yield as a colourless oil starting from **1a** (152 mg, 1.00 mmol), phenylhydrazine (0.12 mL, 1.2 mmol), (*S*)-**3g** (50 mg, 0.05 mmol) and toluene (10 mL, 0.1 *M*). ^{1}H-NMR (δ, ppm) (300 MHz, $CDCl_3$): 7.19 (dd, *J* = 8.7, 7.3 Hz, 2H), 6.90

[5] X. Wu, B. Liu, Y. Zhang, M. Jeret, H. Wang, P. Zheng, S. Yang, B.-A. Song, Y. Chi. R. *Angew. Chem. Int. Ed*. **2016**, *55*, 12280

(d, J = 8 Hz, 2H), 6.65 (tt, J = 7.2, 11.2 Hz, 1H), 4.06 (br s, 1H), 3.88 (d, J = 4.4 Hz, 1H), 2.21-2.10 (m, 1H), 1.89-1.73 (m, 2H), 1.71-1.50 (m, 5H), 1.44-1.33 (m, 5H), 1.89-1.73 (m, 2H). ^{13}C-NMR (δ, ppm) (75.5 MHz, $CDCl_3$): 149.2, 128.7, 116.2, 113.0, 62.1, 59.5, 49.8, 40.4, 31.0, 28.0, 25.8, 24.5, 21.7, 18.8. The enantiomeric excess (e.e.) was determined by HPLC after derivatization of the corresponding product following the *General Procedure II*. $[\alpha]_D^{20}$: -12.0 (*c* 1.0, CH_2Cl_2). IR (ATR) cm^{-1}: 2928, 1590, 1219, 770. MS (EI, 70 eV) m/z (%): 243 (11), 242 (M^+, 60), 21 (5), 213 (4), 200 (15), 199 (100), 198 (4), 171 (4), 157 (6), 135 (6), 134 (8), 130 (6), 117 (5), 108 (4), 107 (6), 104 (7), 94 (4), 93 (8), 92 (4), 91 (11), 81 (4), 79 (10), 78 (6), 77 (37), 67 (7), 65 (6), 55 (4), 53 (4), 51 (8). HRMS (ESI) m/z: $[M+H]^+$ Calcd for $C_{16}H_{23}N_2$ 243.1861; Found 243.1866.

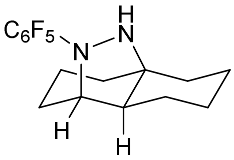

(1*S*,4a*R*,8a*S*)-10-(perfluorophenyl)octahydro-2*H*-1,4a-epidiazano-naphthalene (2b)

Following the *General Procedure I*, **2b** (48.00 mg, 0.14 mmol) was isolated after 16 h at 50ºC by flash chromatography (petroleum ether/EtOAc 19:1) in 96% yield as a pale orange oil starting from **1a** (22.80 mg, 0.15 mmol), pentafluorophenylhydrazine (35.60 mg, 0.18 mmol), (*S*)-**3g** (7.50 mg, 0.008 mmol) and toluene (1.5 mL, 0.1 *M*). ^{1}H-NMR (δ, ppm) (300 MHz, $CDCl_3$): 4.37 (br s, 1H), 3.90 (s, 1H), 1.85-1.68 (m, 5H), 1.59-1.53 (m, 5H), 1.49-1.37 (m, 3H), 1.28-1.18 (m, 2H). ^{13}C-NMR (δ, ppm) (75.5 MHz, $CDCl_3$): 66.4, 59.8, 49.0, 40.0, 30.9, 30.1, 25.7, 24.4, 21.3, 18.8, (Fluor containing C_{arom} do not relax). ^{19}F-NMR (δ, ppm) (282 MHz, $CDCl_3$): -150.86 (m), -164.66 (m), -171.43 (tt, J = 22.6, 5.4 Hz). The enantiomeric excess (e.e.) was determined by HPLC after derivatization of the corresponding product following the *General Procedure II*. $[\alpha]_D^{20}$: +29.8 (*c* 0.7, CH_2Cl_2). IR (ATR) cm^{-1}: 2934, 1515, 1491, 1051, 749. MS (EI, 70 eV) m/z (%): 345 (2), 334 (2), 333 (18), 332 (M^+, 100), 331 (77), 330 (26), 329 (4), 328 (2), 165 (3), 136 (7), 135 (42), 134 (21), 133 (3), 133 (3). HRMS (ESI) m/z: $[M+H]^+$ Calcd for $C_{16}H_{18}N_2F_5$ 333.1390; Found 333.1393.

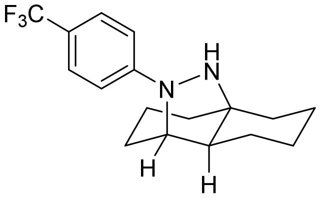

(1*S*,4a*R*,8a*S*)-10-(4-(trifluoromethyl)phenyl)octahydro-2*H*-1,4a-epidiazanonaphthalene (2c)

Following the *General Procedure I*, **2c** (44.20 mg, 0.14 mmol) was isolated after 16 h at 50ºC by flash chromatography (petroleum ether/EtOAc 19:1) in 95% yield as a yellow oil starting from **1a** (22.80 mg, 0.15 mmol), 4-(trifluoromethyl)phenylhydrazine (31.70 mg, 0.18 mmol), (*S*)-**3g** (7.50 mg, 0.008 mmol) and toluene (1.5 mL, 0.1 *M*). ^{1}H-NMR (δ, ppm) (300 MHz, $CDCl_3$): 7.40 (d, J = 8.6 Hz, 2H), 6.88 (br s, 2H), 4.12 (s, 1H), 3.91 (d, J = 4.3 Hz, 1H), 2.13 (dd, J = 7.7, 4.3 Hz, 1H), 1.81 (dd, J = 11.2, 4.8 Hz, 2H), 1.76-1.54 (m, 5H), 1.47-1.34 (m, 5H), 1.26-1.16 (m, 2H). ^{13}C-NMR (δ, ppm) (75.5 MHz, $CDCl_3$): 151.0, 126.0, 117.6, 117.4 (q, J = 32.3 Hz), 112.0, 62.2, 59.7, 49.7, 40.1, 30.8, 27.7, 25.7, 24.4, 21.6, 18.7. ^{19}F-NMR (δ, ppm) (282 MHz, $CDCl_3$): -60.66. The enantiomeric excess (e.e.) was determined by HPLC after derivatization of the corresponding product following the *General Procedure III*. $[\alpha]_D^{20}$: +14.9 (*c* 0.9, CH_2Cl_2). IR (ATR) cm^{-1}: 2857, 1611, 1321, 1105, 750, 763. MS (EI, 70 eV) m/z (%): 311 (19), 310 (M^+, 100), 309 (85), 308 (10), 292 (5), 291 (21). HRMS (ESI) m/z: $[M+H]^+$ Calcd for $C_{17}H_{22}N_2F_3$ 311.1735; Found 311.1734.

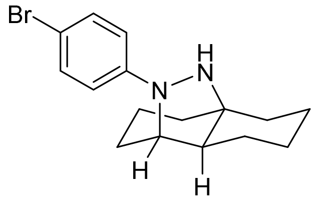

(1*S*,4a*R*,8a*S*)-10-(4-bromophenyl)octahydro-2*H*-1,4a-epidiazano-naphthalene (2d)

Following the *General Procedure I*, **2d** (40.60 mg, 0.13 mmol) was isolated after 20 h at 50ºC by flash chromatography (petroleum ether/EtOAc 19:1) in 84% yield as a brown oil starting from **1a** (22.80 mg, 0.15 mmol), 4-bromophenylhydrazine[6] (33.7 mg, 0.18 mmol), (*S*)-**3g** (7.50 mg, 0.008 mmol) and toluene (1.5 mL, 0.1 *M*). ^{1}H-NMR (δ, ppm) (300 MHz, $CDCl_3$): 7.28 (d, *J* = 8.8 Hz, 2H, 2 x C**H**$_{arom}$), 6.81 (d, *J* = 8.3 Hz, 2H), 4.06 (s, 1H), 3.85 (d, *J* = 4.3 Hz, 1H), 2.12 (dt, *J* = 8.7, 4.2 Hz, 1H), 1.83-1.52 (m, 6H), 1.48-1.33 (m, 6H), 1.29-1.21 (m, 2H). ^{13}C-NMR (δ, ppm) (75.5 MHz, $CDCl_3$): 148.3, 131.3, 114.9, 108.0, 62.4, 59.7, 49.9, 40.3, 30.9, 27.8, 25.8, 24.4, 21.6, 18.7. The enantiomeric excess (e.e.) was determined by HPLC after derivatization of the corresponding product following the *General Procedure II*. $[\alpha]_D^{20}$: +19.7 (*c* 0.8, CH_2Cl_2). IR (ATR) cm^{-1}: 2929, 1587, 1484, 1259, 763, 750. MS (EI, 70 eV) m/z (%): 337 (5), 336 (11), 335 (13), 334 (13), 331 (10), 322 (31), 321 (80), 320 (M^+, 100), 319 (70), 318 (87), 317 (7), 311 (4), 304 (4), 184 (5), 183 (4), 170 (4), 164 (4), 151 (4), 147 (4), 144 (4), 136 (4), 135 (7), 134 (13). HRMS (ESI) m/z: $[M+H]^+$ Calcd for $C_{16}H_{20}N_2Br$ 319.0810; Found 319.0811.

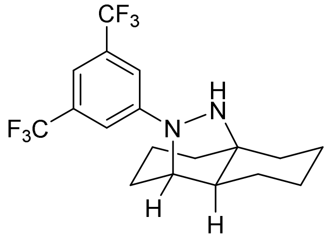

(1*S*,4a*R*,8a*S*)-10-(3,5-bis(trifluoromethyl)phenyl)octahydro-2*H*-1,4a-epidiazanonaphthalene (2e). Following the *General Procedure I*, **2e** (50.90 mg, 0.14 mmol) was isolated after 16 h at 50ºC by flash chromatography (petroleum ether/EtOAc 19:1) in 90% yield as a yellow oil starting from **1a** (22.80 mg, 0.15 mmol)), 3,5-bis(trifluoromethyl)phenylhydrazine (43.9 mg, 0.18 mmol), (*S*)-**3g** (7.50 mg, 0.008 mmol) and toluene (1.5 mL, 0.1 *M*). ^{1}H-NMR (δ, ppm) (300 MHz, $CDCl_3$): 7.09 (s, 1H), 4.15 (s, 1H), 3.94 (d, *J* = 4.3 Hz, 1H), 2.12-2.03 (m, 1H), 1.88-1.78 (m, 2H), 1.75-1.56 (m, 5H), 1.52-1.30 (m, 5H), 1.28-1.15 (m, 2H). ^{13}C-NMR (δ, ppm) (75.5 MHz, $CDCl_3$): 149.6, 125.6, 122.0 (br s), 112.3, 109.0, 62.6, 59.9, 49.7, 39.9, 30.7, 27.4, 25.7, 24.3, 21.5, 18.5. ^{19}F-NMR (δ, ppm) (282 MHz, $CDCl_3$): -62.97. The enantiomeric excess (e.e.) was determined by HPLC after derivatization of the corresponding product following the *General Procedure III*. $[\alpha]_D^{20}$: +9.9 (*c* 1.4, CH_2Cl_2). IR (ATR) cm^{-1}: 2937, 1615, 1275, 763, 750. MS (EI, 70 eV) m/z (%): 393 (1), 392 (1), 390 (1), 380 (2), 379 (22), 378 (M^+, 100), 377 (70), 376 (12), 375 (1), 373 (1), 135 (2), 134 (1). HRMS (ESI) m/z: $[M+H]^+$ Calcd for $C_{18}H_{21}N_2F_6$ 379.1609; Found 379.1608.

[6] The hydrazine was extracted from the corresponding hydrochloride following the procedure described: R. Dey, P. Kumar, P. Banerjee. *J. Org. Chem*. **2018**, *83*, 5438.

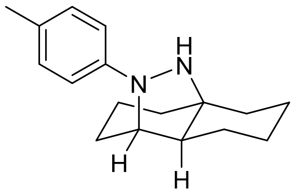

(1*S*,4a*R*,8a*S*)-10-(*p*-tolyl)octahydro-2*H*-1,4a-epidiazano-naphthalene (2f)

Following the *General Procedure I*, **2f** (38.50 mg, 0.15 mmol) was isolated after 20 h at 50ºC by flash chromatography (petroleum ether/EtOAc 19:1) in 99% yield as a brown oil starting from **1a** (22.80 mg, 0.15 mmol) , *p*-tolylhydrazine[6] (21.9 mg, 0.18 mmol), (*S*)-**3g** (7.50 mg, 0.008 mmol) and toluene (1.5 mL, 0.1 *M*). ^{1}H-NMR (δ, ppm) (300 MHz, $CDCl_3$): 7.01 (d, *J* = 8.2 Hz, 2H), 6.83 (d, *J* = 8.2 Hz, 2H), 4.03 (s, 1H), 3.85 (d, *J* = 4.3 Hz, 1H), 2.25 (s, 3H), 2.20-2.06 (m, 1H), 1.84-1.19 (m, 14H). ^{13}C-NMR (δ, ppm) (75.5 MHz, $CDCl_3$): 147.2, 129.2, 125.3, 113.2, 62.2, 59.5, 49.9, 40.5, 31.1, 28.1, 24.5, 21.7, 20.4, 18.9. The enantiomeric excess (e.e.) was determined by HPLC after derivatization of the corresponding product following the *General Procedure III*. $[\alpha]_D^{20}$: -14.4 (*c* 0.7, CH_2Cl_2). IR (ATR) cm^{-1}: 2927, 1592, 1275, 1260, 763, 750. MS (EI, 70 eV) m/z (%): 257 (20), 256 (M$^+$, 100), 255 (95), 254 (23), 213 (5), 212 (14). HRMS (ESI) m/z: [M+H]$^+$ Calcd for $C_{17}H_{25}N_2$ 257.2018; Found 257.2022.

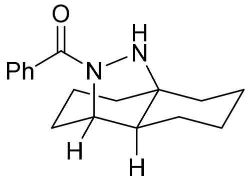

((1*S*,4a*R*,8a*S*)-octahydro-2*H*-1,4a-epidiazanonaphthalen-10-yl)(phenyl)methanone (2h)

Following the *General Procedure I*, **2h** (34.40 mg, 0.13 mmol) was isolated after 16 h at 50ºC by flash chromatography (petroleum ether/EtOAc 2:8) in 85% yield as a colourless oil starting from **1a** (22.80 mg, 0.15 mmol), benzoylhydrazine (24.5 mg, 0.18 mmol), (*S*)-**3g** (7.50 mg, 0.008 mmol) and toluene (1.5 mL, 0.1 *M*). ^{1}H-NMR (δ, ppm) (300 MHz, $CDCl_3$): 7.46-7.40 (m, 2H), 7.34-7.30 (m, 3H), 5.30 (br s, 1H), 3.70 (d, *J* = 4.5 Hz, 1H), 1.97-1.84 (m, 1H), 1.82-1.73 (m, 1H), 1.72-1.55 (m, 5H), 1.46-1.25 (m, 6H), 1.20-1.02 (m, 1H), 0.88-0.70 (m, 1H). ^{13}C-NMR (δ, ppm) (75.5 MHz, $CDCl_3$): 166.9, 135.9, 130.0, 128.3, 128.1, 127.9, 127.2, 64.8, 59.8, 49.3, 39.6, 31.5, 30.5, 25.4, 24.0, 21.1, 19.2. The enantiomeric excess (e.e.) was determined by HPLC using a CHIRALPAK® AD-H column (hexane/*i*-PrOH 90:10, 1 mL/min, 250 nm, 25ºC); t_r (major) = 18.75 min, t_r (minor) = 16.79 min (0% ee). IR (ATR) cm^{-1}: 3235, 2929, 1613, 1275, 1261, 764, 750. MS (EI, 70 eV) m/z (%): 271 (17), 270 (M$^+$, 29), 269 (25), 166 (16), 165 (100), 163 (8), 137 (12), 136 (69), 135 (39), 134 (21), 133 (8), 104 (20). HRMS (ESI) m/z: [M+H]$^+$ Calcd for $C_{17}H_{23}N_2O$ 271.1810; Found 271.1818.

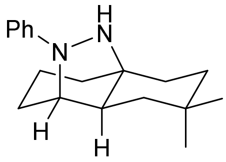

(1*S*,4a*S*,8a*S*)-7,7-dimethyl-10-phenyloctahydro-2*H*-1,4a-epidiazano-naphthalene (2i)

Following the *General Procedure I*, **2i** (40.40 mg, 0.15 mmol) was isolated after 20 h at 50ºC by flash chromatography (petroleum ether/EtOAc 19:1) in 99% yield as a yellow oil starting from **1b** (27.00 mg, 0.15 mmol), phenylhydrazine (18 µL, 0.18 mmol), (*S*)-**3g** (7.50 mg, 0.008 mmol) and toluene (1.5 mL, 0.1 *M*). ^{1}H-NMR (δ, ppm) (300 MHz, $CDCl_3$): 7.20 (dd, *J* = 8.7, 7.3 Hz, 2H), 6.90 (d, *J* = 8.0 Hz, 2H), 6.66 (tt, *J* = 7.2, 1.1 Hz, 1H), 4.04 (s, 1H), 3.86 (d, *J* = 4.3 Hz, 1H), 2.18 (ddd, *J* = 9.3, 4.0, 2.1 Hz, 1H), 1.94 (dd, *J* = 11.0, 6.6 Hz, 1H), 1.70-1.58 (m, 3H), 1.39-1.23 (m, 8H), 0.95 (s, 3H), 0.92 (s, 3H). ^{13}C-NMR (δ, ppm) (75.5 MHz, $CDCl_3$): 149.0, 128.7, 116.3,

113.1, 62.1, 58.8, 46.6, 40.1, 38.3, 34.1, 32.8, 30.1, 28.0, 27.5, 23.4, 18.8. The enantiomeric excess (e.e.) was determined by HPLC after derivatization of the corresponding product following the *General Procedure II*. [α]$_D^{20}$: -20.9 (*c* 0.3, CH_2Cl_2). IR (ATR) cm^{-1}: 2935, 1596, 1219, 770. MS (EI, 70 eV) m/z (%): 283 (4), 271 (16), 270 (M^+, 53), 269 (100), 268 (65), 266 (6), 265 (6), 248 (5), 225 (5), 224 (4), 205 (4), 203 (4), 177 (7), 163 (4), 162 (10), 161 (5), 159 (5), 145 (5), 142 (4), 127 (6), 126 (4), 93 (5). HRMS (ESI) m/z: $[M+H]^+$ Calcd for $C_{18}H_{27}N_2$ 271.2174; Found 271.2179.

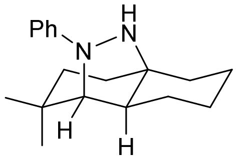

(1*R*,4a*R*,8a*S*)-2,2-dimethyl-10-phenyloctahydro-2*H*-1,4a-epidiazano-naphthalene (2j)

Following the *General Procedure I*, **2j** (38.10 mg, 0.14 mmol) was isolated after 20 h at 50ºC by flash chromatography (petroleum ether/EtOAc 19:1) in 94% yield as a colourless oil starting from **1c** (27.00 mg, 0.15 mmol), phenylhydrazine (18 μL, 0.18 mmol), (*S*)-**3g** (7.50 mg, 0.008 mmol) and toluene (1.5 mL, 0.1 *M*). ^{1}H-NMR (δ, ppm) (300 MHz, $CDCl_3$): 7.19 (dd, *J* = 8.8, 7.2 Hz, 2H), 7.03 (d, *J* = 8.1 Hz, 2H), 6.69 (tt, *J* = 7.2, 1.2 Hz, 1H), 3.39 (br s, 1H), 3.18 (s, 1H), 1.89 (dd, *J* = 11.8, 5.2 Hz, 1H), 1.79-1.60 (m, 4H), 1.55-1.26 (m, 6H), 1.21-1.09 (m, 1H), 1.07 (s, 3H), 1.01 (s, 3H), 0.94-0.80 (m, 1H). ^{13}C-NMR (δ, ppm) (75.5 MHz, $CDCl_3$): 154.0, 128.6, 116.8, 112.5, 76.3, 59.9, 44.4, 38.0, 36.3, 33.4, 32.4, 29.7, 25.9, 24.9, 24.4, 21.7. The enantiomeric excess (e.e.) was determined by HPLC after derivatization of the corresponding product following the *General Procedure II*. [α]$_D^{20}$: -202.6 (*c* 0.4, CH_2Cl_2). IR (ATR) cm^{-1}: 2923, 1593, 1219, 775. MS (EI, 70 eV) m/z (%): 297 (4), 285 (7), 284 (7), 283 (5), 282 (10), 281 (4), 280 (6), 271 (12), 270 (M^+, 60), 269 (94), 268 (100), 267 (6), 266 (21), 263 (4), 261 (4), 199 (4), 198 (4), 197 (4), 177 (6), 171 (4), 164 (4), 163 (10), 162 (18), 161 (6), 159 (4), 158 (4), 157 (4), 149 (4), 108 (4), 104 (6). HRMS (ESI) m/z: $[M+H]^+$ Calcd for $C_{18}H_{27}N_2$ 271.2174; Found 271.2166.

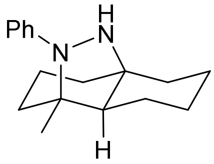

(1*S*,4a*R*,8a*R*)-1-methyl-10-phenyloctahydro-2*H*-1,4a-epidiazano-naphthalene (2k)

Following the *General Procedure I*, **2k** (26.70 mg, 0.10 mmol) was isolated after 20 h at 60ºC by flash chromatography (petroleum ether/EtOAc 19:1) in 69% yield as a yellow oil starting from **1d** (25.00 mg, 0.15 mmol), phenylhydrazine (18 μL, 0.18 mmol), (*S*)-**3g** (7.50 mg, 0.008 mmol) and toluene (1.5 mL, 0.1 *M*). ^{1}H-NMR (δ, ppm) (300 MHz, $CDCl_3$): 7.18 (d, *J* = 5.1 Hz, 4H), 6.70 (dq, *J* = 5.6, 3.0 Hz, 1H), 2.21 (t, *J* = 9.6 Hz, 1H), 1.82-1.69 (m, 3H), 1.66 (dd, *J* = 11.4, 5.1 Hz, 2H), 1.56 (s, 3H), 1.52-1.45 (m, 2H), 1.44-1.32 (m, 5H), 1.29-1.12 (m, 1H). ^{13}C-NMR (δ, ppm) (75.5 MHz, $CDCl_3$): 149.8, 128.4, 121.7, 117.0, 114.8, 67.0, 57.8, 56.4, 40.4, 35.1, 31.1, 24.7, 23.6, 23.0, 21.7, 19.4. The enantiomeric excess (e.e.) was determined by HPLC after derivatization of the corresponding product following the *General Procedure II*. [α]$_D^{20}$: -8.1 (*c* 0.1, CH_2Cl_2). IR (ATR) cm^{-1}: 2928, 1595, 1219, 767. MS (EI, 70 eV) m/z (%): 257 (7), 256 (M^+, 33), 241 (11), 227 (5), 214 (17), 213 (100), 212 (5), 186 (6), 150 (5), 149 (38), 148 (4), 133 (5), 130 (4), 121 (4), 119 (5), 118 (6), 117 (5), 114 (4), 108 (8), 107 (18), 106 (6), 105 (14), 95 (5), 93 (20), 92 (8), 91 (27), 81 (23), 80 (4), 79 (22), 78 (9), 77 (66), 69 (4), 67 (12), 65 (10), 55 (9), 53 (8), 51 (17). HRMS (ESI) m/z: $[M+H]^+$ Calcd for $C_{17}H_{25}N_2$ 257.2018; Found 257.2025.

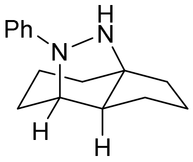

(3a*R*,7*S*,7a*S*)-8-phenyloctahydro-3a,7-epidiazanoindene (2l)

Following the *General Procedure I*, **2l** (26.10 mg, 0.11 mmol) was isolated after 72 h at 50ºC by flash chromatography (petroleum ether/EtOAc 19:1) in 88% yield as a colourless oil starting from **1e** (18.00 mg, 0.13 mmol), phenylhydrazine (18 µL, 0.18 mmol), (*S*)-**3g** (7.500 mg, 0.008 mmol) and toluene (1.5 mL, 0.1 *M*). ^{1}H-NMR (δ, ppm) (300 MHz, $CDCl_3$): 7.20 (dd, *J* = 8.5, 7.2 Hz, 2H), 6.91 (d, *J* = 8.0 Hz, 2H), 6.74-6.63 (m, 1H), 4.22 (d, *J* = 4.3 Hz, 1H), 3.65 (br s, 1H), 2.22-2.12 (m, 1H), 2.04-1.79 (m, 6H), 1.73-1.56 (m, 2H), 1.49-1.20 (m, 5H).^{13}C-NMR (δ, ppm) (75.5 MHz, $CDCl_3$): 149.2, 129.1, 128.7, 116.5, 113.4, 70.1, 60.3, 55.6, 36.4, 33.4, 27.9, 26.6, 22.6, 18.4. The enantiomeric excess (e.e.) was determined by HPLC after derivatization of the corresponding product following the *General Procedure II*. $[\alpha]_D^{20}$: +62.0 (*c* 0.3, CH_2Cl_2). IR (ATR) cm^{-1}: 2936, 1595, 1219, 774. MS (EI, 70 eV) m/z (%): 243 (9), 242 (13), 240 (5), 229 (12), 228 (M^+, 73), 227 (100), 226 (52), 225 (8), 223 (15), 222 (4), 213 (5), 211 (4), 150 (4), 143 (4), 142 (4), 133 (4), 121 (5), 120 (20), 119 (4), 118 (5), 93 (4). HRMS (ESI) m/z: $[M+H]^+$ Calcd for $C_{15}H_{21}N_2$ 229.1705; Found 229.1711.

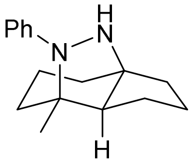

(3a*R*,7*S*,7a*R*)-7-methyl-8-phenyloctahydro-3a,7-epidiazanoindene (2m)

Following the *General Procedure I*, **2m** (33.60 mg, 0.14 mmol) was isolated after 24 h at 60ºC by flash chromatography (petroleum ether/EtOAc 19:1) in 92% yield as a yellow oil starting from **1f** (23.00 mg, 0.15 mmol), phenylhydrazine (18 µL, 0.18 mmol), (*S*)-**3g** (7.50 mg, 0.008 mmol) and toluene (1.5 mL, 0.1 *M*). ^{1}H-NMR (δ, ppm) (300 MHz, $CDCl_3$): 7.22-7.11 (m, 4H), 6.71 (tt, *J* = 6.8, 1.6 Hz, 1H), 4.04 (br s, 1H), 2.24 (ddd, *J* = 8.3, 3.9, 1.5 Hz, 1H), 1.94-1.74 (m, 8H), 1.64 (s, 3H), 1.45-1.35 (m, 4H). ^{13}C-NMR (δ, ppm) (75.5 MHz, $CDCl_3$): 129.1, 128.4, 117.4, 114.9, 77.2, 66.9, 63.3, 35.8, 34.3, 33.4, 25.2, 23.8, 22.3, 19.6. The enantiomeric excess (e.e.) was determined by HPLC after derivatization of the corresponding product following the *General Procedure II*. $[\alpha]_D^{20}$: -14.4 (*c* 0.5, CH_2Cl_2). IR (ATR) cm^{-1}: 2934, 1595, 1219, 767. MS (EI, 70 eV) m/z (%): 242 (M^+, 8), 241 (61), 240 (6), 239 (36), 220 (8), 210 (8), 181 (7), 176 (5), 172 (8), 167 (5), 146 (7), 145 (7), 139 (5), 136 (5), 135 (100), 134 (40), 132 (10), 132 (8), 131 (6), 107 (5), 104 (6), 94 (6). HRMS (ESI) m/z: $[M+H]^+$ Calcd for $C_{16}H_{23}N_2$ 243.1861; Found 243.1865.

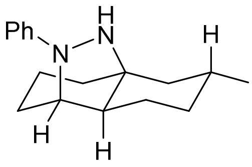

(1*S*,4a*S*,6*R*,8a*S*)-6-methyl-10-phenyloctahydro-2*H*-1,4a-epidiazano-naphthalene (2n).

Following the *General Procedure I*, **2n** (35.80 mg, 0.14 mmol) was isolated after 16 h at 50ºC by flash chromatography (petroleum ether/EtOAc 19:1) in 93% yield as a crystalline white solid starting from **1g** (24.90 mg, 0.15 mmol)), phenylhydrazine (18 µL, 0.18 mmol), (*S*)-**3g** (7.50 mg, 0.008 mmol) and toluene (1.5 mL, 0.1 *M*). ^{1}H-NMR (δ, ppm) (300 MHz, $CDCl_3$): 7.19 (dd, *J* = 8.5, 7.1 Hz, 2H), 6.90 (d, *J* = 8.0 Hz, 2H), 6.66 (td, *J* = 7.2, 1.2 Hz, 1H), 4.04 (br s, 1H), 3.91 (d, *J* = 4.4 Hz, 1H), 2.23-2.09 (m, 1H), 1.92-1.52 (m, 7H), 1.46-1.25 (m, 5H), 1.06 (dd, *J* = 14.3, 12.3 Hz, 1H), 0.93 (d, *J* = 6.3 Hz, 3H). ^{13}C-NMR (δ, ppm) (75.5 MHz, $CDCl_3$): 149.2, 128.7, 116.2, 113.1, 61.9, 60.4, 49.5, 40.5, 40.0, 33.3, 28.1, 28.0, 26.0, 22.4, 18.7. $[\alpha]_D^{20}$: +32.3 (*c* 0.8, CH_2Cl_2). IR (ATR) cm^{-1}: 2924, 1596, 1494, 1275, 1260, 763, 749. MS (EI, 70 eV) m/z (%): 256 (M^+, 6), 255 (5), 253 (8), 252 (7), 242 (4), 241 (38), 240 (100), 239 (52), 238 (4), 237 (5), 236

(8), 235 (5), 231 (6), 223 (4), 214 (6), 213 (10), 212 (9), 211 (9), 209 (4), 181 (4), 148 (4), 106 (4). HRMS (ESI) m/z: $[M+H]^+$ Calcd for $C_{17}H_{25}N_2$ 257.2018; Found 257.2014.

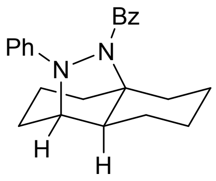

Phenyl((1*S*,4a*R*,8a*S*)-10-phenyloctahydro-2*H*-1,4a-epidiazano-naphthalen-9-yl)methanone (3a)

Following the *General Procedure II*, **3a** (42.00 mg, 0.12 mmol) was isolated after 16 h at room temperature by flash chromatography (petroleum ether/EtOAc 6:4) in 81% yield as a colourless oil starting from **2a** (36.10 mg, 0.15 mmol), triethylamine (25 µL, 0.18 mmol), benzoyl chloride (14 µL, 0.12 mmol) and CH_2Cl_2 (0.20 mL). ^{1}H-NMR (δ, ppm) (300 MHz, $CDCl_3$): 8.17-8.09 (m, 2H), 7.90 (dd, *J* = 7.9, 1.8 Hz, 1H), 7.69-7.57 (m, 1H), 7.52-7.44 (m, 2H), 7.18-7.04 (m, 2H), 6.92-6.77 (m, 2H), 6.62 (t, *J* = 7.1 Hz, 1H), 3.82 (d, *J* = 3.8 Hz, 1H), 3.37 (d, *J* = 11.0 Hz, 1H), 2.67 (dd, *J* = 13.6, 6.1 Hz, 1H), 2.36-2.19 (m, 1H), 2.08 (dt, *J* = 12.0, 6.2 Hz, 1H), 1.92-1.78 (m, 3H), 1.62 (m, 3H), 1.52-1.41 (m, 2H), 1.26 (m, 2H), 1.12 (m, 1H). ^{13}C-NMR (δ, ppm) (75.5 MHz, $CDCl_3$): 172.0, 151.5, 137.1, 133.7, 130.2, 129.7, 129.4, 128.4, 128.3, 127.1, 119.9, 68.9, 68.0, 51.3, 34.7, 32.6, 25.6, 24.8, 22.3, 21.6, 19.6. The enantiomeric excess (e.e.) was determined by HPLC using a CHIRALPAK® AD-3 column (hexane/*i*-PrOH 90:10, 1 mL/min, 210.5 nm, 25ºC); t_r (major) = 11.11 min, t_r (minor) = 12.98 min (96% ee). $[\alpha]_D^{20}$: -3.9 (*c* 0.6, CH_2Cl_2). IR (ATR) cm^{-1}: 1687, 1219, 775. MS (EI, 70 eV) m/z (%): 348 (4), 347 (31), 346 (M^+, 100), 241 (4). HRMS (ESI) m/z: $[M+H]^+$ Calcd for $C_{23}H_{27}N_2O$ 347.2123; Found 347.2130.

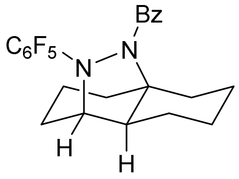

((1*S*,4a*R*,8a*S*)-10-(perfluorophenyl)octahydro-2*H*-1,4a-epidiazano-naphthalen-9-yl)(phenyl)methanone (3b)

Following the *General Procedure II*, **3b** (41.90 mg, 0.09 mmol) was isolated after 16 h at room temperature by flash chromatography (petroleum ether/EtOAc 6:4) in 64% yield as a white foam starting from **2b** (48.00 mg, 0.14 mmol), triethylamine (23 µL, 0.17 mmol), benzoyl chloride (13 µL, 0.11 mmol) and CH_2Cl_2 (0.20 mL). ^{1}H-NMR (δ, ppm) (300 MHz, toluene-d_8): 7.66 (d, *J* = 6.9 Hz, 1H), 7.38 (br s, 1H), 6.83-6.70 (m, 3H), 3.58-3.45 (m, 1H), 2.59 (dd, *J* = 13.6, 5.9 Hz, 1H), 2.01-1.88 (m, 3H), 1.61-1.39 (m, 3H), 1.30 (dd, *J* = 11.4, 5.8 Hz, 2H), 1.17 (dt, *J* = 8.1, 4.2 Hz, 2H), 1.09-0.96 (m, 4H). ^{13}C-NMR (δ, ppm) (75.5 MHz, toluene-d_8): 168.6, 137.0, 129.3, 129.1, 127.7, 127.6, 127.0, 126.9, 68.7, 68.0, 50.7, 39.2, 34.5, 32.0, 30.8, 30.2, 25.6, 24.5. ^{19}F-NMR (δ, ppm) (282 MHz, toluene-d_8): -149.79 (d, *J* = 21.1 Hz), -150.35 (d, *J* = 22.1 Hz), -150.52 (d, *J* = 22.9 Hz), -164.00 (t, *J* = 21.3 Hz), -164.59 (td, *J* = 23.0, 5.3 Hz), -166.84 (t, *J* = 22.1 Hz), -167.82 (t, *J* = 22.2 Hz).. The enantiomeric excess (e.e.) was determined by HPLC using a CHIRALPAK® IA column (hexane/*i*-PrOH 90:10, 1 mL/min, 250 nm, 25ºC); t_r (major) = 7.87 min, t_r (minor) = 7.21 min (90% ee). $[\alpha]_D^{20}$: +84.7 (*c* 2.1, CH_2Cl_2). IR (ATR) cm^{-1}: 1638, 1515, 1497, 1048, 982, 764, 749. MS (EI, 70 eV) m/z (%): 438 (4), 437 (26), 436 (M^+, 100), 331 (2). HRMS (ESI) m/z: $[M+H]^+$ for $C_{23}H_{22}N_2OF_5$ 437.1652; Found 437.1656. M.p.: >200 ºC (EtOAc/hexane).

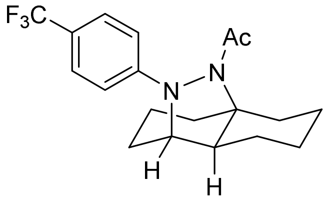

1-((1*S*,4a*R*,8a*S*)-10-(4-(trifluoromethyl)phenyl)octahydro-2*H*-1,4a-epidiazanonaphthalen-9-yl)ethan-1-one (3c)

Following the *General Procedure III*, **3c** (27.40 mg, 0.08 mmol) was isolated after 2 h at room temperature by flash chromatography (petroleum ether/EtOAc 1:1) in 80% yield as a colourless oil starting from **2c** (31.30 mg, 0.10 mmol), DMAP (2.40 mg, 0.02 mmol), pyridine (81 µL, 1 mmol), acetyl chloride (71 µL, 1 mmol) and CH_2Cl_2 (0.83 mL). ^{1}H-NMR (δ, ppm) (300 MHz, $CDCl_3$): 7.53-7.45 (m, 2H), 7.17 (dd, *J* = 9.0, 2.5 Hz, 1H), 6.83 (dd, *J* = 8.4, 2.5 Hz, 1H), 3.79 (d, *J* = 2.3 Hz, 1H), 3.07 (dd, *J* = 11.5, 4.4 Hz, 1H), 2.50 (dd, *J* = 13.1, 5.1 Hz, 1H), 2.05 (s, 3H), 1.86-1.21 (m, 11H), 1.14-0.99 (m, 1H), 0.97-0.85 (m, 1H). ^{13}C-NMR (δ, ppm) (75.5 MHz, $CDCl_3$): 176.7, 155.3, 126.4, 122.2 (q, *J* = 31.8 Hz), 116.4, 114.0, 68.5, 64.3, 51.8, 34.4, 32.3, 31.9, 25.7, 25.3, 23.9, 22.2, 19.9. ^{19}F-NMR (δ, ppm) (282 MHz, $CDCl_3$): -61,48. The enantiomeric excess (e.e.) was determined by HPLC using a CHIRALPAK® IA column (hexane/*i*-PrOH 99:01, 1 mL/min, 250 nm, 30ºC); t_r (major) = 13.58 min, t_r (minor) = 12.75 min (83% ee). $[\alpha]_D^{20}$: +60.5 (*c* 1.2, CH_2Cl_2). IR (ATR) cm^{-1}: 1666, 1611, 1321, 763, 750. MS (EI, 70 eV) m/z (%): 354 (3), 353 (23), 352 (M^+, 100), 350 (1), 310 (1), 309 (3). HRMS (ESI) m/z: $[M+H]^+$ Calcd for $C_{19}H_{24}N_2OF_3$ 353.1841; Found 353.1845.

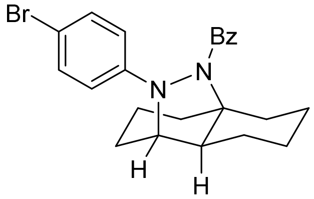

((1*S*,4a*R*,8a*S*)-10-(4-bromophenyl)octahydro-2*H*-1,4a-epidiazano-naphthalen-9-yl)(phenyl)methanone (3d)

Following the *General Procedure II*, **3d** (27.60 mg, 0.07 mmol) was isolated after 16 h at room temperature by flash chromatography (petroleum ether/EtOAc 7:3) in 54% yield as a colourless oil starting from **2d** (40.60 mg, 0.13 mmol), triethylamine (25 µL, 0.18 mmol), benzoyl chloride (14 µL, 0.12 mmol) and CH_2Cl_2 (0.20 mL). ^{1}H-NMR (δ, ppm) (300 MHz, $CDCl_3$): 7.95-7.80 (m, 2H), 7.45 (dd, *J* = 13.2, 7.1 Hz, 2H), 7.31 (d, *J* = 8.7 Hz, 1H), 7.22-7.08 (m, 3H), 6.72-6.53 (m, 2H), 3.76 (d, *J* = 2.7 Hz, 1H), 3.33 (d, *J* = 11.2 Hz, 1H), 2.65 (dd, *J* = 13.6, 6.0 Hz, 1H), 2.24 (d, *J* = 9.2 Hz, 1H), 1.91-1.75 (m, 3H), 1.71-1.36 (m, 6H), 1.17-0.98 (m, 3H). ^{13}C-NMR (δ, ppm) (75.5 MHz, $CDCl_3$): 171.1, 150.8, 136.9, 131.3, 129.9, 128.3, 127.3, 112.0, 68.9, 68.2, 51.3, 39.6, 34.5, 32.5, 25.7, 24.7, 22.3, 19.5. The enantiomeric excess (e.e.) was determined by HPLC using a CHIRALPAK® IA column (hexane/*i*-PrOH 90:10, 1 mL/min, 250 nm, 25ºC); t_r (major) = 12.99 min, t_r (minor) = 19.15 min (96% ee). $[\alpha]_D^{20}$: -1.4 (*c* 2.0, CH_2Cl_2). IR (ATR) cm^{-1}: 1637, 1260, 1275, 763, 750. MS (EI, 70 eV) m/z (%): 427 (26), 426 (94), 425 (26), 424 (M^+, 100). HRMS (ESI) m/z: $[M+H]^+$ Calcd for $C_{23}H_{26}N_2OBr$ 425.1229; Found 425.1222.

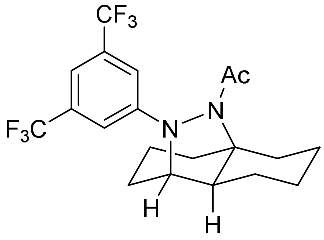

1-((1*S*,4a*R*,8a*S*)-10-(3,5-bis(trifluoromethyl)phenyl)octahydro-2*H*-1,4a-epidiazanonaphthalen-9-yl)ethan-1-one (3e)

Following the *General Procedure III*, **3e** (27.70 mg, 0.07 mmol) was isolated after 3 h at room temperature by flash chromatography (petroleum ether/EtOAc 1:1) in 88% yield as a pale yellow oil starting from **2e** (29.60 mg, 0.08 mmol), DMAP (1.90 mg, 0.02 mmol), pyridine (65 μL, 0.80 mmol), acetyl chloride (57 μL, 0.80 mmol) and CH_2Cl_2 (0.66 mL). ^{1}H-NMR (δ, ppm) (300 MHz, $CDCl_3$): 7.51 (s, 1H), 7.36 (s, 1H), 7.15 (s, 1H), 3.79 (d, *J* = 3.2 Hz, 1H), 3.07 (dd, *J* = 12.2, 4.9 Hz, 1H), 2.53 (dd, *J* = 12.9, 5.0 Hz, 1H), 2.04 (s, 3H), 1.91-1.53 (m, 6H), 1.50-1.24 (m, 5H), 1.17-1.00 (m, 1H), 0.88-0.70 (m, 1H). ^{13}C-NMR (δ, ppm) (75.5 MHz, $CDCl_3$): 176.5, 154.0, 132.5 (qd, *J* = 33.4, 3.7 Hz), 125.0, 121.4, 116.4, 113.9 (m), 69.1, 68.8, 51.8, 34.3, 32.2, 31.7, 25.9, 24.5, 23.8, 22.2, 19.8. ^{19}F-NMR (δ, ppm) (282 MHz, $CDCl_3$): -62.99, -63.18. The enantiomeric excess (e.e.) was determined by HPLC using a CHIRALPAK® IC column (hexane/*i*-PrOH 95:05, 1 mL/min, 250 nm, 25ºC); t_r (major) = 6.73 min, t_r (minor) = 7.29 min (72% ee). $[\alpha]_D^{20}$: -23.4 (*c* 2.0, CH_2Cl_2). IR (ATR) cm^{-1}: 1671, 1275, 1260, 763, 750. MS (EI, 70 eV) m/z (%): 422 (3), 421 (23), 420 (M^+, 100), 418 (1), 378 (2), 377 (6), 376 (2). HRMS (ESI) m/z: $[M+H]^+$ Calcd for $C_{20}H_{23}N_2OF_6$ 421.1715; Found 421.1715.

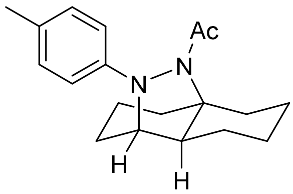

1-((1*S*,4a*R*,8a*S*)-10-(*p*-tolyl)octahydro-2*H*-1,4a-epidiazano-naphthalen-9-yl)ethan-1-one (3f)

Following the *General Procedure III*, **3f** (36.80 mg, 0.12 mmol) was isolated after 3 h at room temperature by flash chromatography (petroleum ether/EtOAc 1:1) in 87% yield as a colourless oil starting from **2f** (38.50 mg, 0.15 mmol), DMAP (3.60 mg, 0.03 mmol), pyridine (122 μL, 1.5 mmol), acetyl chloride (107 μL, 1.5 mmol) and CH_2Cl_2 (1.25 mL). ^{1}H-NMR (δ, ppm) (300 MHz, $CDCl_3$): 7.07-7.04 (m, 3H), 6.69-6.67 (m, 1H), 3.70 (d, *J* = 4.0 Hz, 1H), 3.12 (dd, *J* = 13.5, 5.0 Hz, 1H), 2.49 (dd, *J* = 13.1, 5.4 Hz, 1H), 2.27 (s, 3H), 2.06 (s, 3H), 1.84-1.38 (m, 9H), 1.38-1.01 (m, 4H). ^{13}C-NMR (δ, ppm) (75.5 MHz, $CDCl_3$): 176.8, 150.3, 129.6, 129.2, 116.8, 114.5, 68.3, 68.2, 52.0, 34.7, 32.5, 32.4, 25.6, 24.8, 24.1, 22.2, 20.4, 20.0. The enantiomeric excess (e.e.) was determined by HPLC using a CHIRALPAK® IC column (hexane/*i*-PrOH 90:10, 1 mL/min, 254.8 nm, 25ºC); t_r (major) = 14.02 min, t_r (minor) = 12.81 min (94% ee). $[\alpha]_D^{20}$: +47.4 (*c* 2.6, CH_2Cl_2). IR (ATR) cm^{-1}: 1661, 1275, 1260, 763, 750. MS (EI, 70 eV) m/z (%): 312 (1), 300 (4), 299 (25), 298 (M^+, 100), 297 (4), 296 (1), 256 (1), 255 (4), 254 (3), 240 (1), 134 (1). HRMS (ESI) m/z: $[M+H]^+$ Calcd for $C_{19}H_{27}N_2O$ 299.2123; Found 299.2130.

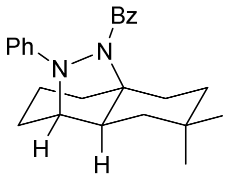

((1*S*,4a*S*,8a*S*)-7,7-dimethyl-10-phenyloctahydro-2*H*-1,4a-epidiazano-naphthalen-9-yl)(phenyl)methanone (3i)

Following the *General Procedure II*, **3i** (55.40 mg, 0.14 mmol) was isolated after 16 h at room temperature by flash chromatography (petroleum ether/EtOAc 7:3) in 99% yield as a colourless

oil starting from **2i** (40.40 mg, 0.15 mmol), triethylamine (29 µL, 0.21 mmol), benzoyl chloride (16 µL, 0.14 mmol) and CH_2Cl_2 (0.23 mL). ^{1}H-NMR (δ, ppm) (300 MHz, $CDCl_3$): 7.88 (dd, *J* = 7.8, 1.9 Hz, 2H), 7.59-7.48 (m, 1H), 7.42 (d, *J* = 7.4 Hz, 1H), 7.23-7.17 (m, 1H), 7.14-7.05 (m, 3H), 6.82 (t, *J* = 7.2 Hz, 1H), 6.69-6.56 (m, 1H), 3.83-3.74 (m, 1H), 3.27 (dt, *J* = 14.7, 3.4 Hz, 1H), 2.70 (dd, *J* = 13.5, 6.1 Hz, 1H), 2.31-2.20 (m, 1H), 1.99 (dd, *J* = 12.4, 6.1 Hz, 2H), 1.87-1.70 (m, 2H), 1.60-1.51 (m, 4H), 1.30 (ddd, *J* = 16.1, 6.1, 2.8 Hz, 2H), 0.86 (s, 3H), 0.71 (s, 3H). ^{13}C-NMR (δ, ppm) (75.5 MHz, $CDCl_3$): 162.3, 137.2, 134.5, 130.5, 129.6, 128.8, 128.3, 127.1, 120.0, 68.2, 68.1, 48.4, 39.4, 37.7, 34.9, 32.8, 32.2, 29.8, 28.2, 23.5, 19.7. The enantiomeric excess (e.e.) was determined by HPLC using a CHIRALPAK® IA column (hexane/*i*-PrOH 95:05, 1 mL/min, 250 nm, 25ºC); t_r (major) = 11.29 min, t_r (minor) = 13.65 min (98% ee). $[\alpha]_D^{20}$: -6.6 (*c* 2.1, CH_2Cl_2). IR (ATR) cm^{-1}: 1698, 1219, 769. MS (EI, 70 eV) m/z (%): 376 (5), 375 (32), 374 (M^+, 100), 269 (2). HRMS (ESI) m/z: $[M+H]^+$ Calcd for $C_{25}H_{31}N_2O$ 375.2436; Found 375.2443.

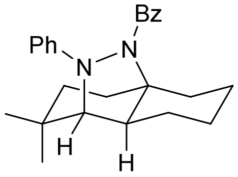

((1*R*,4a*R*,8a*S*)-2,2-dimethyl-10-phenyloctahydro-2*H*-1,4a-epidiazano-naphthalen-9-yl)(phenyl)methanone (3j)

Following the *General Procedure II*, **3j** (51.30 mg, 0.14 mmol) was isolated after 16 h at room temperature by flash chromatography (petroleum ether/EtOAc 7:3) in 98% yield as a colourless oil starting from **2j** (38.10 mg, 0.14 mmol), triethylamine (28 µL, 0.20 mmol), benzoyl chloride (15 µL, 0.13 mmol) and CH_2Cl_2 (0.22 mL). ^{1}H-NMR (δ, ppm) (300 MHz, $CDCl_3$): 8.19 (dd, *J* = 8.4, 1.4 Hz, 1H), 7.87 (dd, *J* = 7.9, 1.8 Hz, 2H), 7.60-7.51 (m, 1H), 7.42 (dt, *J* = 5.5, 1.6 Hz, 1H), 7.12 (qt, *J* = 7.1, 1.2 Hz, 2H), 6.99-6.82 (m, 2H), 6.70-6.59 (m, 1H), 3.45-3.38 (m, 1H), 3.26 (d, *J* = 16.0 Hz, 1H), 2.64-2.50 (m, 1H), 2.15 (t, *J* = 8.7 Hz, 1H), 1.94-1.76 (m, 1H), 1.74-1.45 (m, 8H), 1.38 (s, 3H), 1.28-1.17 (m, 1H) 1.11 (s, 3H). ^{13}C-NMR (δ, ppm) (75.5 MHz, $CDCl_3$) (presence of rotatory isomers): 171.1, 152.1, 137.3, 134.5, 130.5, 129.5, 128.0, 127.1, 120.0, 78.7, 68.6, 46.2, 36.0, 33.1, 32.3, 32.1, 29.7, 25.8, 25.5, 25.0, 22.4. The enantiomeric excess (e.e.) was determined by HPLC using a CHIRALPAK® IA column (hexane/*i*-PrOH 90:10, 1 mL/min, 250 nm, 25ºC); t_r (major) = 8.90 min, t_r (minor) = 7.52 min (98% ee). $[\alpha]_D^{20}$: +11.6 (*c* 1.00, CH_2Cl_2). IR (ATR) cm^{-1}: 1683, 1219, 771. MS (EI, 70 eV) m/z (%): 376 (4), 375 (30), 374 (M^+, 100), 372 (1), 269 (3). HRMS (ESI) m/z: $[M+H]^+$ Calcd for $C_{25}H_{31}N_2O$ 375.2436; Found 375.2444.

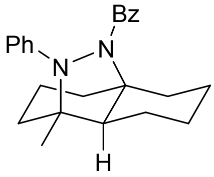

((1*S*,4a*R*,8a*S*)-1-methyl-10-phenyloctahydro-2*H*-1,4a-epidiazano-naphthalen-9-yl)(phenyl)methanone (3k)

Following the *General Procedure II*, **3k** (24.70 mg, 0.07 mmol) was isolated after 16 h at room temperature by flash chromatography (petroleum ether/EtOAc 6:4) in 69% yield as a colourless oil starting from **2k** (26.70 mg, 0.10 mmol), triethylamine (19 µL, 0.14 mmol), benzoyl chloride (11 µL, 0.09 mmol) and CH_2Cl_2 (0.15 mL). ^{1}H-NMR (δ, ppm) (300 MHz, $CDCl_3$): 7.66 (dd, *J* = 7.6, 2.1 Hz, 2H), 7.48-7.33 (m, 1H), 7.22-7.13 (m, 2H), 7.03 (d, *J* = 6.4 Hz, 2H), 6.98-6.84 (m, 2H), 6.66 (t, *J* = 7.3 Hz, 1H), 3.50 (dd, *J* = 13.8, 2.5 Hz, 1H), 2.63 (dd, *J* = 13.8, 6.2 Hz, 1H), 2.20-1.90 (m, 3H), 1.85-1.66 (m, 7H), 1.32-1.17 (m, 3H), 1.15 (s, 3H). ^{13}C-NMR (δ, ppm) (75.5 MHz, $CDCl_3$) (presence of rotatory isomers): 168.9, 148.6, 137.6, 130.4, 128.9, 128.1, 126.9, 121.6, 119.5, 66.2, 64.6, 55.0, 40.0, 35.4, 32.9, 24.9, 23.1, 22.3, 21.4, 19.6. The enantiomeric excess (e.e.) was determined by HPLC using a CHIRALPAK® IA column (hexane/*i*-PrOH 90:10, 1 mL/min, 250 nm, 25ºC); t_r (major) = 13.18 min, t_r (minor) = 10.42 min (80% ee). $[\alpha]_D^{20}$: -9.1 (*c*

0.5, CH_2Cl_2). IR (ATR) cm^{-1}: 1686, 1219, 771. MS (EI, 70 eV) m/z (%): 362 (5), 361 (29), 360 (M^+, 100), 358 (1), 255 (3), 254 (1), 148 (1). HRMS (ESI) m/z: $[M+H]^+$ Calcd for $C_{24}H_{29}N_2O$ 361.2280; Found 361.2287.

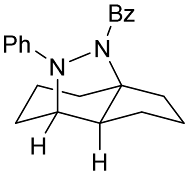

Phenyl((3a*R*,7*S*,7a*S*)-8-phenyloctahydro-3a,7-epidiazanoinden-9-yl)methanone (3l)

Following the *General Procedure II*, **3l** (16.60 mg, 0.05 mmol) was isolated after 16 h at room temperature by flash chromatography (petroleum ether/EtOAc 7:3) in 70% yield as a colourless oil starting from **2l** (17.80 mg, 0.07 mmol), triethylamine (13 μL, 0.09 mmol), benzoyl chloride (7 μL, 0.06 mmol) and CH_2Cl_2 (0.10 mL). ^1H-NMR (δ, ppm) (300 MHz, $CDCl_3$): 7.94 (dd, *J* = 7.8, 1.9 Hz, 2H), 7.16-7.06 (m, 3H), 6.99 (td, *J* = 7.1, 2.4 Hz, 2H), 6.76 (d, *J* = 8.1 Hz, 2H), 6.66 (dd, *J* = 7.9, 6.7 Hz, 1H), 4.18 (dd, *J* = 3.9, 1.9 Hz, 1H), 3.16-3.01 (m, 1H), 2.95 (dd, *J* = 13.3, 6.1 Hz, 1H), 2.27 (dt, *J* = 13.0, 5.1 Hz, 1H), 2.13-2.01 (m, 1H), 2.01-1.46 (m, 9H). ^{13}C-NMR (δ, ppm) (75.5 MHz, $CDCl_3$): 168.8, 150.9, 136.5, 129.8, 128.3, 127.2, 120.4, 115.0, 66.7, 56.7, 36.2, 34.8, 32.3, 31.7, 26.3, 22.8, 19.7. The enantiomeric excess (e.e.) was determined by HPLC using a CHIRALPAK® AD-H column (hexane/*i*-PrOH 90:10, 1 mL/min, 250 nm, 25ºC); t_r (major) = 11.16 min (>99% ee). $[\alpha]_D^{20}$: +22.9 (*c* 1.3, CH_2Cl_2). IR (ATR) cm^{-1}: 1624, 1218, 771. MS (EI, 70 eV) m/z (%): 334 (4), 333 (25), 332 (M^+, 100), 227 (2), 105 (1). HRMS (ESI) m/z: $[M+H]^+$ Calcd for $C_{22}H_{25}N_2O$ 333.1967; Found 333.1977.

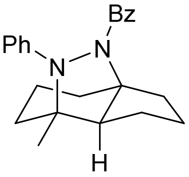

((3a*R*,7*S*,7a*S*)-7-methyl-8-phenyloctahydro-3a,7-epidiazanoinden-9-yl)(phenyl)methanone (3m)

Following the *General Procedure II*, **3m** (27.80 mg, 0.08 mmol) was isolated after 16 h at room temperature by flash chromatography (petroleum ether/EtOAc 7:3) in 57% yield as a colourless oil starting from **2m** (33.60 mg, 0.14 mmol), triethylamine (28 μL, 0.20 mmol), benzoyl chloride (15 μL, 0.13 mmol) and CH_2Cl_2 (0.22 mL). ^1H-NMR (δ, ppm) (300 MHz, $CDCl_3$): 7.73-7.67 (m, 2H), 7.65-7.54 (m, 1H), 7.50-7.38 (m, 1H), 7.23-7.12 (m, 1H), 7.09-7.00 (m, 2H), 6.98-6.89 (m, 2H), 6.78-6.62 (m, 1H), 3.31-3.10 (m, 1H), 2.91 (dd, *J* = 13.3, 6.1 Hz, 1H), 2.13-1.93 (m, 5H), 1.94-1.67 (m, 5H), 1.47-1.38 (m, 1H), 1.20 (s, 3H). ^{13}C-NMR (δ, ppm) (75.5 MHz, $CDCl_3$): 167.9, 148.4, 136.9, 130.6, 129.1, 128.1, 126.9, 122.1, 118.9, 76.7, 66.7, 62.0, 41.0, 34.9, 32.1, 25.0, 23.1, 22.1, 20.4 (**C**-5). The enantiomeric excess (e.e.) was determined by HPLC using a CHIRALPAK® AD-H column (hexane/*i*-PrOH 90:10, 1 mL/min, 250 nm, 25ºC); t_r (major) = 12.34 min, t_r (minor) = 9.86 min (74% ee). $[\alpha]_D^{20}$: +4.3 (*c* 1.0, CH_2Cl_2). IR (ATR) cm^{-1}: 1683, 1219, 766. MS (EI, 70 eV) m/z (%): 348 (4), 347 (30), 346 (M^+, 100), 344 (1), 241 (2), 135 (2), 134 (1), 105 (1). HRMS (ESI) m/z: $[M+H]^+$ Calcd for $C_{23}H_{27}N_2O$ 347.2123; Found 347.2129.

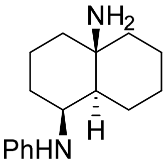

(1*S*,4a*R*,8a*S*)-*N*¹-phenyloctahydronaphthalene-1,4a(2*H*)-diamine (4a)

Following the *General Procedure*, **4a** (14.90 mg, 0.06 mmol) was isolated after 40 min at 80ºC by flash chromatography (petroleum ether/EtOAc 1:1) in 98% yield as a colourless oil starting from **2a** (15.00 mg, 0.06 mmol). ^1H-NMR (δ, ppm) (300 MHz, $CDCl_3$): 7.11 (dd, *J* = 8.4, 7.2 Hz,

2H), 6.58-6.51 (m, 3H), 3.54 (q, *J* =3.2 Hz, 1H), 2.03 (dtd, *J* = 13.5, 4.9, 4.4, 1.8 Hz, 1H), 1.98-1.83 (m, 1H), 1.76 (ddd, *J* = 14.4, 4.3, 2.2 Hz, 1H), 1.65-1.46 (m, 2H), 1.45-1.18 (m, 10H). ^{13}C-NMR (δ, ppm) (75.5 MHz, $CDCl_3$): 148.6, 129.1, 115.1, 112.4, 52.3, 51.6, 46.0, 43.8, 41.6, 29.8, 26.4, 26.3, 21.5, 16.8. The enantiomeric excess (e.e.) was determined by HPLC using a CHIRALPAK® OD-3 column (hexane/*i*-PrOH 90:10, 1 mL/min, 250 nm, 25ºC); t_r (major) = 4.35 min, t_r (minor) = 5.17 min (92% ee). $[\alpha]_D^{20}$: -59.5 (*c*= 2.5, CH_2Cl_2). IR (ATR) cm^{-1}: 3005, 2989, 2926, 1600, 1508, 1275, 1260, 763, 750. MS (EI, 70 eV) m/z (%): 259 (4), 246 (15), 245 (68), 244 (M^+, 100), 243 (7), 241 (7), 228 (6), 227 (9), 226 (20), 225 (9), 224 (4), 134 (5). HRMS (ESI) m/z: $[M+H]^+$ Calcd for $C_{16}H_{25}N_2$ 245.2018; Found 245.2024.

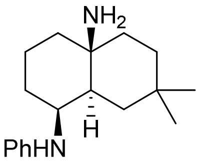

(1*S*,4a*S*,8a*S*)-7,7-dimethyl-*N*^{1}-phenyloctahydronaphthalene-1,4a(2*H*)-diamine (4b)

Following the *General Procedure*, **4b** (22.60 mg, 0.08 mmol) was isolated after 40 min at 80ºC by flash chromatography (petroleum ether/EtOAc 1:1) in 92% yield as a colourless oil starting from **2i** (24.30 mg, 0.09 mmol).^{1}H-NMR (δ, ppm) (300 MHz, $CDCl_3$): 7.06 (t, *J* = 7.7 Hz, 2H), 6.58-6.41 (m, 3H), 3.05-2.93 (br s, 1H), 1.96 (t, *J* = 13.8 Hz, 1H), 1.78-1.16 (m, 12H), 1.07 (s, 3H), 0.93 (s, 3H). ^{13}C-NMR (δ, ppm) (75.5 MHz, $CDCl_3$): 150.7, 129.0, 114.3, 111.6, 62.2, 43.6, 42.1, 37.3, 36.2, 30.3, 29.7, 28.9, 26.3, 26.1, 25.8, 21.5. $[\alpha]_D^{20}$: +228.2 (*c*= 1.5, CH_2Cl_2). IR (ATR) cm^{-1}: 3303, 2924, 2848, 1599, 1508, 1275, 1260, 763, 749. MS (EI, 70 eV) m/z (%): 274 (6), 273 (42), 272 (M^+, 100), 118 (6), 93 (4), 92 (3), 82 (5), 77 (5), 71 (3), 69 (4), 65 (3), 57 (3), 56 (4), 55 (5). HRMS (ESI) m/z: $[M+H]^+$ Calcd for $C_{18}H_{29}N_2$ 273.2331; Found 273.2337.

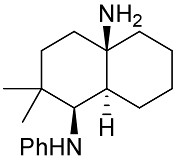

(1*R*,4a*R*,8a*S*)-2,2-dimethyl-*N*^{1}-phenyloctahydronaphthalene-1,4a(2*H*)-diamine (4c)

Following the *General Procedure*, **4c** (24.70 mg, 0.09 mmol) was isolated after 40 min at 80ºC by flash chromatography (petroleum ether/EtOAc 1:1) in 91% yield as a colourless oil starting from **2j** (27.00 mg, 0.1 mmol).^{1}H-NMR (δ, ppm) (300 MHz, $CDCl_3$): 7.18-7.08 (m, 2H), 6.65-6.43 (m, 1H), 3.49 (d, *J* = 2.9 Hz, 1H), 2.04 (ddd, *J* = 14.6, 3.9, 1.7 Hz, 1H), 1.93 (dt, *J* = 14.1, 3.6 Hz, 1H), 1.59 (dd, *J* = 5.2, 2.3 Hz, 2H), 1.54-1.44 (m, 2H), 1.41-1.31 (m, 3H), 1.30-1.22 (m, 2H), 1.14-1.06 (m, 1H), 1.05-1.01 (m, 1H), 0.95 (d, *J* = 2.3 Hz, 6H). ^{13}C-NMR (δ, ppm) (75.5 MHz, $CDCl_3$): 148.6, 129.1, 115.2, 112.5, 52.4, 51.4, 41.3, 40.9, 39.9, 39.3, 34.3, 33.3, 30.9, 29.7, 24.8, 16.9. $[\alpha]_D^{20}$: +138.1 (*c*= 1.1, CH_2Cl_2). IR (ATR) cm^{-1}: 3325, 2924, 2855, 1600, 1518, 1275, 1260, 763, 749. MS (EI, 70 eV) m/z (%): 273 (29), 272 (M^+, 100). HRMS (ESI) m/z: $[M+H]^+$ Calcd for $C_{18}H_{29}N_2$ 273.2331; Found 273.2336.

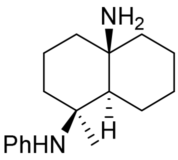

(1*S*,4a*R*,8a*R*)-1-methyl-*N*^{1}-phenyloctahydronaphthalene-1,4a(2*H*)-diamine (4d)

Following the *General Procedure*, **4d** (22.80 mg, 0.09 mmol) was isolated after 40 min at 80ºC by flash chromatography (EtOAc/MeOH 3%) in 88% yield as a colourless oil starting from **2k** (25.60 mg, 0.10 mmol).^{1}H-NMR (δ, ppm) (300 MHz, $CDCl_3$): 7.11 (dd, *J* = 8.5, 7.2 Hz, 2H), 6.78 (dd, *J* = 8.5, 1.2 Hz, 2H), 6.71 (t, *J* = 7.4 Hz, 1H), 2.30-2.20 (m, 1H), 1.90-1.75 (m, 3H), 1.61-

1.54 (m, 3H), 1.42-1.36 (m, 2H), 1.33 (s, 3H), 1.28-1.22 (m, 2H), 1.10 (td, *J* = 13.0, 12.4, 3.1 Hz, 4H).^{13}C-NMR (δ, ppm) (75.5 MHz, $CDCl_3$): 147.5, 128.7, 118.4, 115.7, 56.2, 53.4, 43.1, 41.6, 35.7, 29.7, 28.7, 26.8, 21.3, 20.9, 17.7. $[\alpha]_D^{20}$: +326.7 (*c*= 0.5, CH_2Cl_2). IR (ATR) cm^{-1}: 3270, 2929, 2857, 1597, 1496, 1275, 1260, 763, 750. MS (EI, 70 eV) m/z (%): 260 (14), 259 (75), 258 (M^+, 100), 256 (4), 255 (5), 149 (5), 148 (4). HRMS (ESI) m/z: $[M+H]^+$ Calcd for $C_{17}H_{27}N_2$ 259.2174; Found 259.2176.

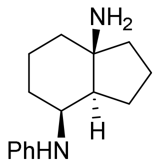

(3a*R*,7*S*,7a*S*)-*N*^{7}-phenyloctahydro-3a*H*-indene-3a,7-diamine (4e)

Following the General Procedure, **4e** (21.30 mg, 0.09 mmol) was isolated after 40 min at 80ºC by flash chromatography (EtOAc/MeOH 3%) in 93% yield as a colourless oil starting from **2l** (22.80 mg, 0.10 mmol).^{1}H-NMR (δ, ppm) (300 MHz, $CDCl_3$): 7.11 (dd, *J* = 8.5, 7.1 Hz, 2H), 6.61 (d, *J* = 8.1 Hz, 3H), 3.84 (d, *J* = 3.1 Hz, 1H), 2.10-1.60 (m, 7H), 1.52-1.30 (m, 6H). ^{13}C-NMR (δ, ppm) (75.5 MHz, $CDCl_3$): 148.7, 129.0, 116.1, 113.2, 60.0, 50.0, 48.2, 37.8, 30.2, 29.7, 24.1, 19.0, 17.2. $[\alpha]_D^{20}$: +463.7 (*c*= 0.4, CH_2Cl_2). IR (ATR) cm^{-1}: 3306, 2929, 2875, 1600, 1508, 1275, 1260, 763, 750. MS (EI, 70 eV) m/z (%): 232 (5), 231 (38), 230 (M^+, 100), 229 (3), 227 (4), 214 (4), 213 (6), 212 (6). HRMS (ESI) m/z: $[M+H]^+$ Calcd for $C_{15}H_{23}N_2$ 231.1861; Found 231.1858.

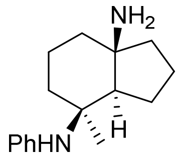

(3a*R*,7*S*,7a*R*)-7-methyl-*N*^{7}-phenyloctahydro-3a*H*-indene-3a,7-diamine (4f)

Following the General Procedure, **4f** (12.90 mg, 0.05 mmol) was isolated after 40 min at 80ºC by flash chromatography (petroleum ether/EtOAc 1:1) in 97% yield as a colourless oil starting from **2m** (13.10 mg, 0.05 mmol). ^{1}H-NMR (δ, ppm) (300 MHz, $CDCl_3$): 7.12 (dd, *J* = 8.6, 7.2 Hz, 2H), 6.77 (d, *J* = 7.4 Hz, 2H), 6.68 (tt, *J* = 7.3, 1.1 Hz, 1H), 2.79 (br s, 2H), 2.35 (d, *J* = 13.1 Hz, 1H), 1.90-1.66 (m, 6H), 1.54-1.44 (m, 2H), 1.42-1.38 (m, 1H), 1.36 (s, 3H), 1.27 (d, *J* = 7.5 Hz, 2H), 1.04 (td, *J* = 13.4, 3.7 Hz, 1H). ^{13}C-NMR (δ, ppm) (75.5 MHz, $CDCl_3$): 147.7, 128.7, 117.6, 117.5, 58.8, 55.7, 55.7, 42.4, 39.2, 35.1, 28.4, 21.1, 19.0, 18.7. $[\alpha]_D^{20}$: +181.0 (*c*= 1.0, CH_2Cl_2). IR (ATR) cm^{-1}: 3270, 2930, 2872, 1598, 1496, 1275, 1260, 763, 750. MS (EI, 70 eV) m/z (%): 246 (10), 245 (74), 244 (M^+, 100), 241 (4). HRMS (ESI) m/z: $[M+H]^+$ Calcd for $C_{16}H_{25}N_2$ 245.2018; Found 245.2022.

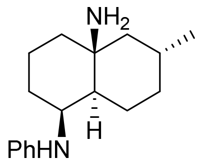

(1*S*,4a*S*,6*R*,8a*S*)-6-methyl-*N*^{1}-phenyloctahydronaphthalene-1,4a(2*H*)-diamine (4g)

Following the General Procedure, **4g** (22.20 mg, 0.09 mmol) was isolated after 40 min at 80ºC by flash chromatography (petroleum ether/EtOAc 1:1) in 90% yield as a colourless oil starting from **2n** (25.00 mg, 0.1 mmol).^{1}H-NMR (δ, ppm) (300 MHz, $CDCl_3$): 7.11 (t, *J* = 7.8 Hz, 2H), 6.64-6.50 (m, 3H,), 3.57 (d, *J* = 3.3 Hz, 1H), 2.12-1.85 (m, 2H), 1.84-1.60 (m, 3H), 1.50 (d, *J* = 13.3 Hz, 1H), 1.33 (tdd, *J* = 16.2, 7.9, 3.3 Hz, 6H), 1.13-0.96 (m, 2H), 0.89 (d, *J* = 6.2 Hz, 3H). ^{13}C-NMR (δ, ppm) (75.5 MHz, $CDCl_3$): 148.7, 129.1, 115.3, 112.6, 52.6, 52.1, 45.6, 41.4, 35.2, 29.9, 29.7, 27.8, 26.3, 22.4, 16.7. $[\alpha]_D^{20}$: +249.9 (*c*= 1.3, CH_2Cl_2). IR (ATR) cm^{-1}: 3305, 2922, 2862, 1599, 1508, 1275, 1260, 763, 749. MS (EI, 70 eV) m/z (%): 260 (5), 259 (33), 258 (M^+, 100), 118 (3), 92 (3), 77 (4), 57 (4), 55 (3). HRMS (ESI) m/z: $[M+H]^+$ Calcd for $C_{17}H_{27}N_2$ 259.2174; Found 259.2173.

3. NMR spectra

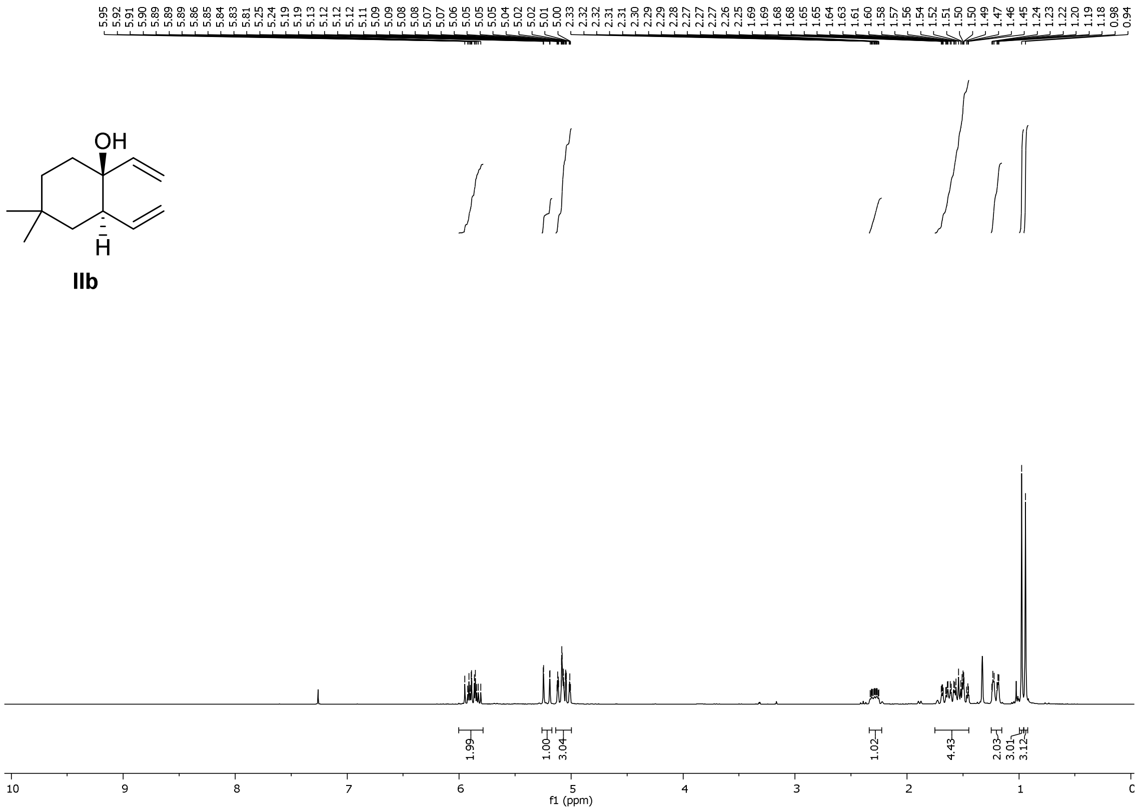

Figure SI-1. ^{1}H-NMR (300 MHz, $CDCl_3$) spectra of compound **IIb**.

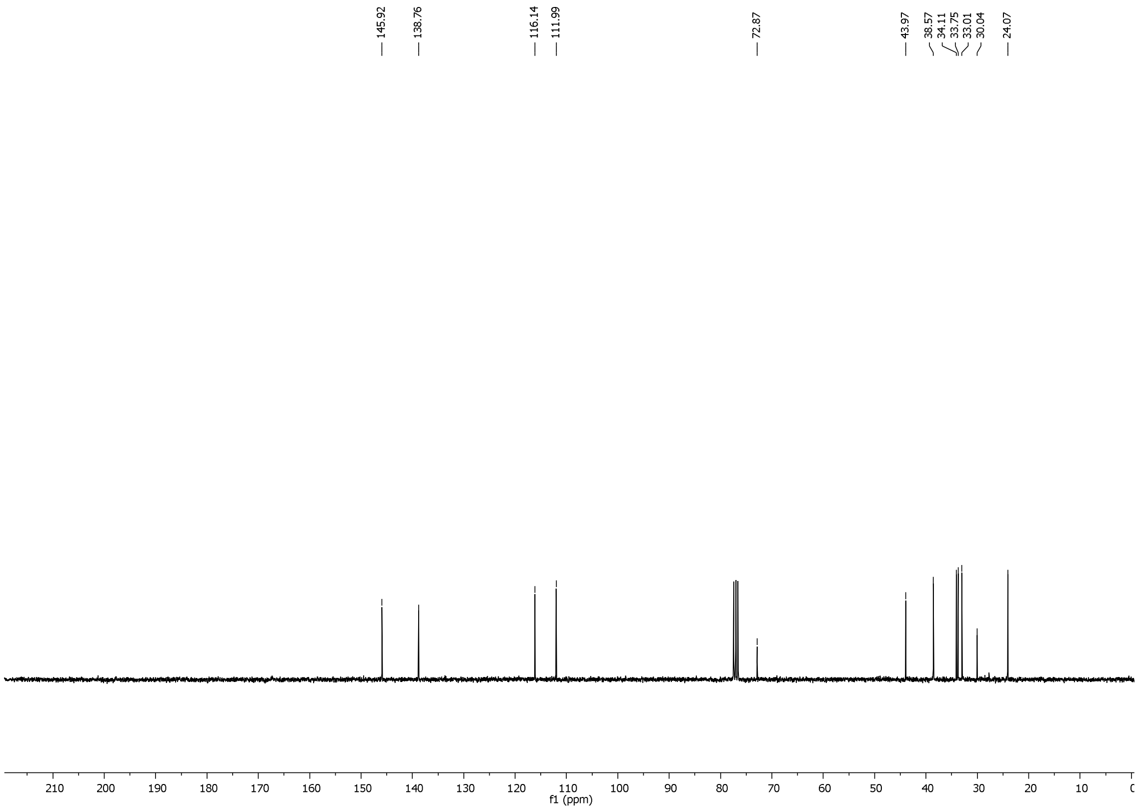

Figure SI-2. ^{13}C-NMR (75.5 MHz, $CDCl_3$) spectra of compound **IIb**.

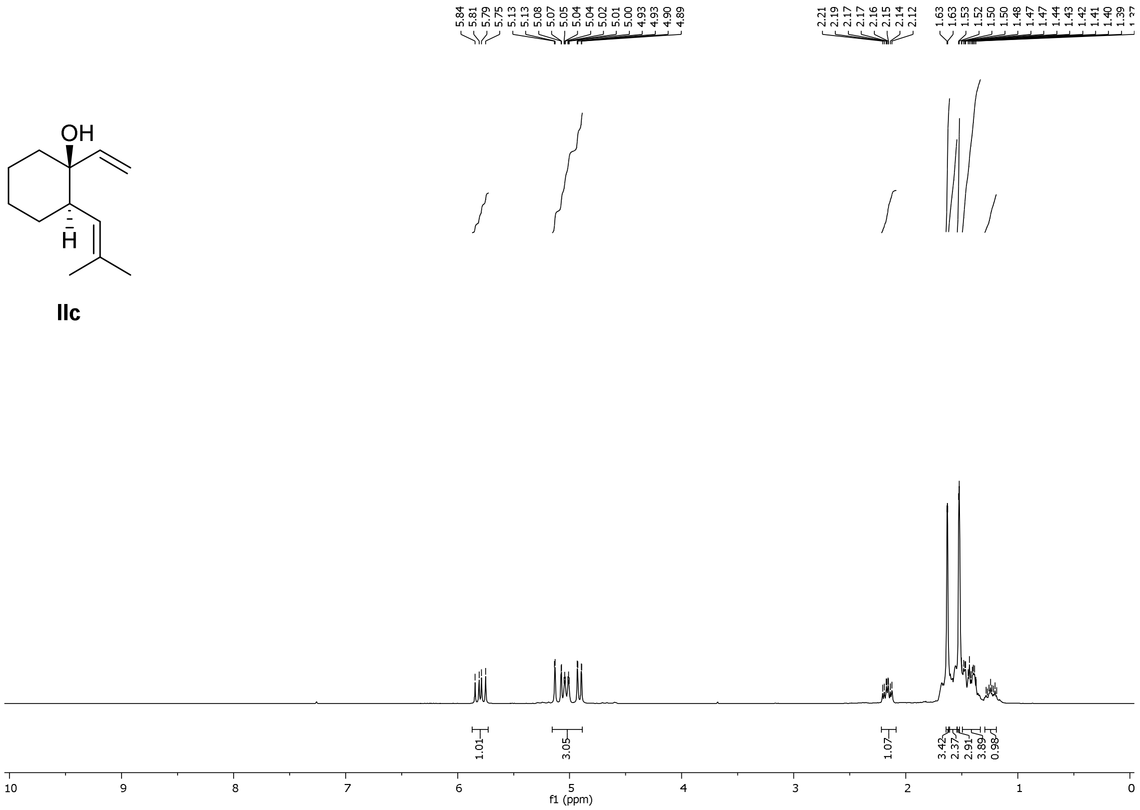

Figure SI-3. ^{1}H-NMR (300 MHz, $CDCl_3$) spectra of compound **IIc**.

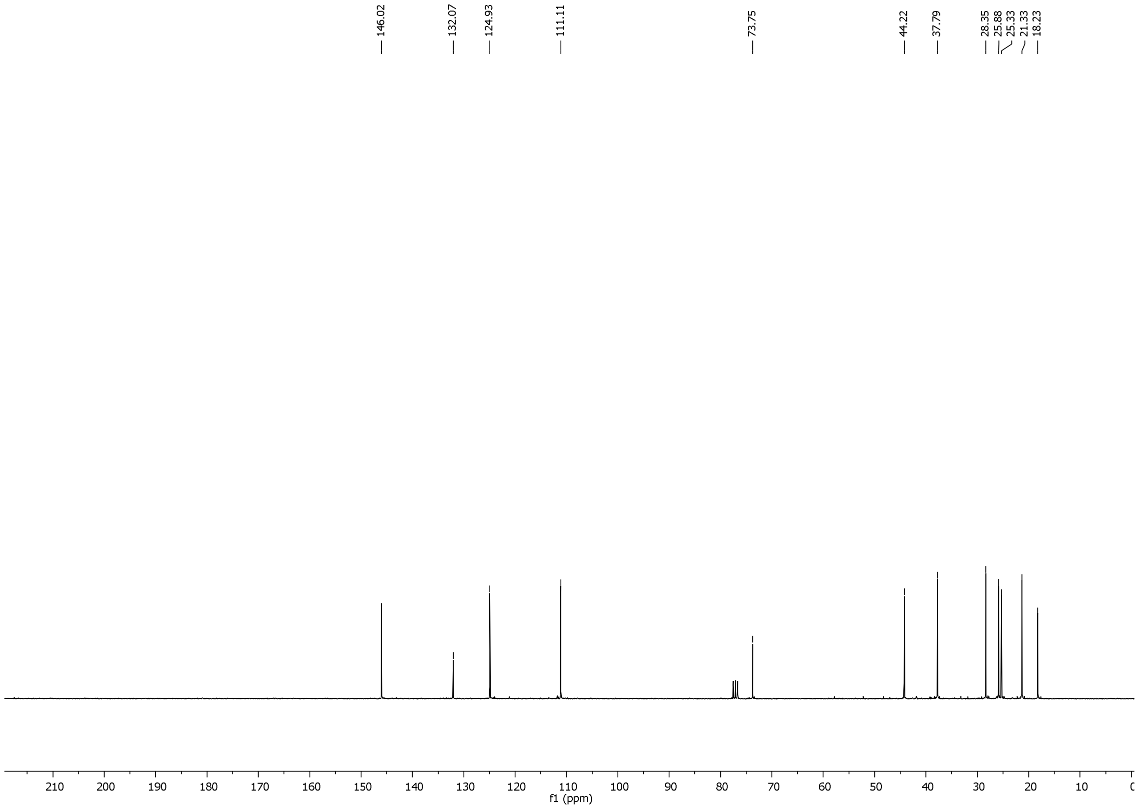

Figure SI-4. ^{13}C-NMR (75.5 MHz, $CDCl_3$) spectra of compound **IIc**.

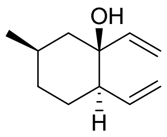

IIg

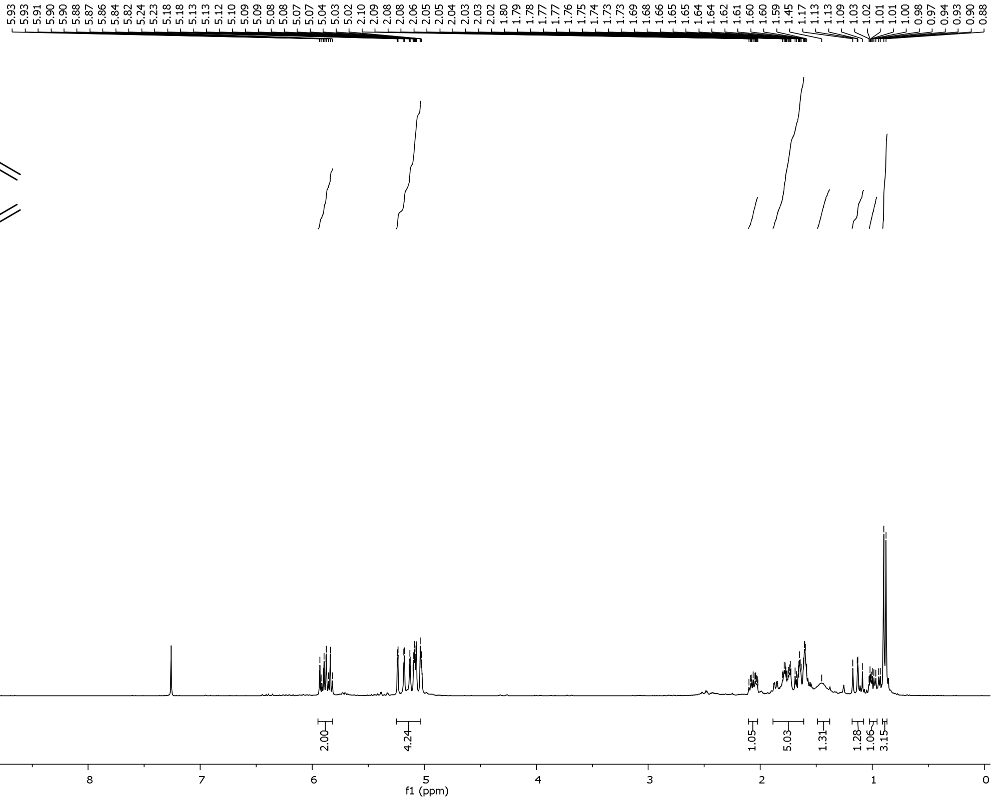

Figure SI-5. ^{1}H-NMR (300 MHz, $CDCl_3$) spectra of compound **IIg**.

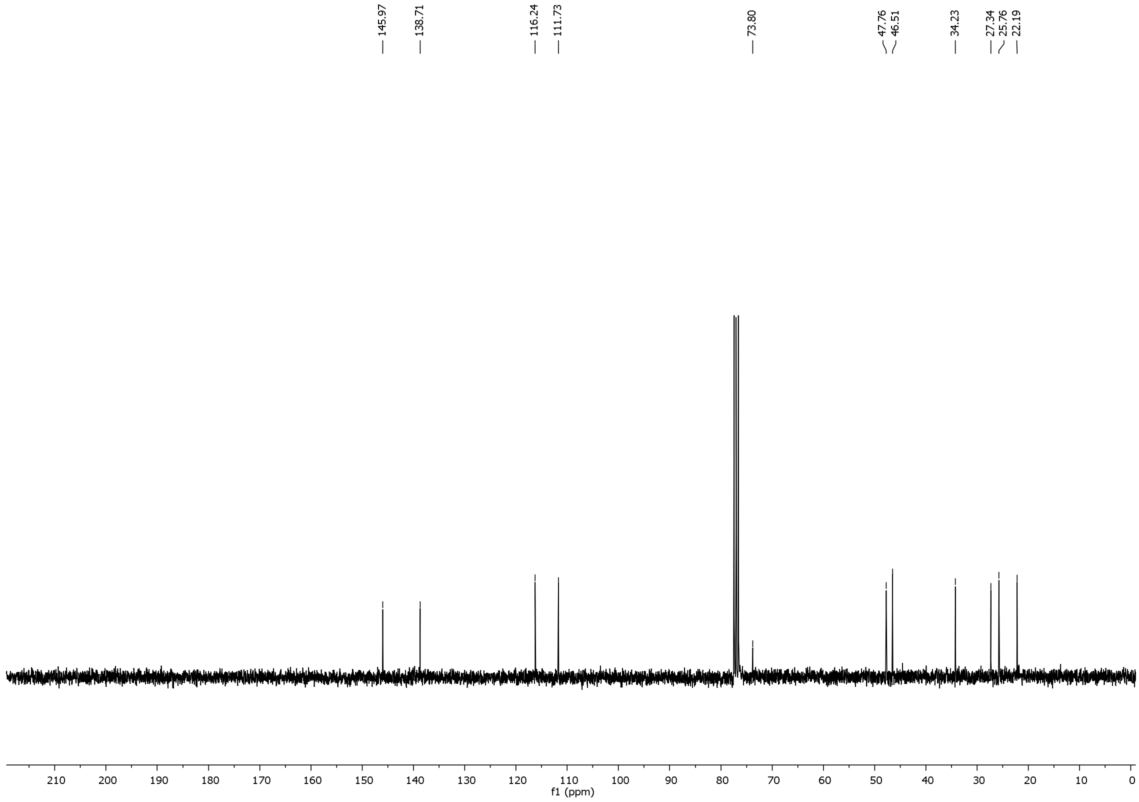

Figure SI-6. ^{13}C-NMR (75.5 MHz, $CDCl_3$) spectra of compound **IIg**.

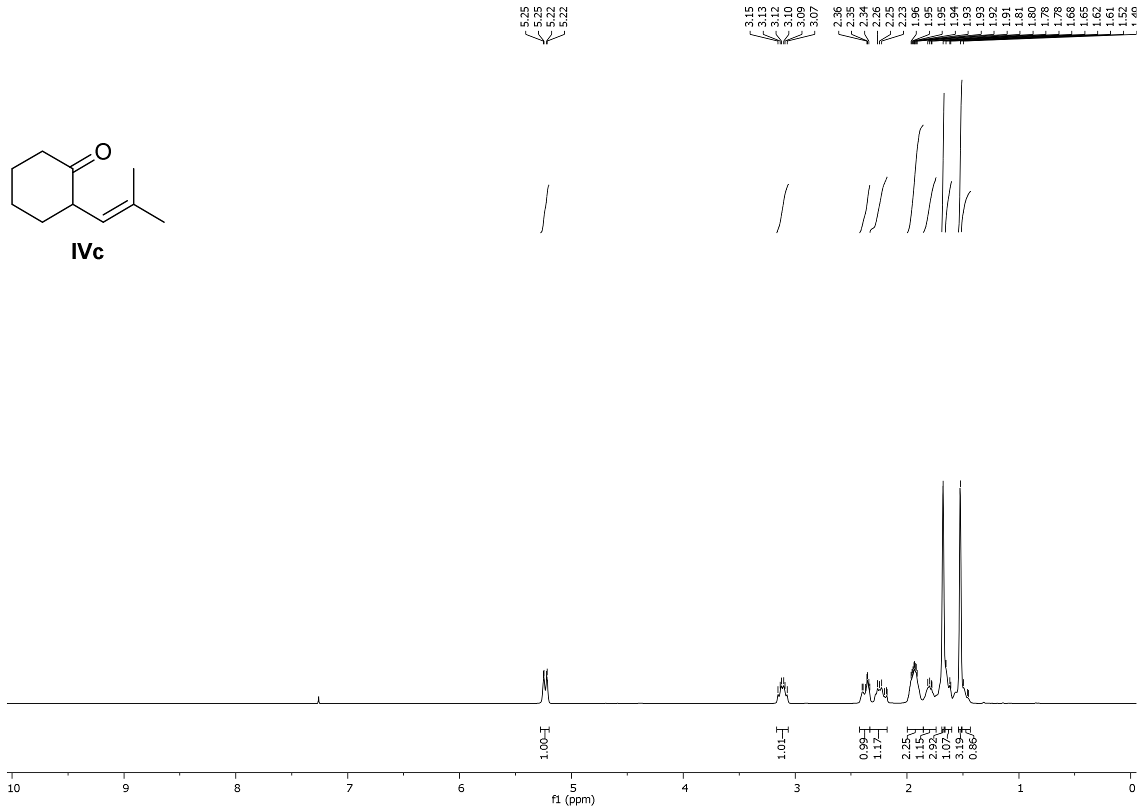

Figure SI-7. ^{1}H-NMR (300 MHz, $CDCl_3$) spectra of compound **IVc**.

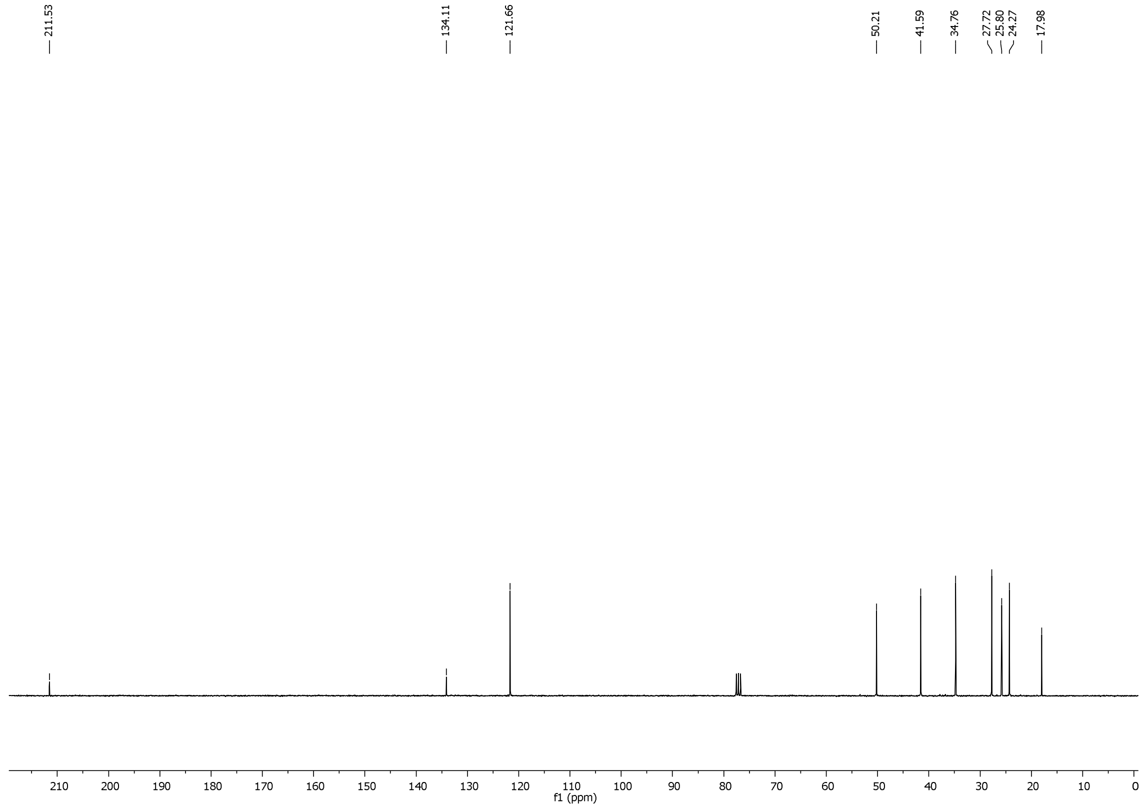

Figure SI-8. ^{13}C-NMR (75.5 MHz, $CDCl_3$) spectra of compound **IVc**.

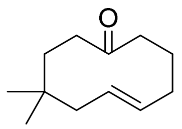

1b

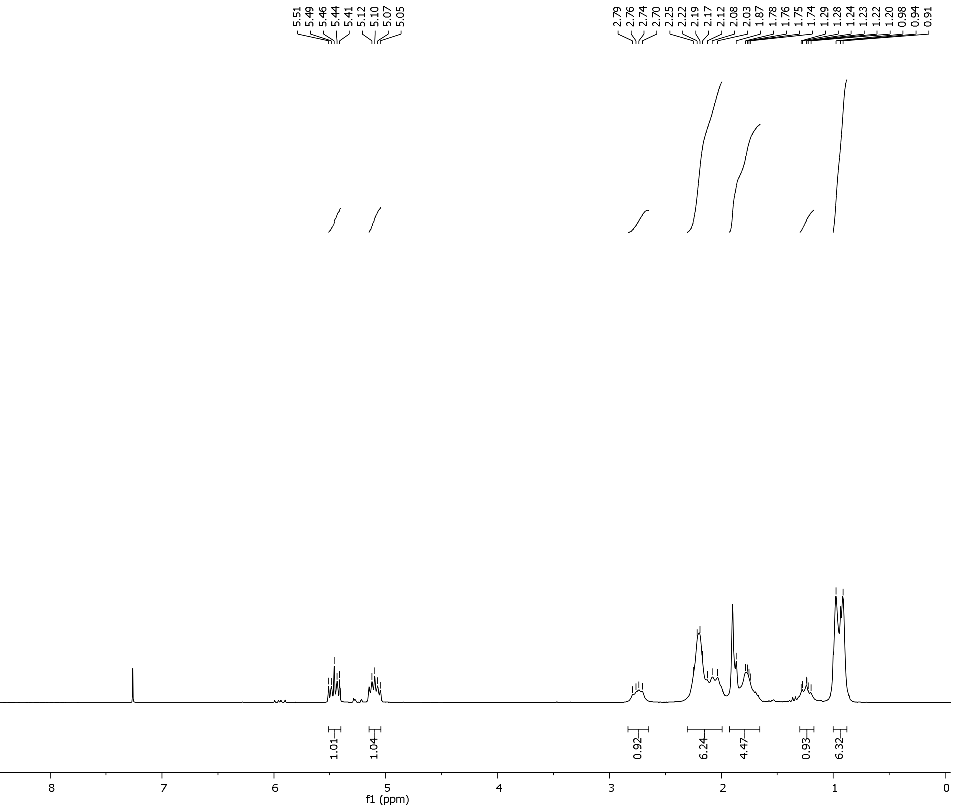

Figure SI-9. ^{1}H-NMR (300 MHz, $CDCl_3$) spectra of compound **1b**.

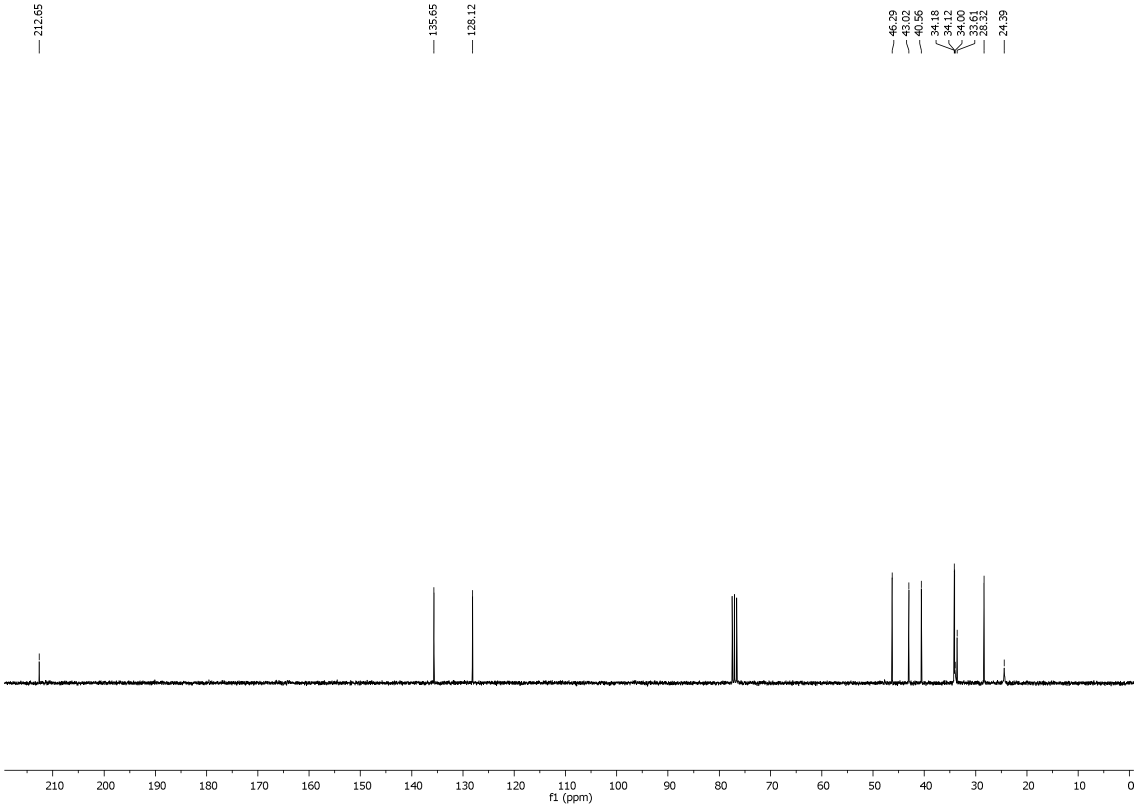

Figure SI-10. ^{13}C-NMR (75.5 MHz, $CDCl_3$) spectra of compound **1b**.

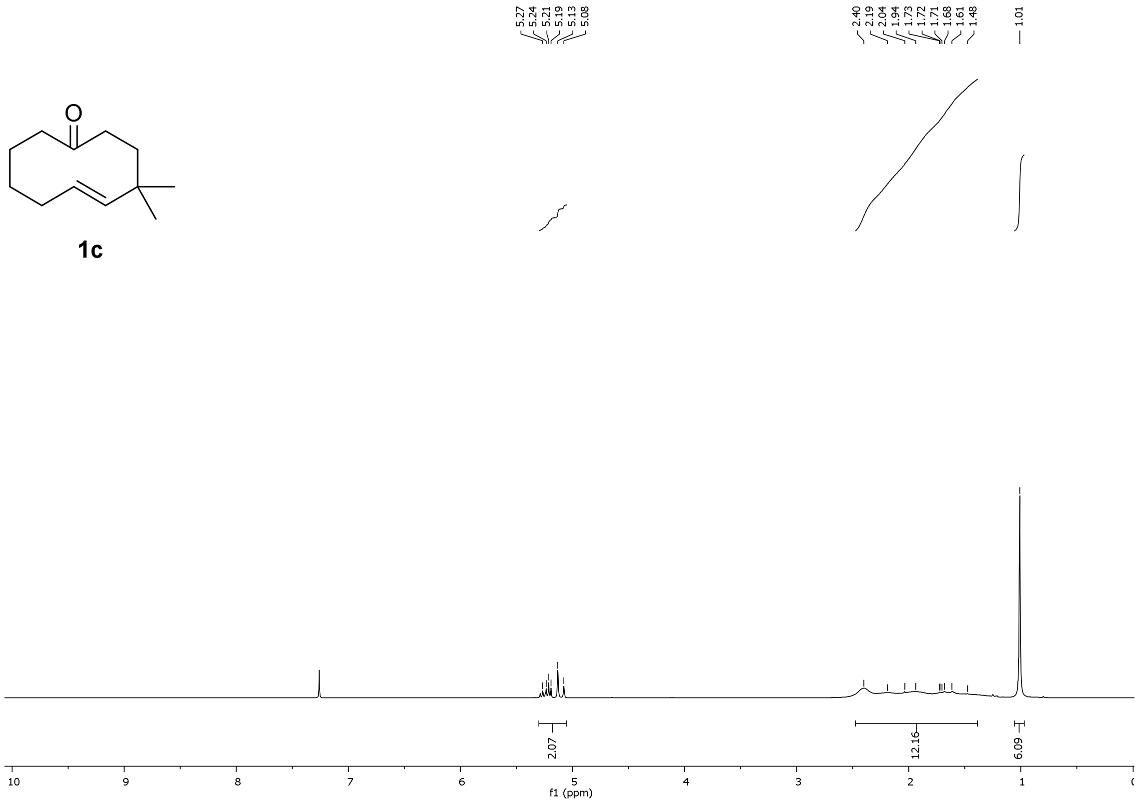

Figure SI-11. ^{1}H-NMR (300 MHz, $CDCl_3$) spectra of compound **1c**.

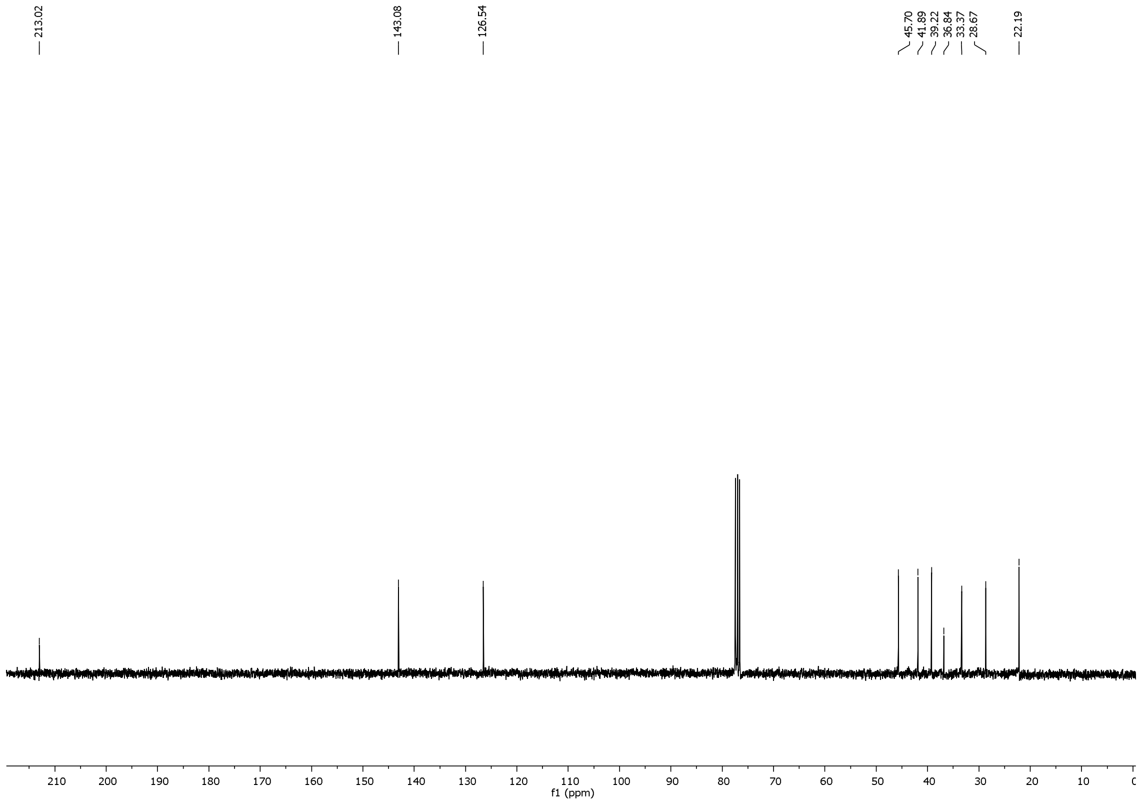

Figure SI-12. ^{13}C-NMR (75.5 MHz, $CDCl_3$) spectra of compound **1c**.

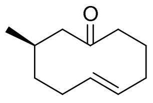

1g

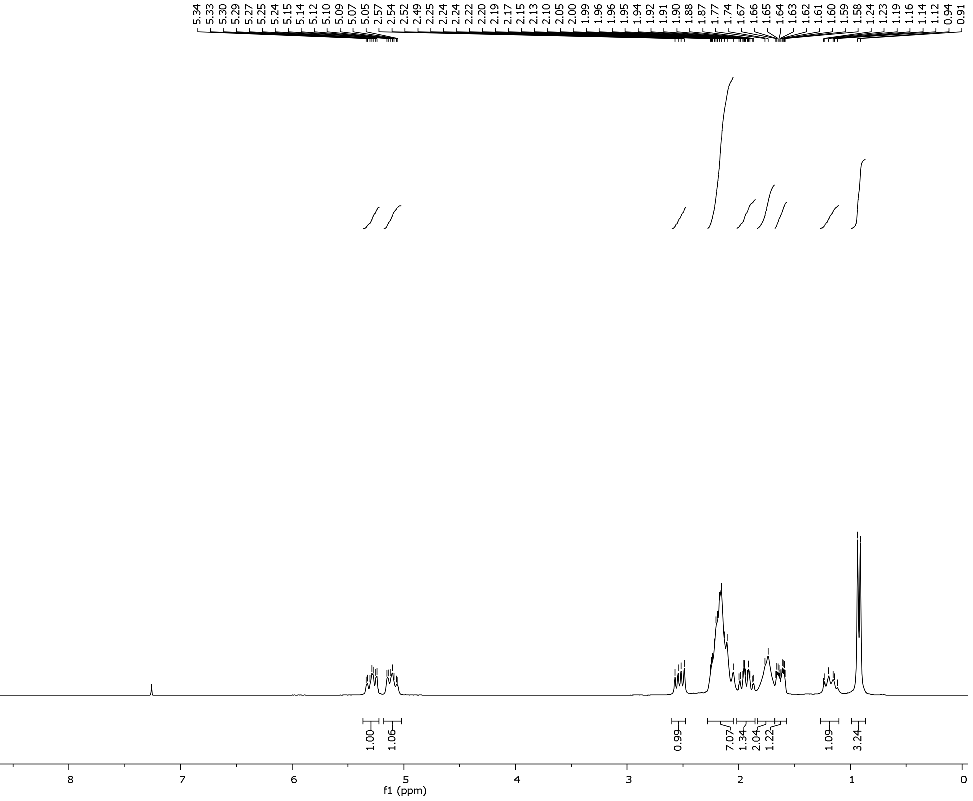

Figure SI-13. ^{1}H-NMR (300 MHz, $CDCl_3$) spectra of compound **1g**.

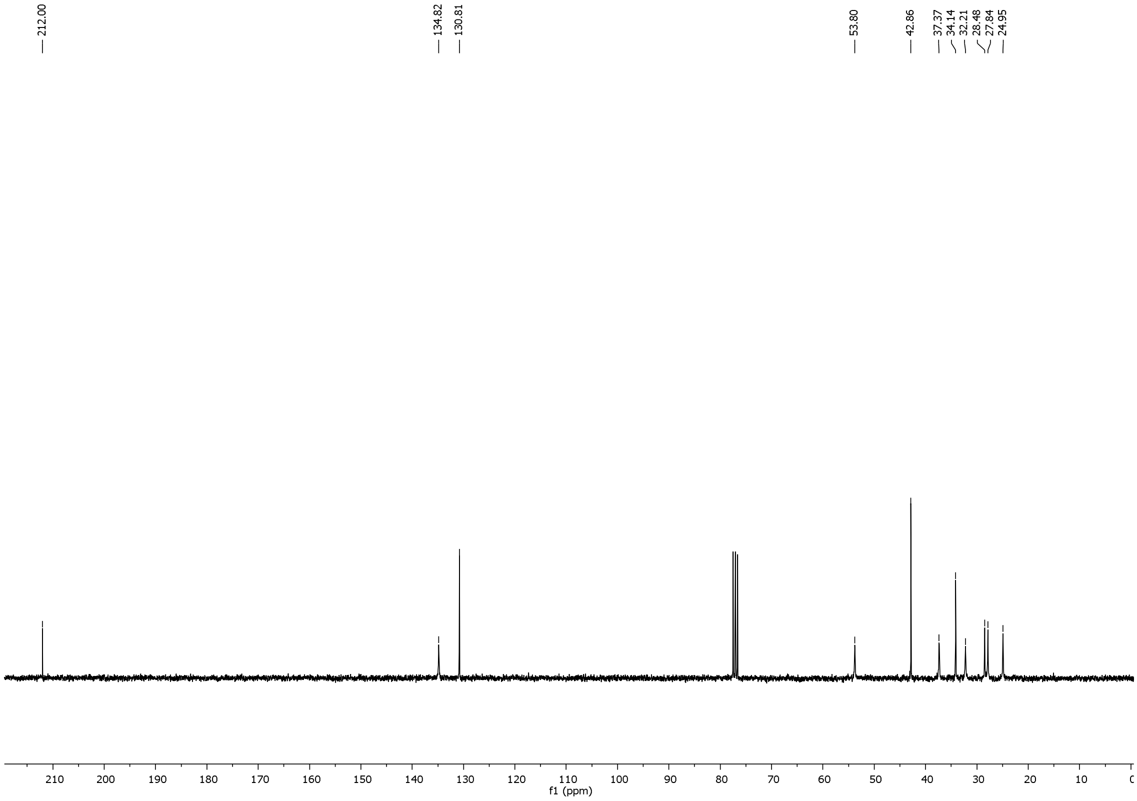

Figure SI-14. ^{13}C-NMR (75.5 MHz, $CDCl_3$) spectra of compound **1g**.

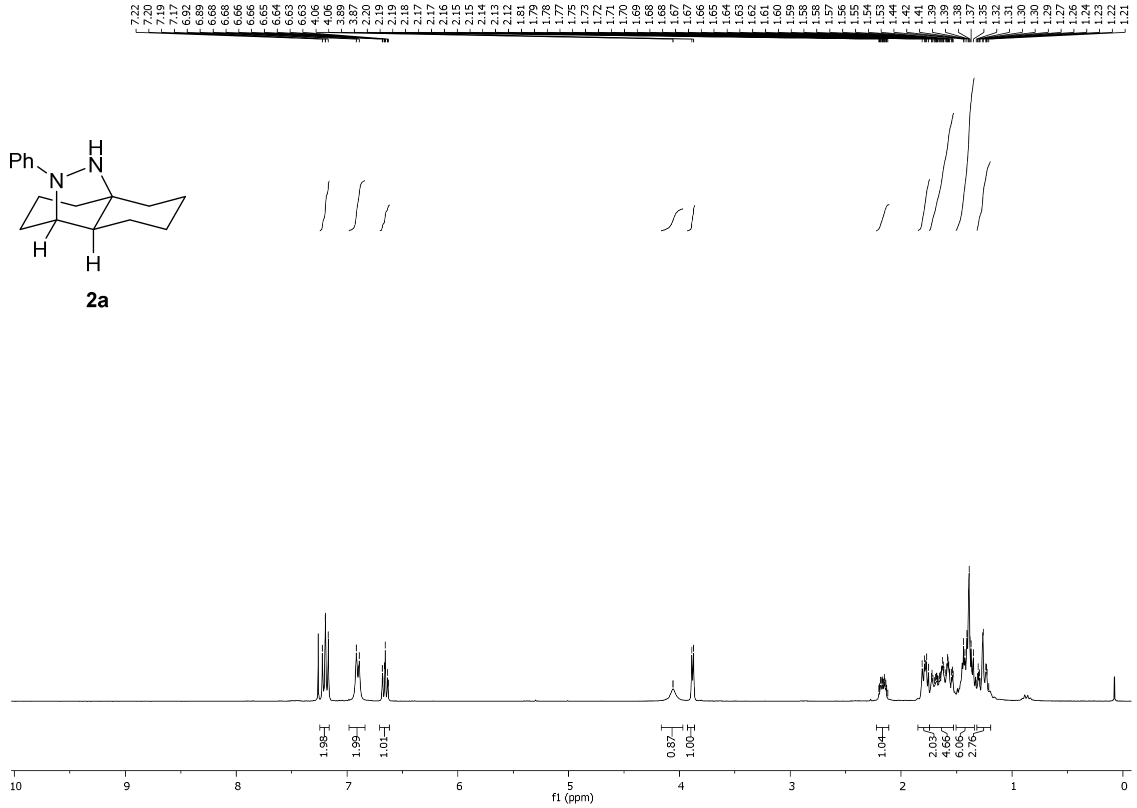

Figure SI-15. ^{1}H-NMR (300 MHz, $CDCl_3$) spectra of compound **2a**.

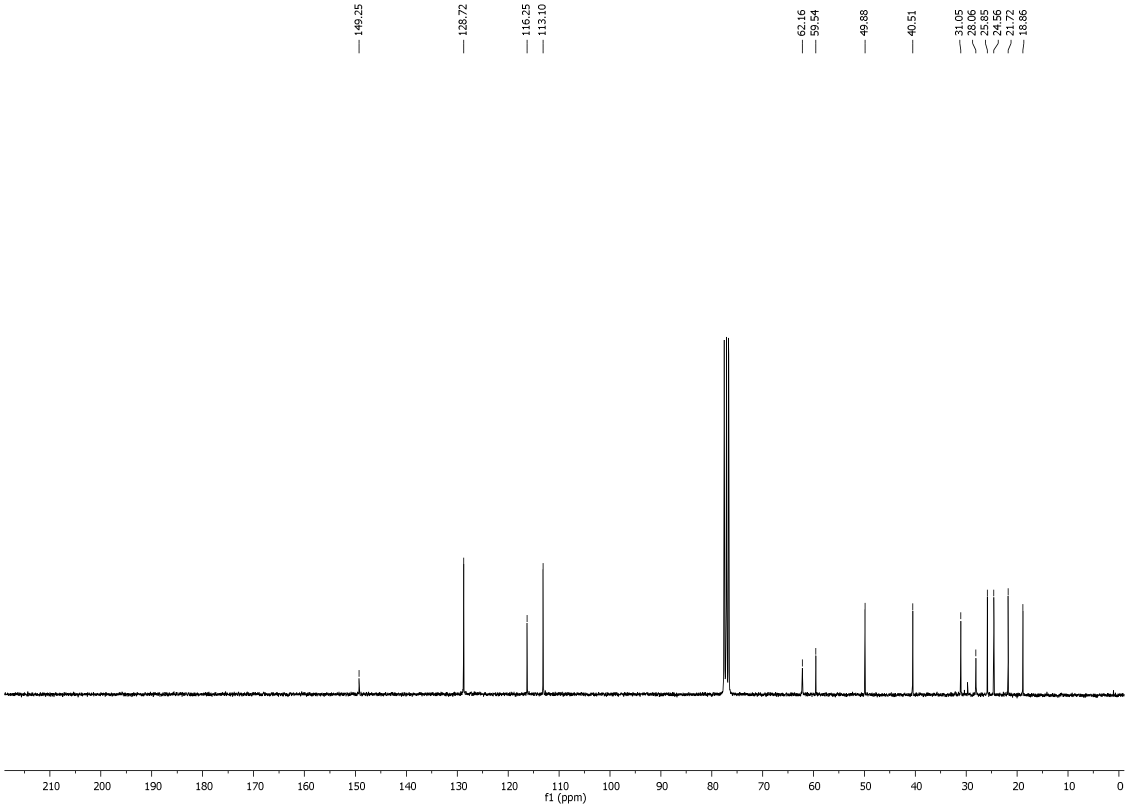

Figure SI-16. ^{13}C-NMR (75.5 MHz, $CDCl_3$) spectra of compound **2a**.

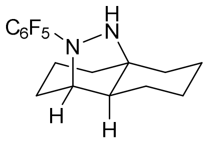

2b

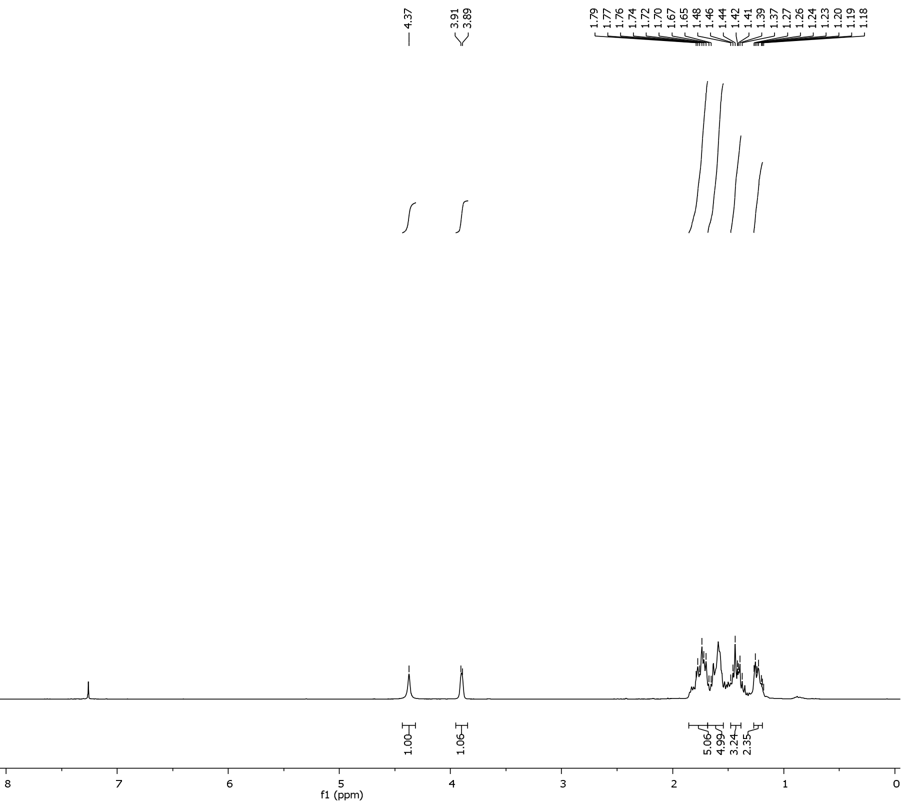

Figure SI-17. ^{1}H-NMR (300 MHz, $CDCl_3$) spectra of compound **2b**.

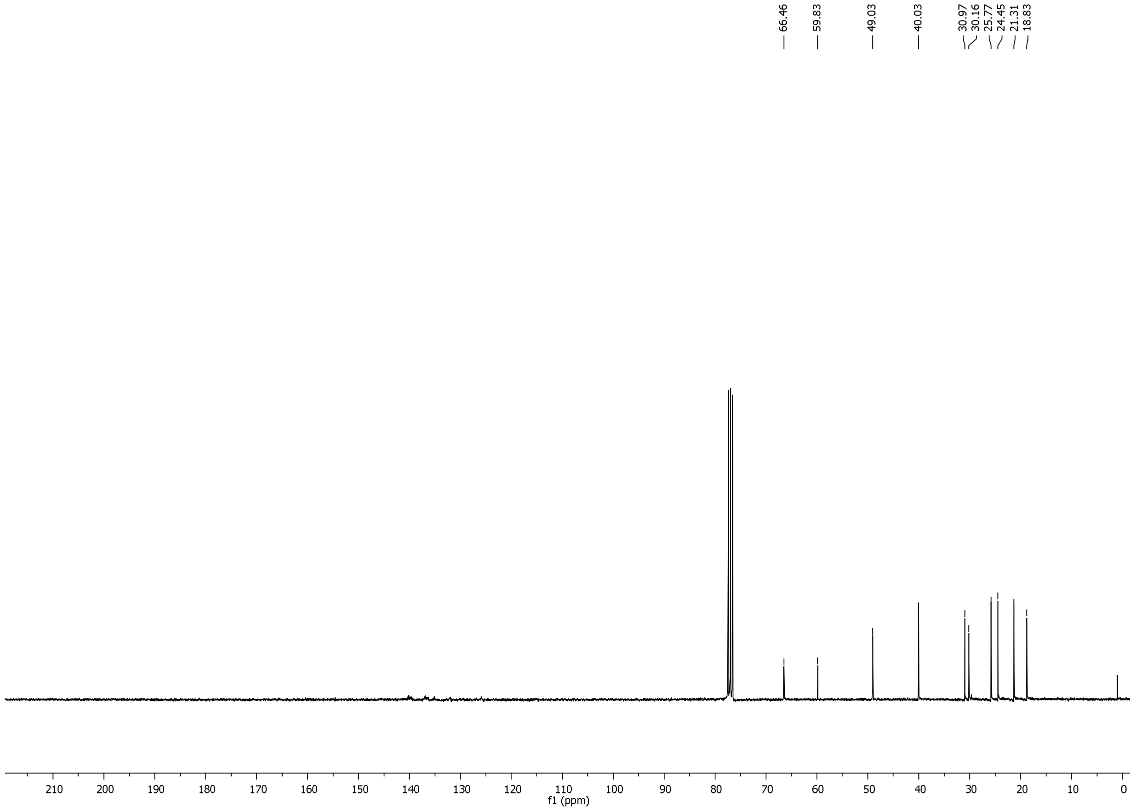

Figure SI-18. ^{13}C-NMR (75.5 MHz, $CDCl_3$) spectra of compound **2b**.

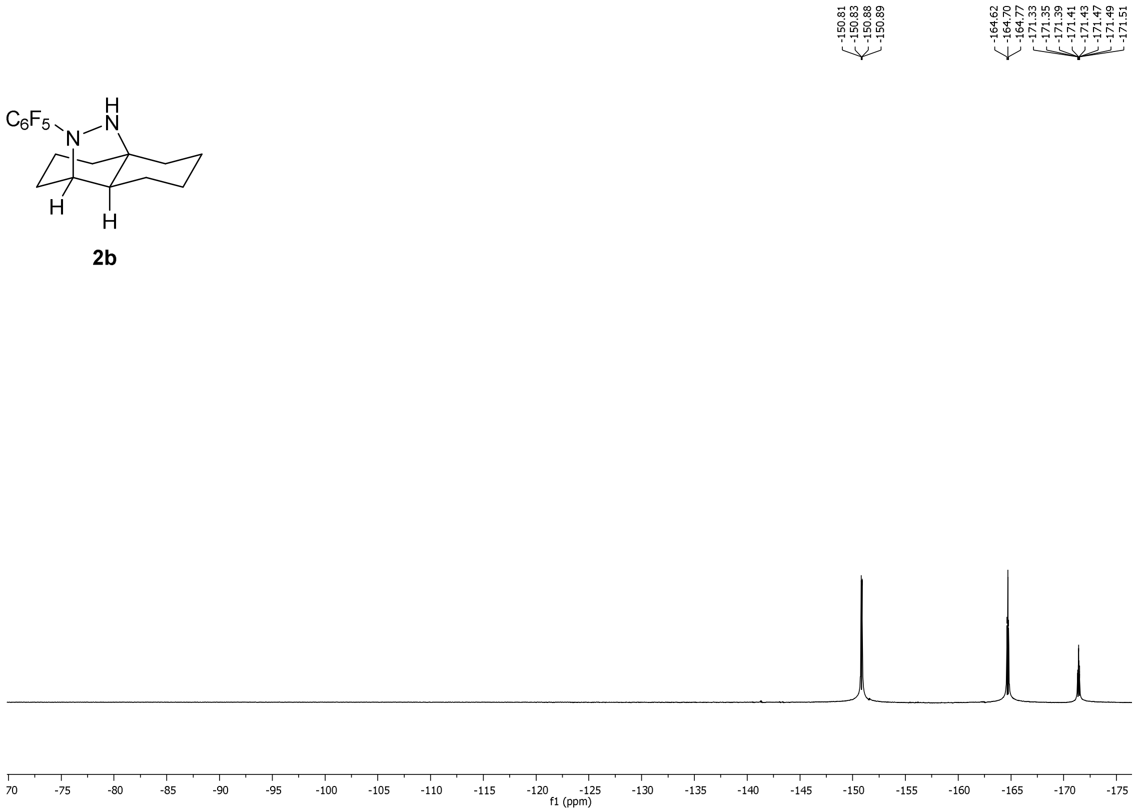

Figure SI-19. ^{19}F-NMR (282 MHz, $CDCl_3$) spectra of compound **2b**.

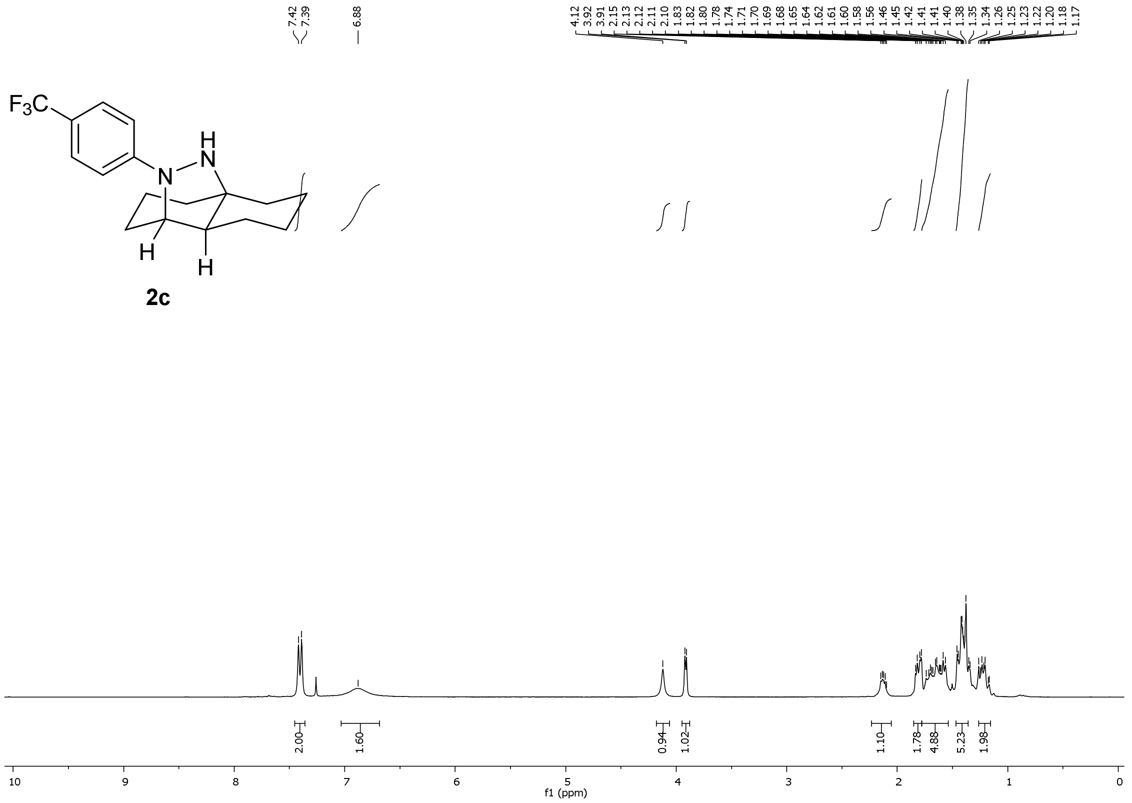

Figure SI-20. ^{1}H-NMR (300 MHz, $CDCl_3$) spectra of compound **2c**.

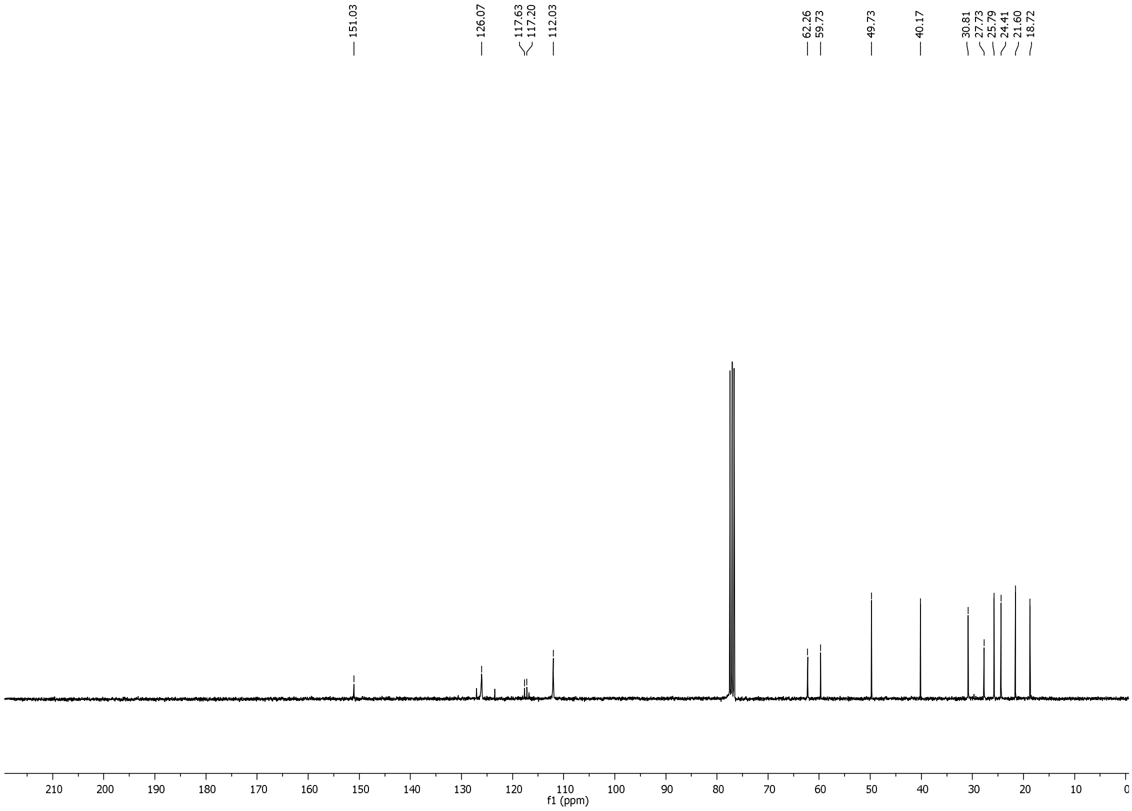

Figure SI-21. ^{13}C-NMR (75.5 MHz, $CDCl_3$) spectra of compound **2c**.

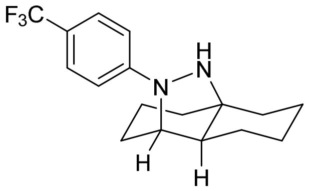

2c

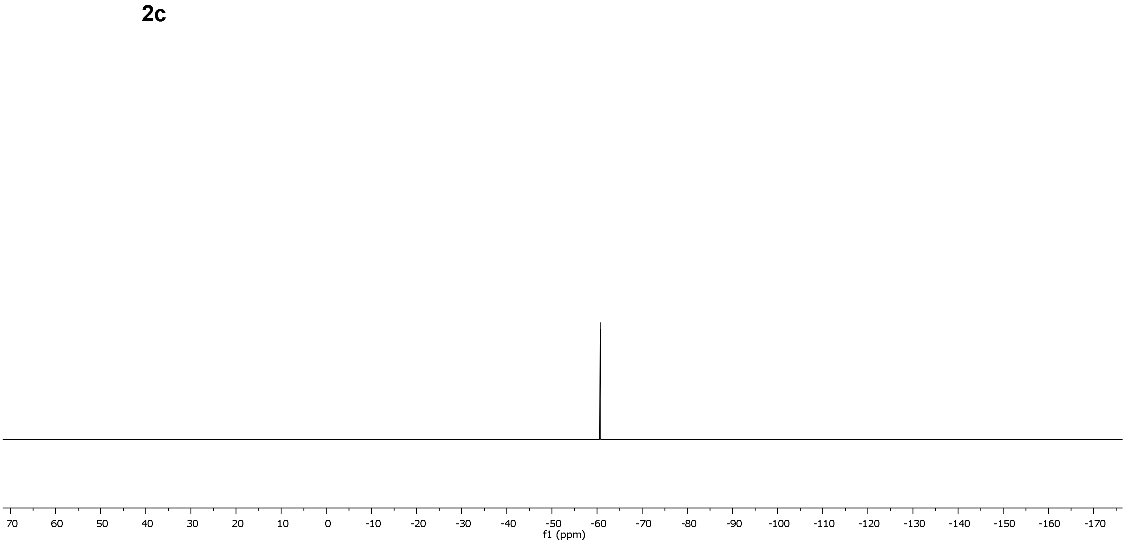

Figure SI-22. ^{19}F-NMR (282 MHz, $CDCl_3$) spectra of compound **2c**.

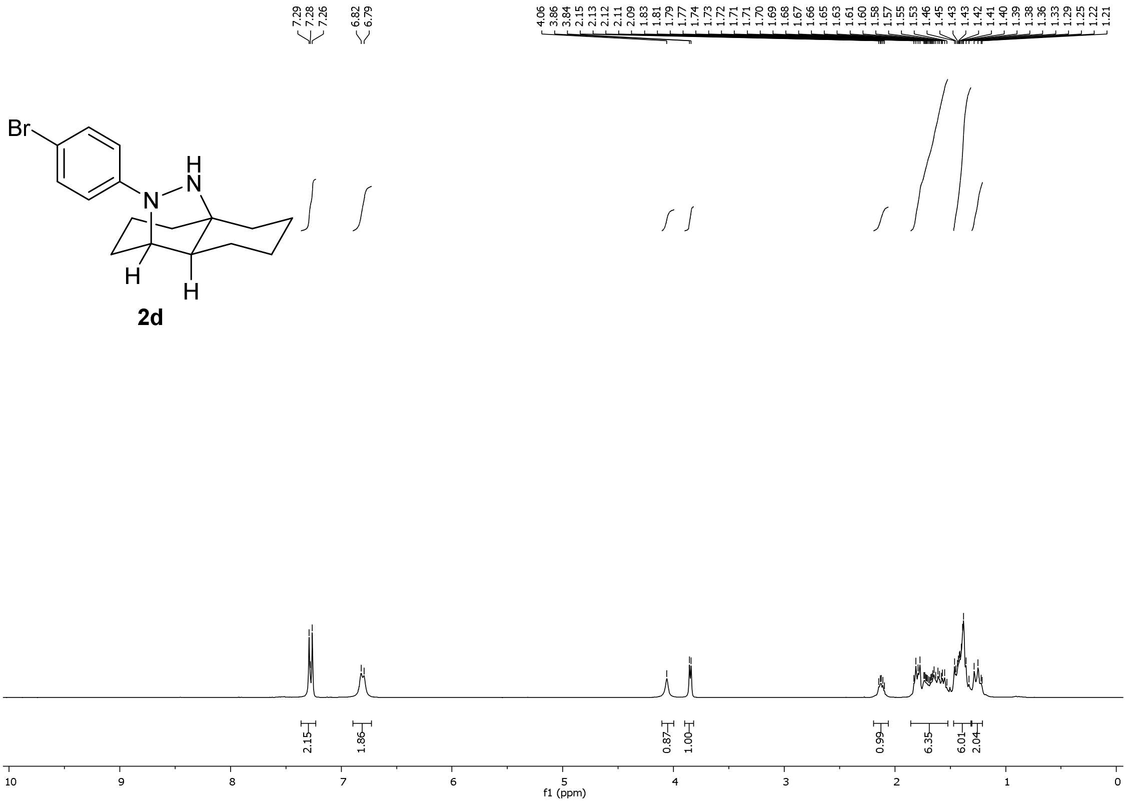

Figure SI-23. ^{1}H-NMR (300 MHz, $CDCl_3$) spectra of compound **2d**.

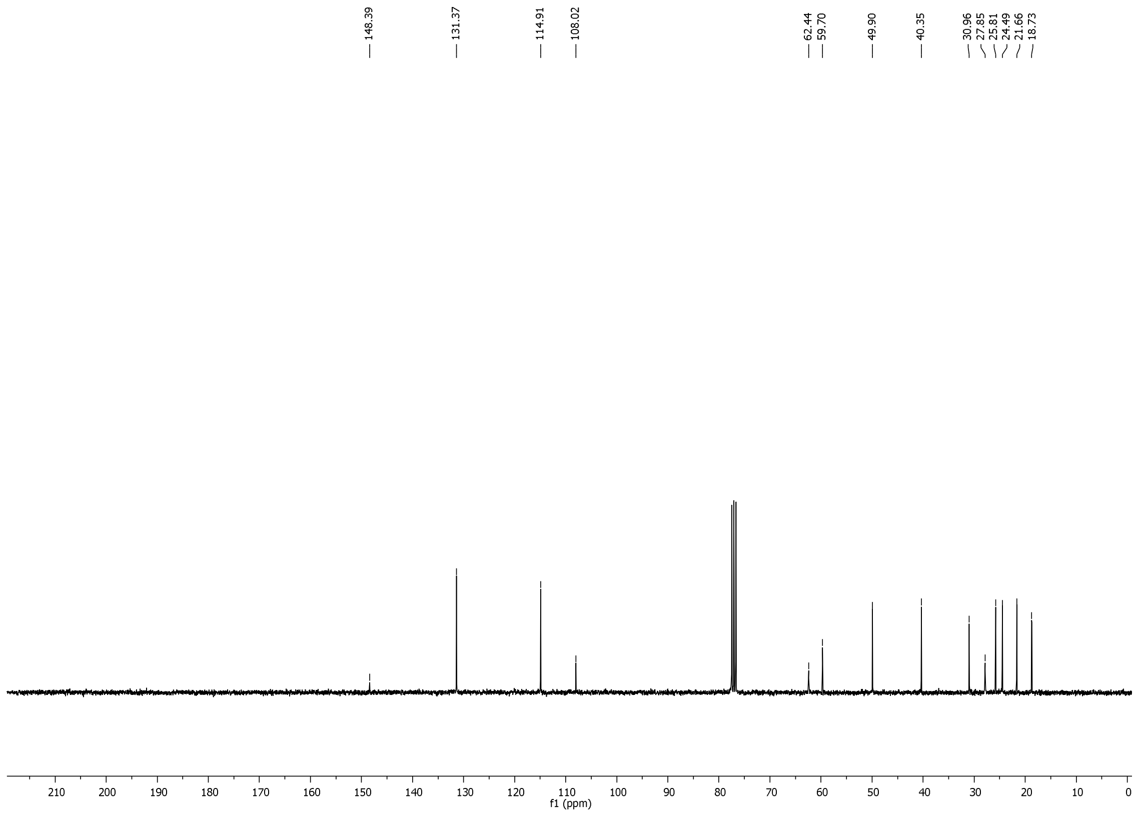

Figure SI-24. ^{13}C-NMR (75.5 MHz, $CDCl_3$) spectra of compound **2d**.

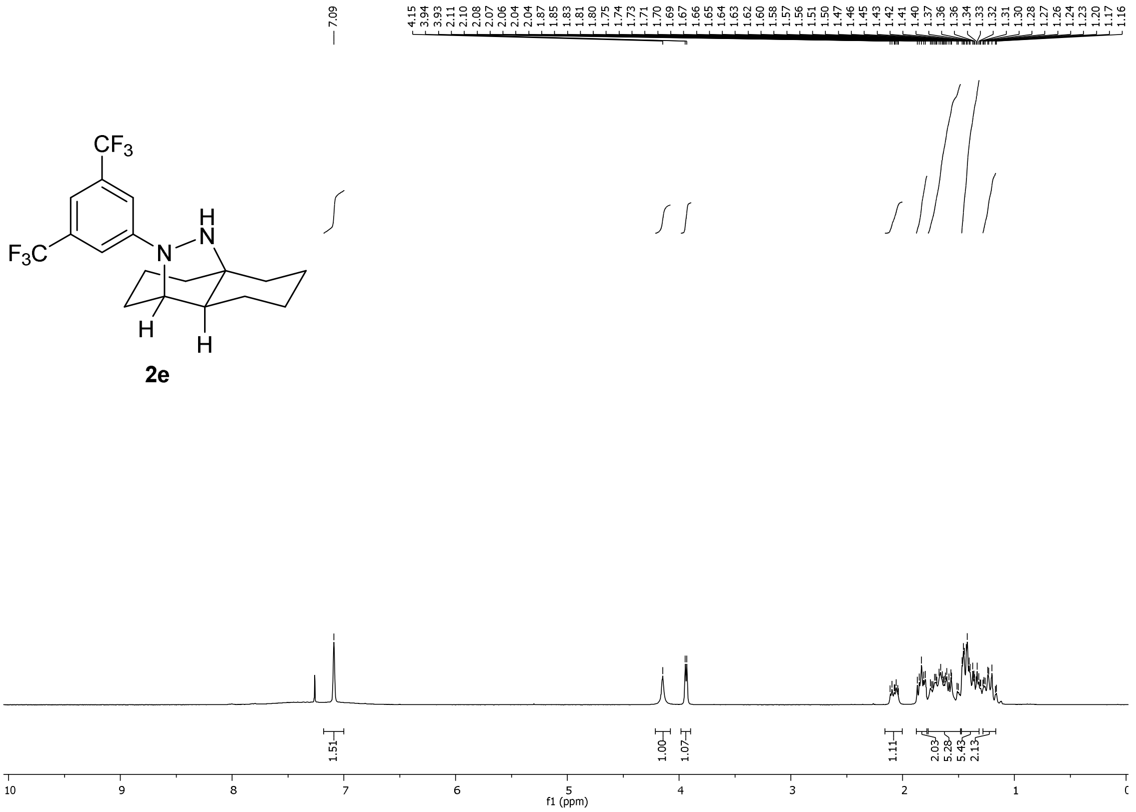

Figure SI-25. ^{1}H-NMR (300 MHz, $CDCl_3$) spectra of compound **2e**.

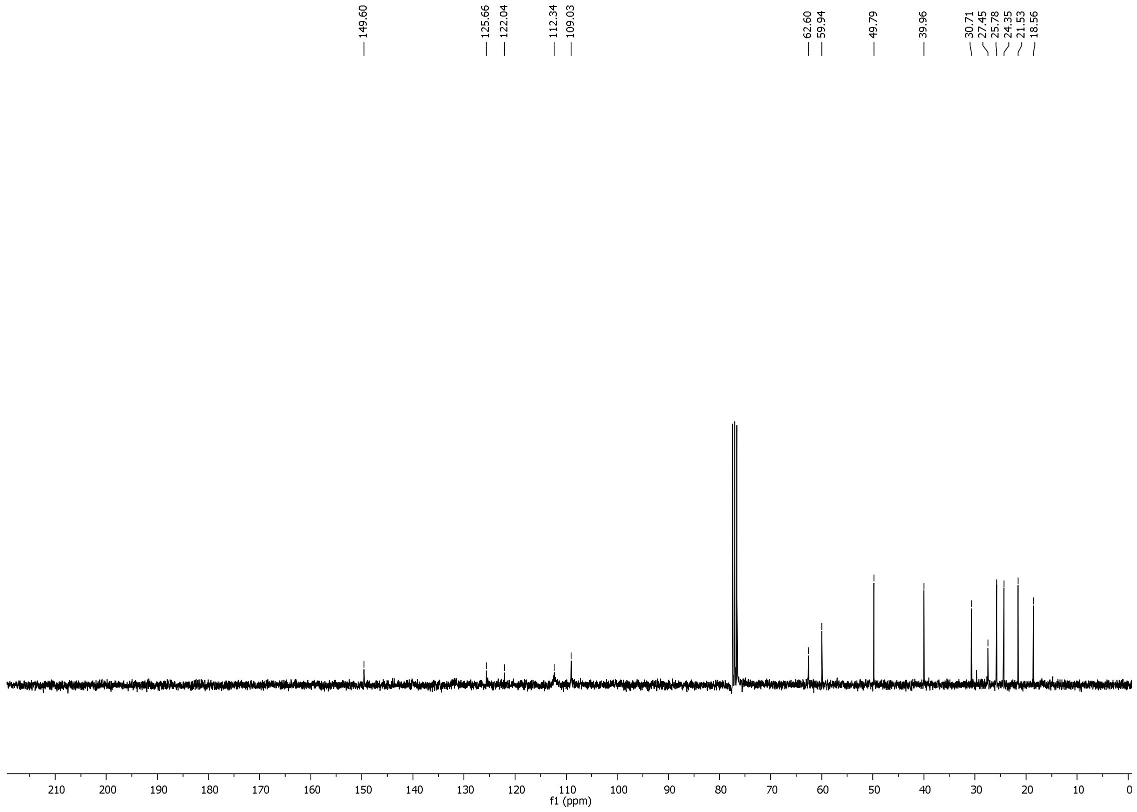

Figure SI-26. ^{13}C-NMR (75.5 MHz, $CDCl_3$) spectra of compound **2e**.

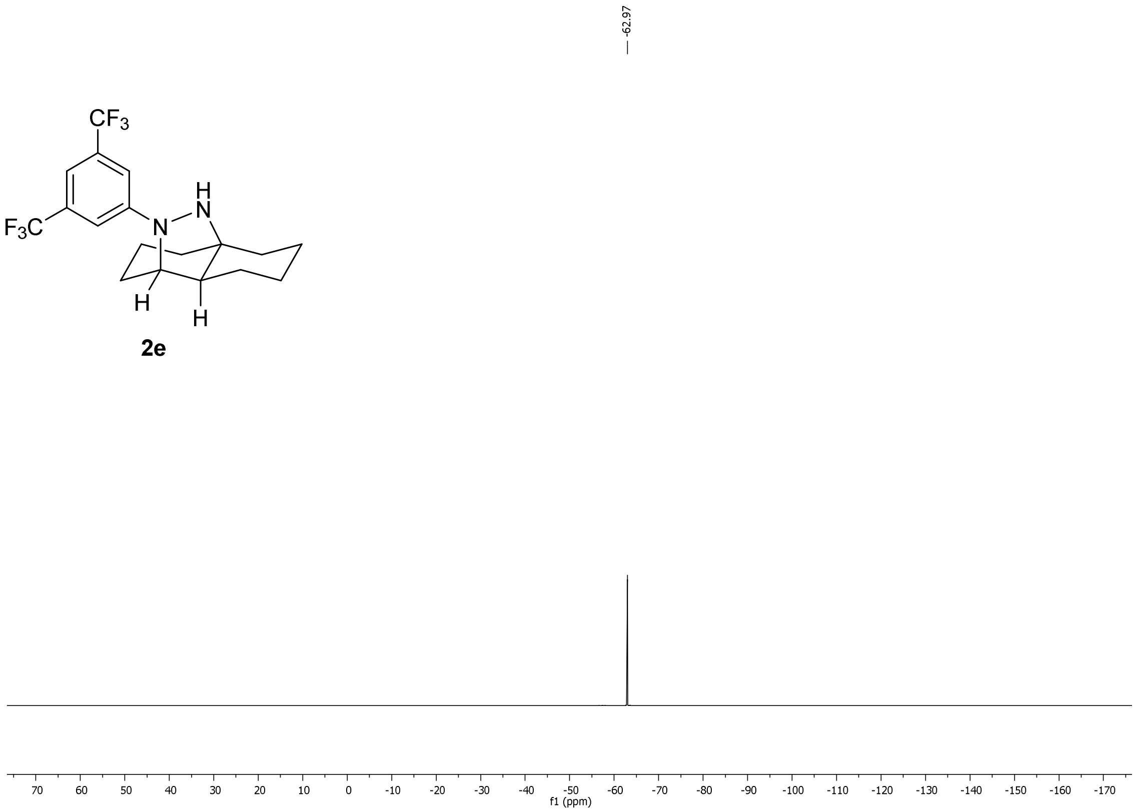

Figure SI-27. ^{19}F-NMR (282 MHz, $CDCl_3$) spectra of compound **2e**.

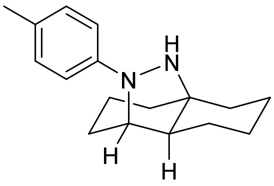

2f

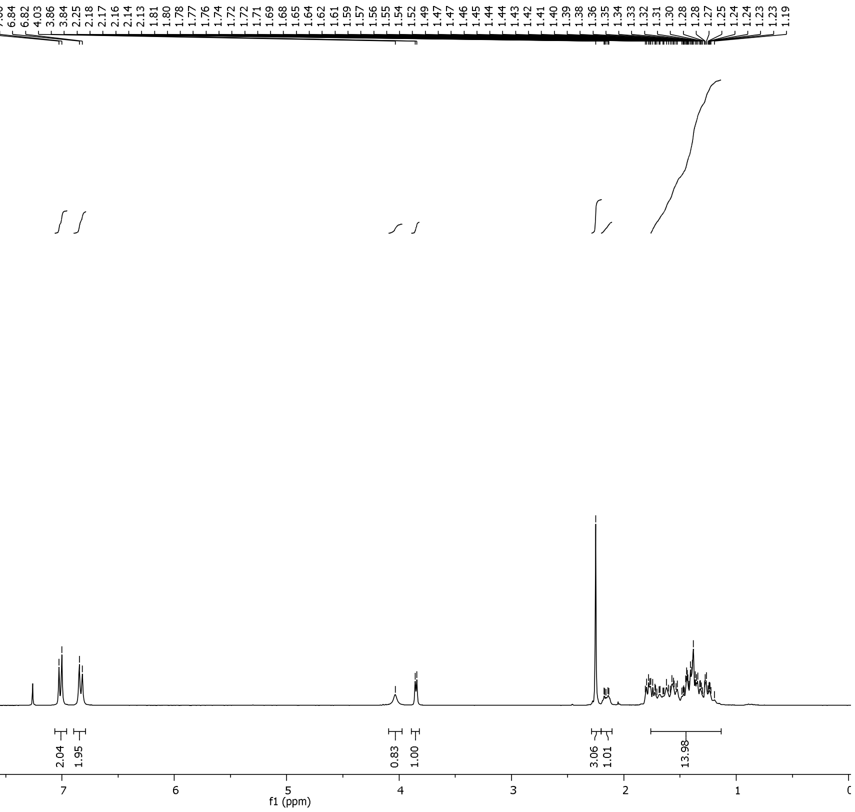

Figure SI-28. ^{1}H-NMR (300 MHz, $CDCl_3$) spectra of compound **2f**.

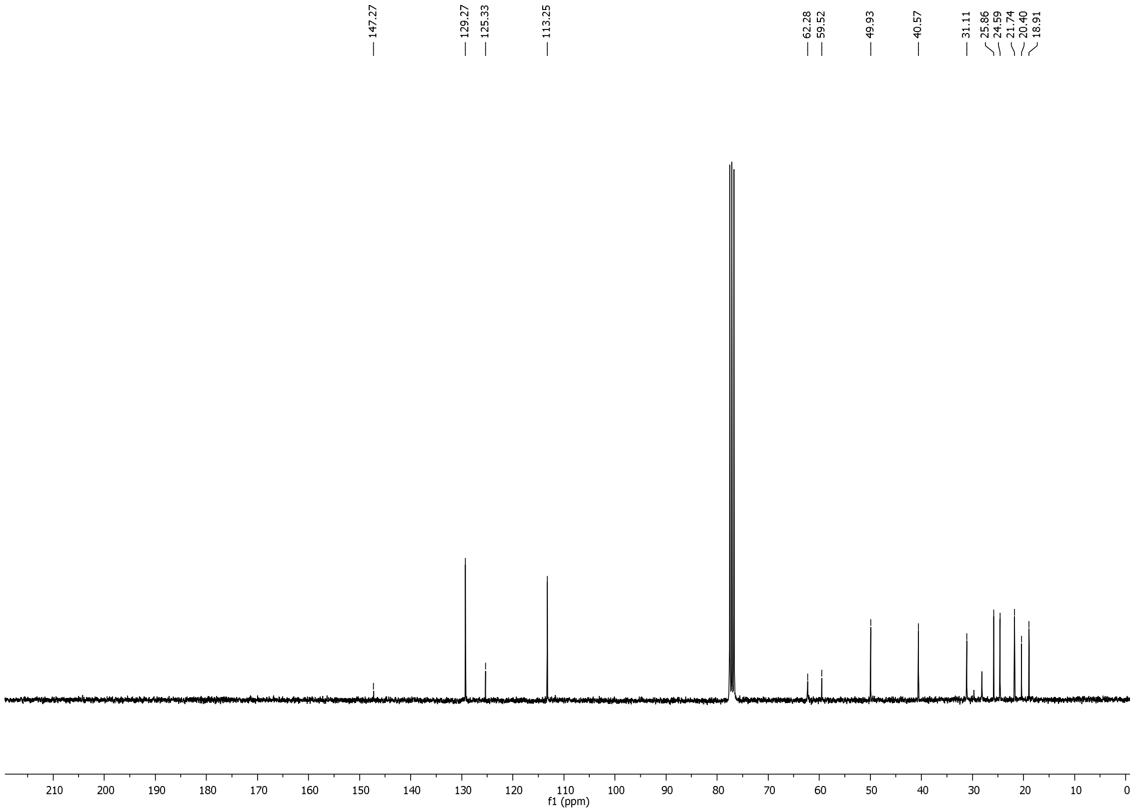

Figure SI-29. ^{13}C-NMR (75.5 MHz, $CDCl_3$) spectra of compound **2f**.

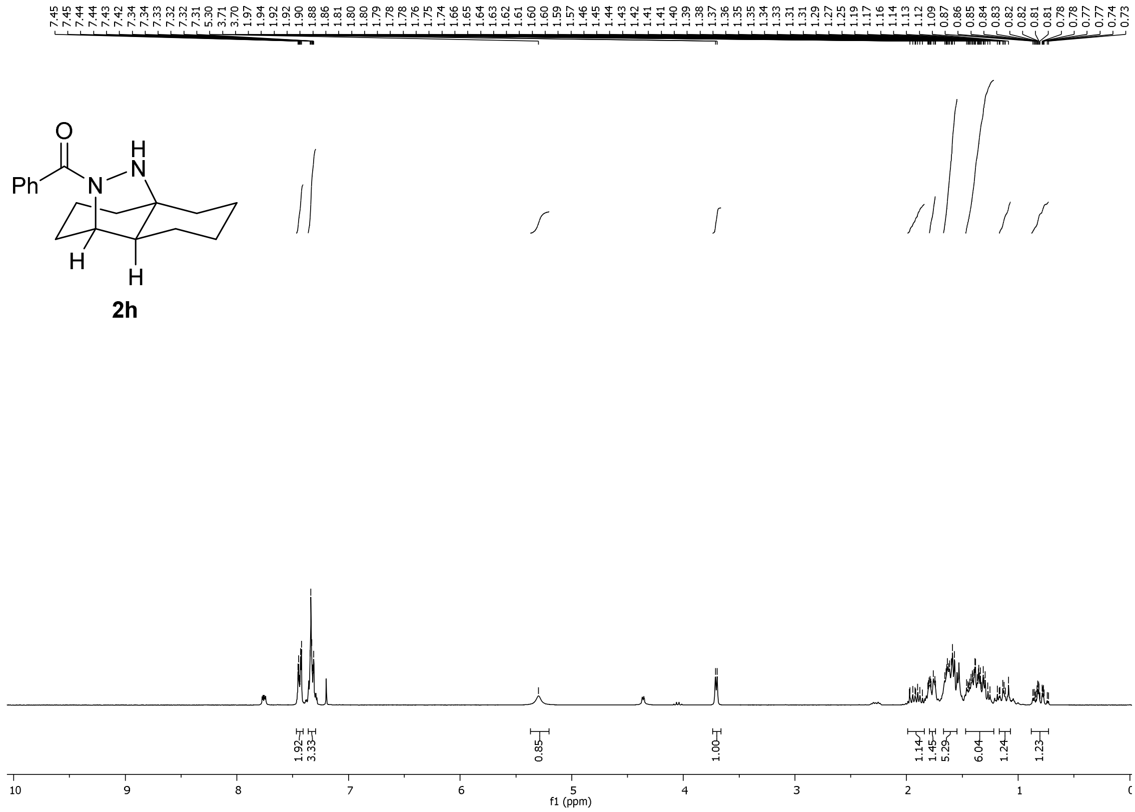

Figure SI-30. ^{1}H-NMR (300 MHz, $CDCl_3$) spectra of compound **2h**.

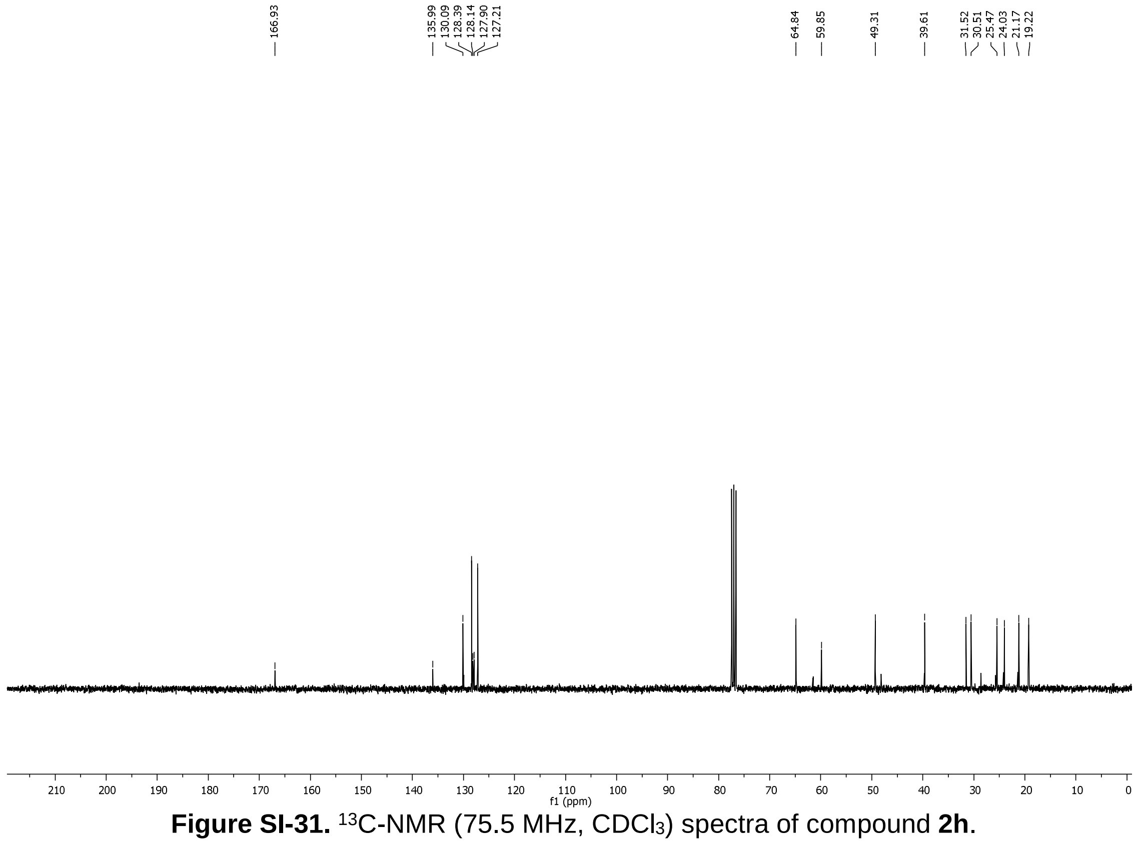

Figure SI-31. ^{13}C-NMR (75.5 MHz, $CDCl_3$) spectra of compound **2h**.

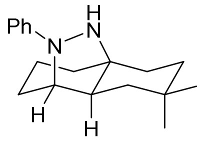

2i

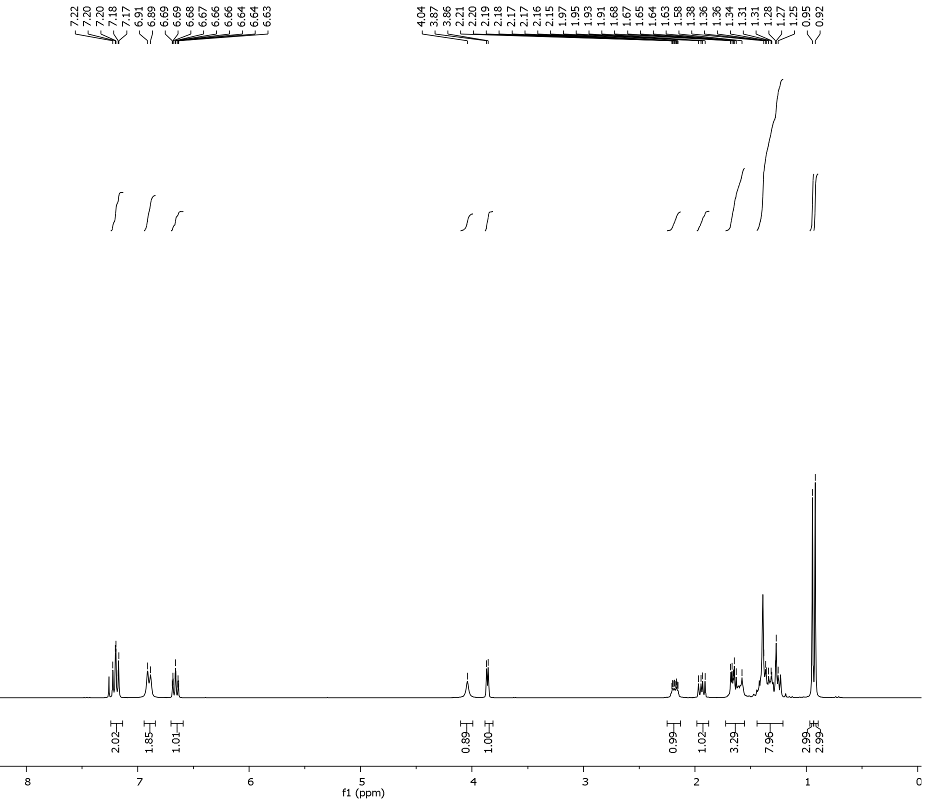

Figure SI-32. ^{1}H-NMR (300 MHz, $CDCl_3$) spectra of compound **2i**.

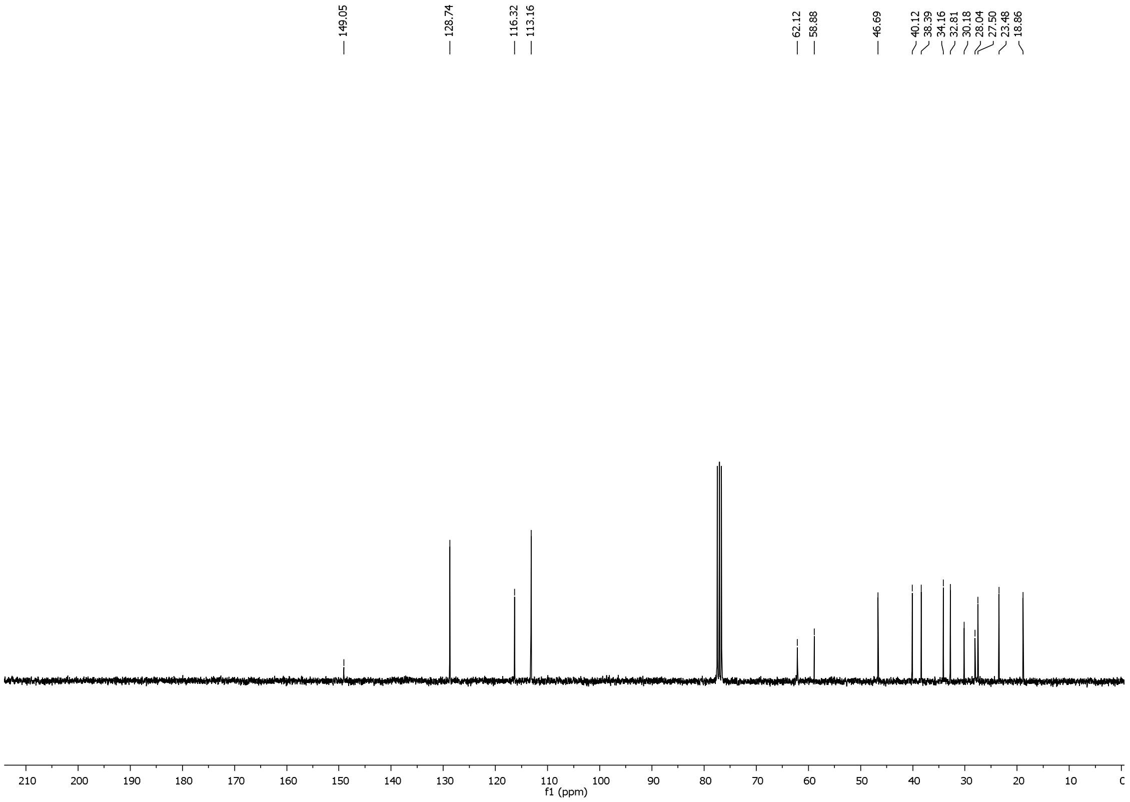

Figure SI-33. ^{13}C-NMR (75.5 MHz, $CDCl_3$) spectra of compound **2i**.

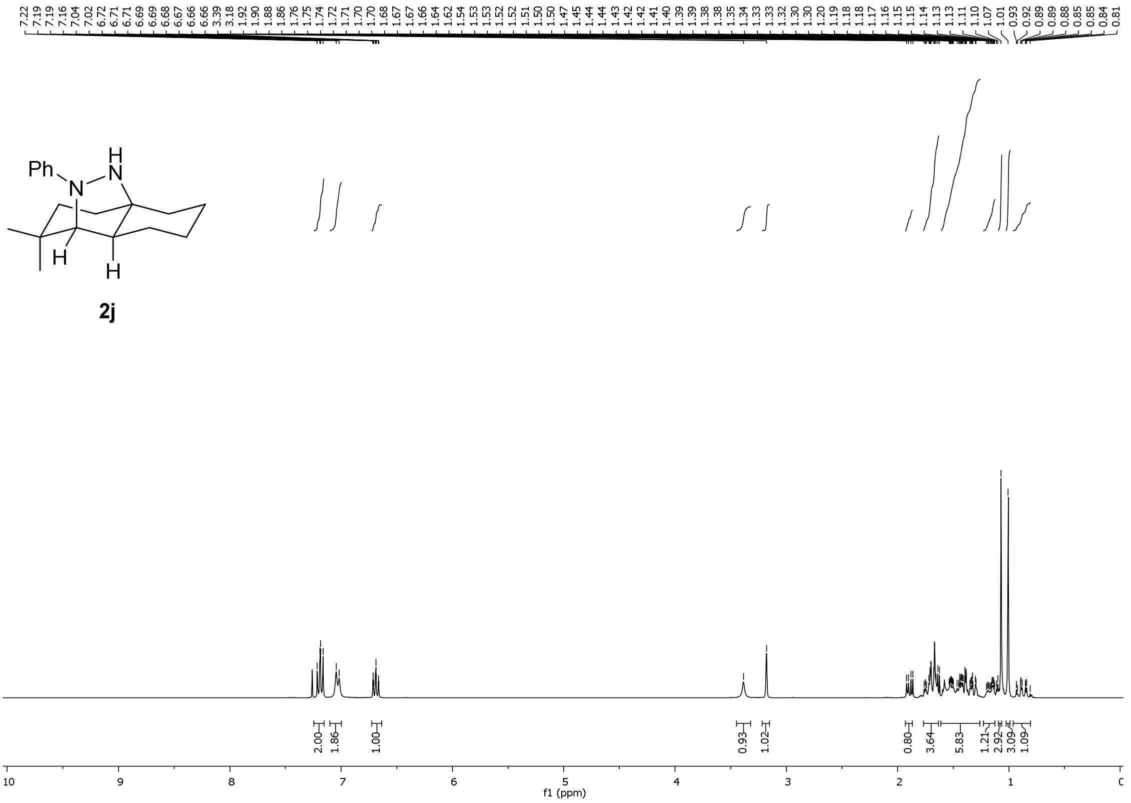

Figure SI-34. ^{1}H-NMR (300 MHz, $CDCl_3$) spectra of compound **2j**.

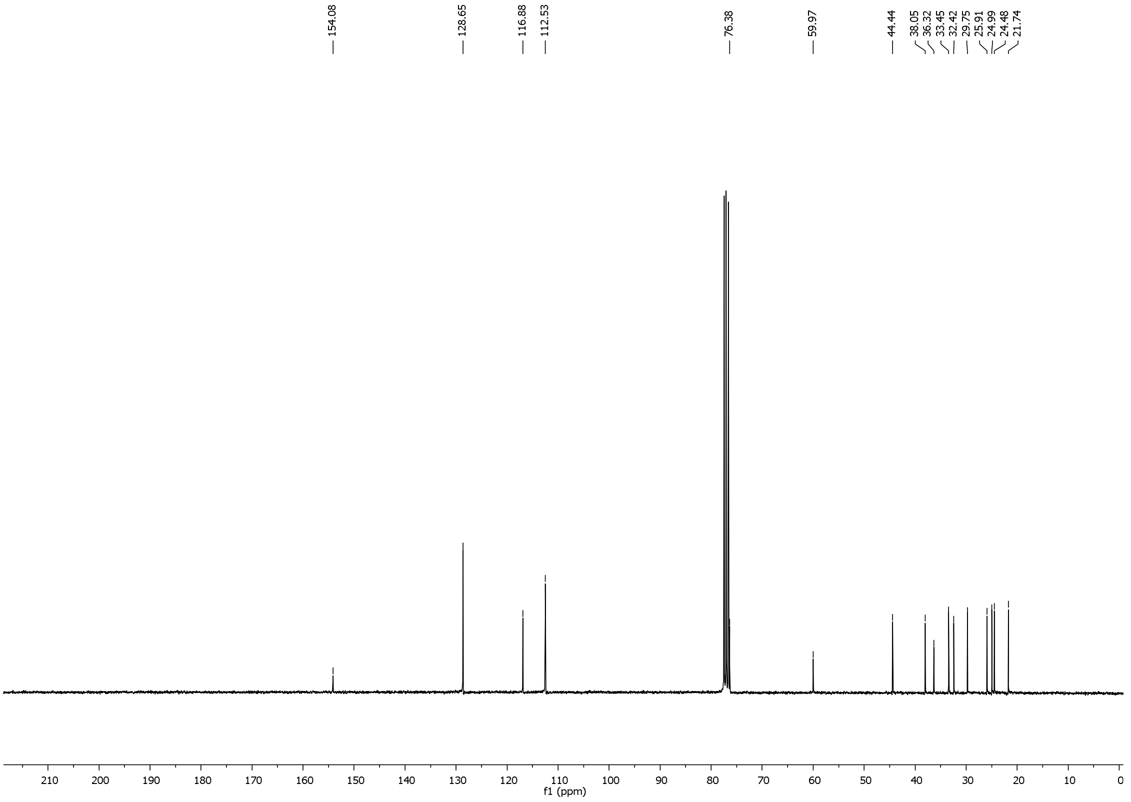

Figure SI-35. ^{13}C-NMR (75.5 MHz, $CDCl_3$) spectra of compound **2j**.

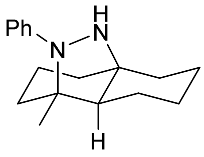

2k

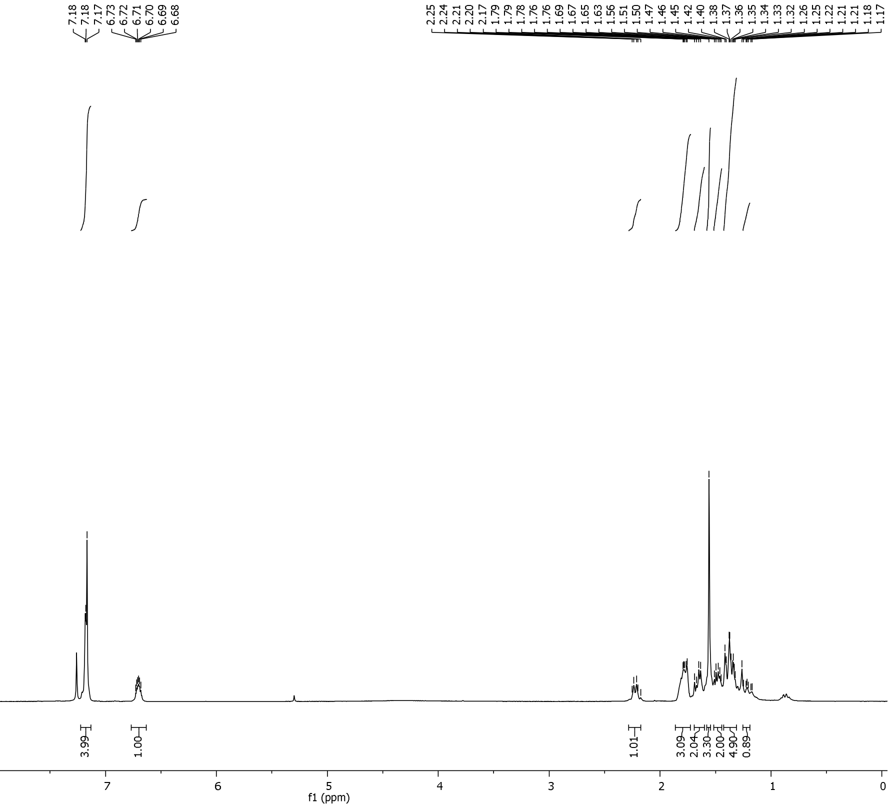

Figure SI-36. ^{1}H-NMR (300 MHz, $CDCl_3$) spectra of compound **2k**.

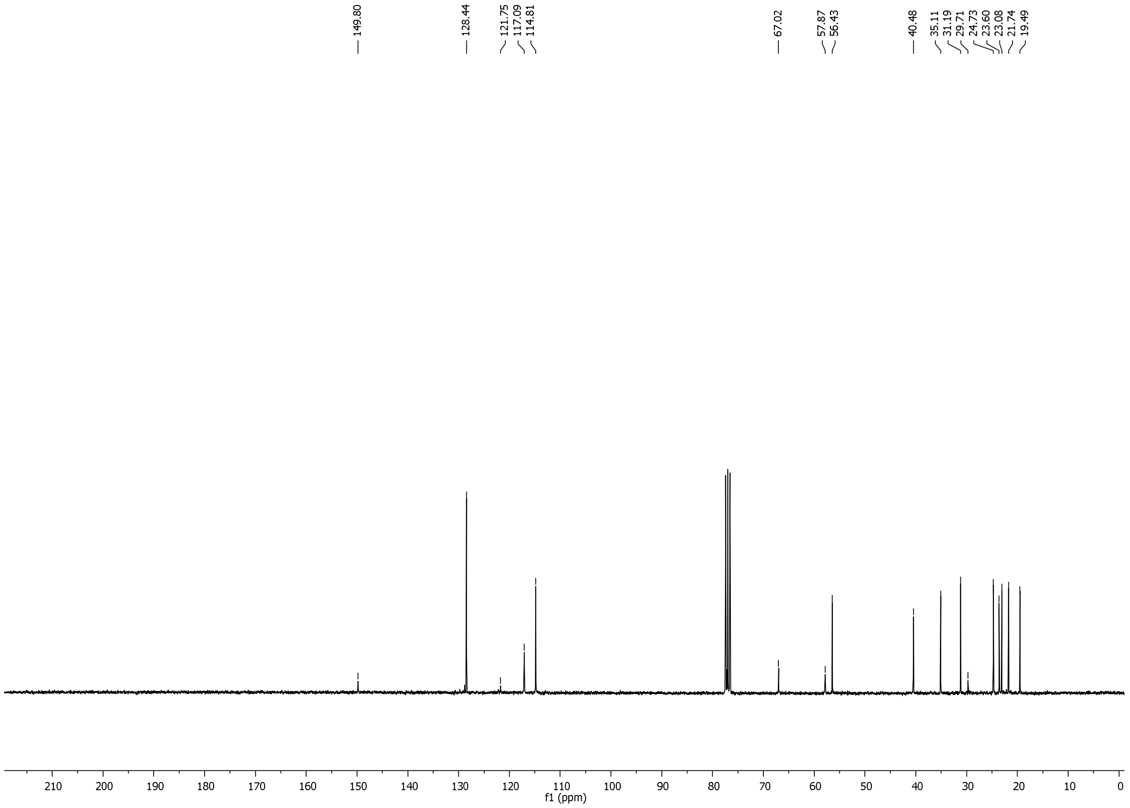

Figure SI-37. ^{13}C-NMR (75.5 MHz, $CDCl_3$) spectra of compound **2k**.

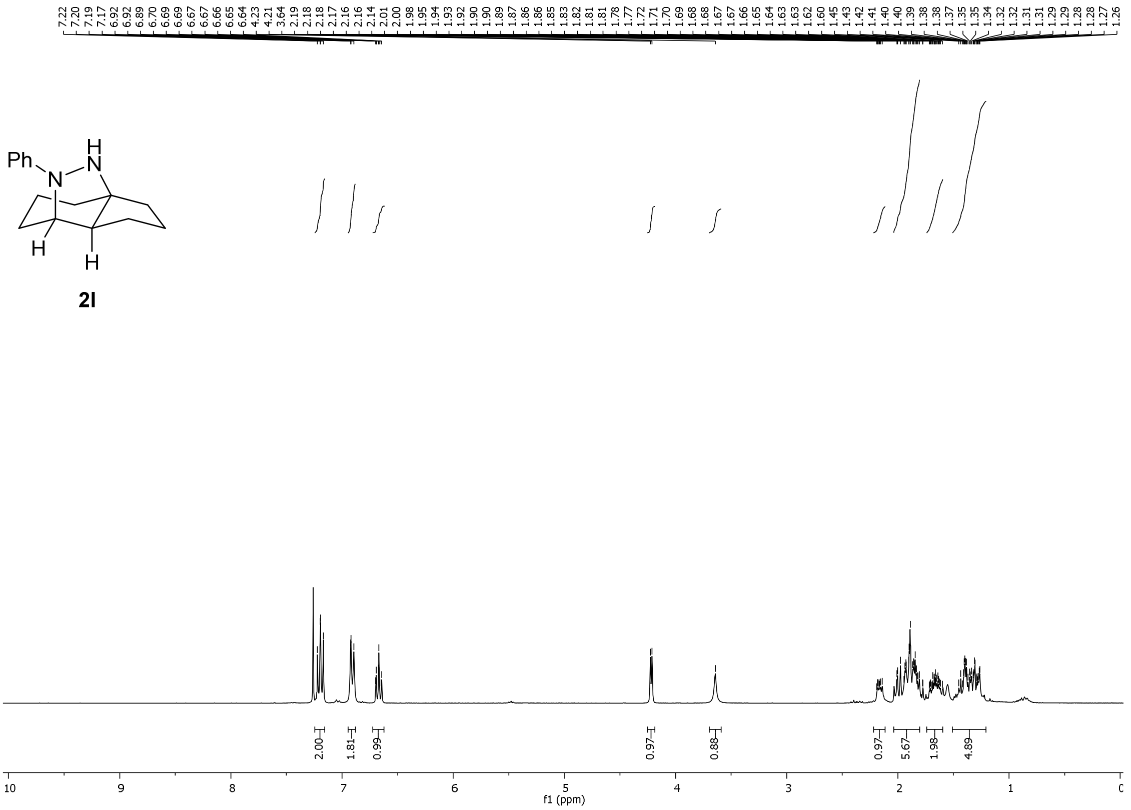

Figure SI-38. ^{1}H-NMR (300 MHz, $CDCl_3$) spectra of compound **2l**.

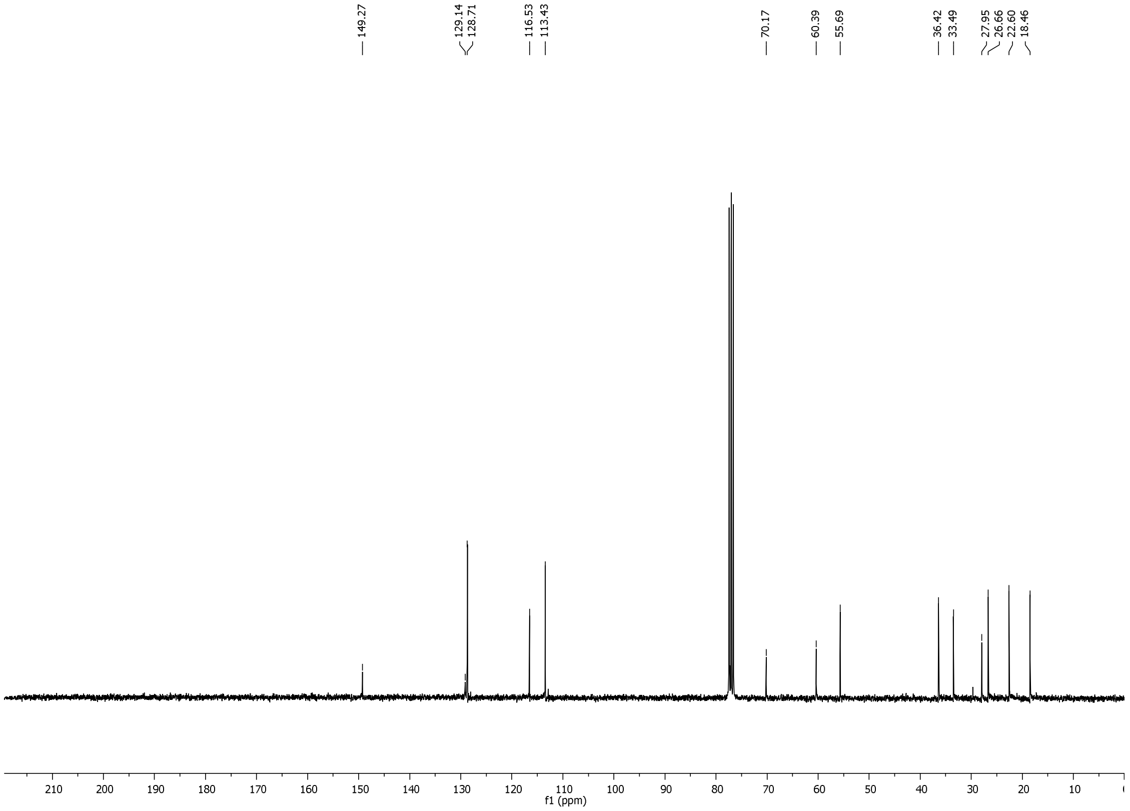

Figure SI-39. ^{13}C-NMR (75.5 MHz, $CDCl_3$) spectra of compound **2l**.

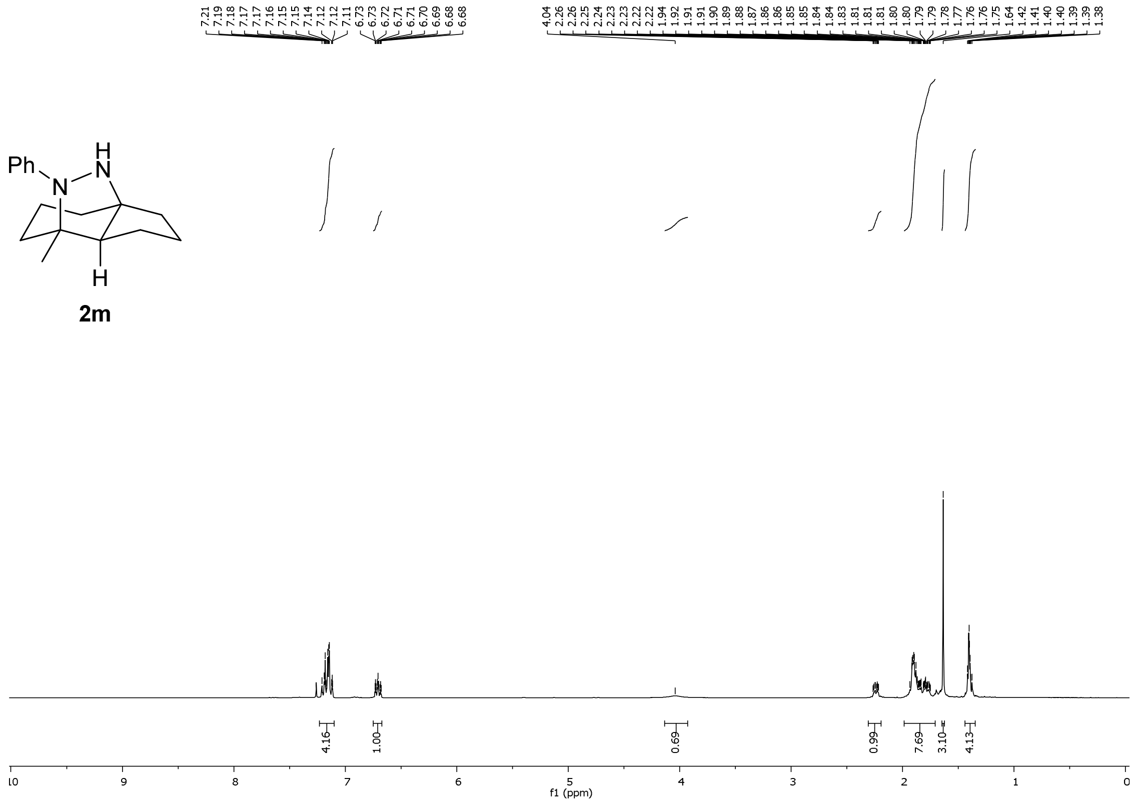

Figure SI-40. ^{1}H-NMR (300 MHz, $CDCl_3$) spectra of compound **2m**.

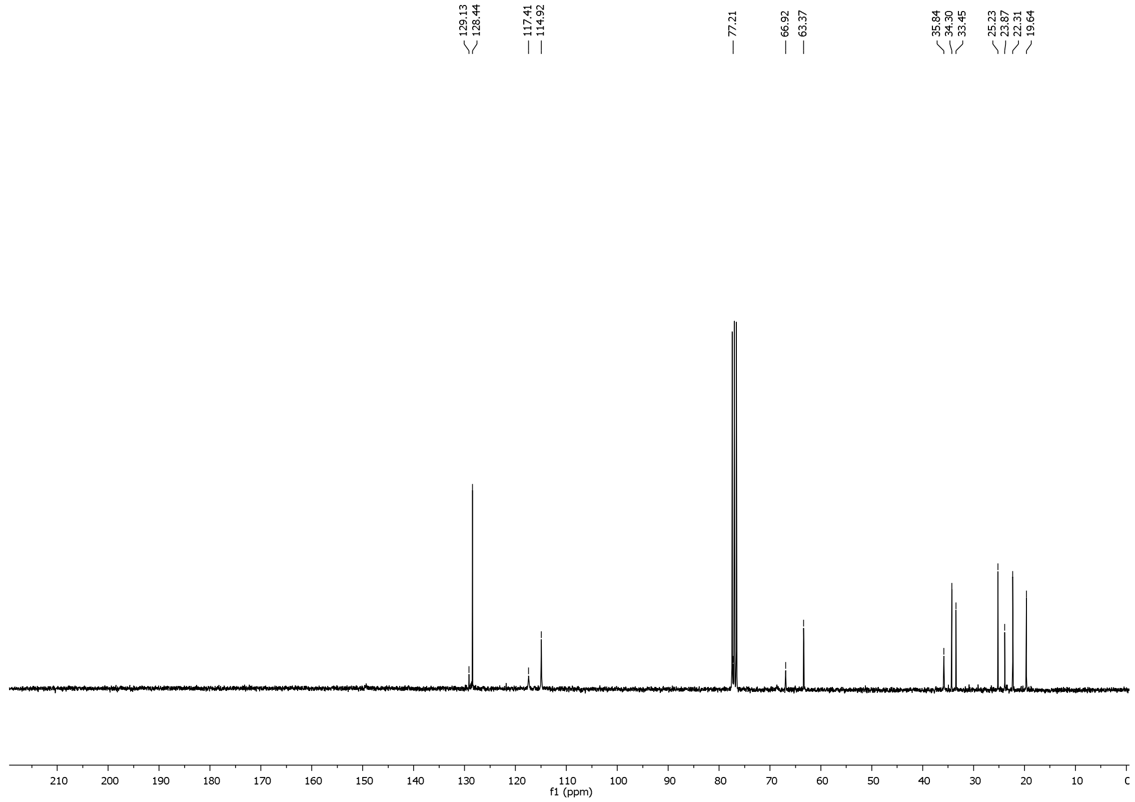

Figure SI-41. ^{13}C-NMR (75.5 MHz, $CDCl_3$) spectra of compound **2m**.

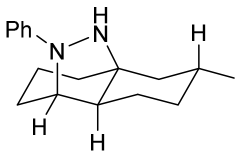

2n

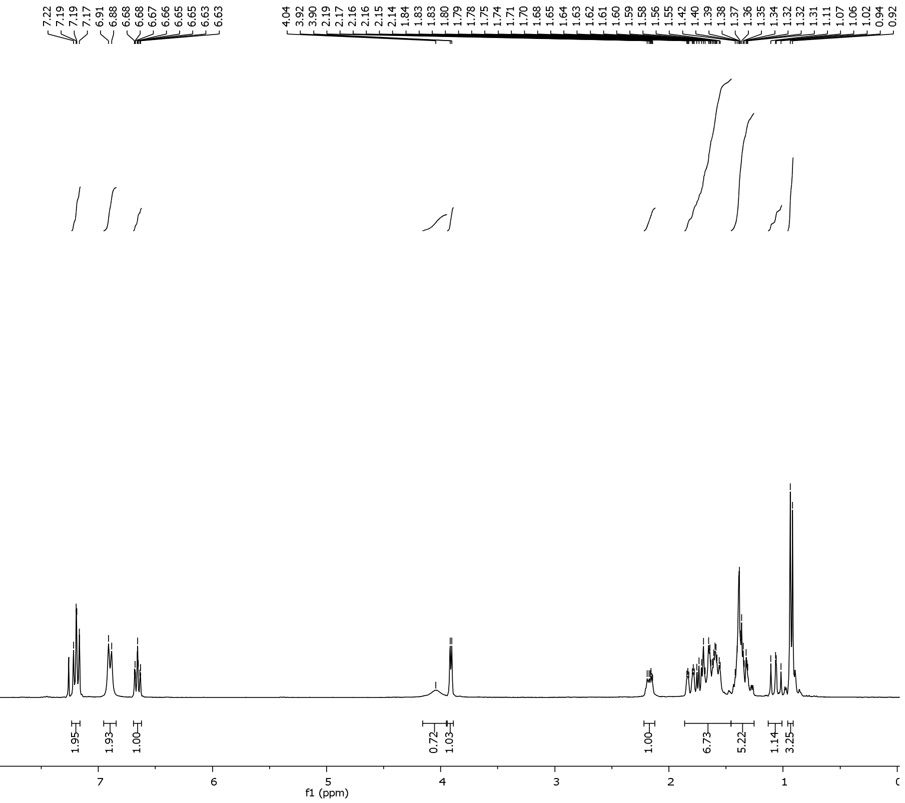

Figure SI-42. ^{1}H-NMR (300 MHz, $CDCl_3$) spectra of compound **2n**.

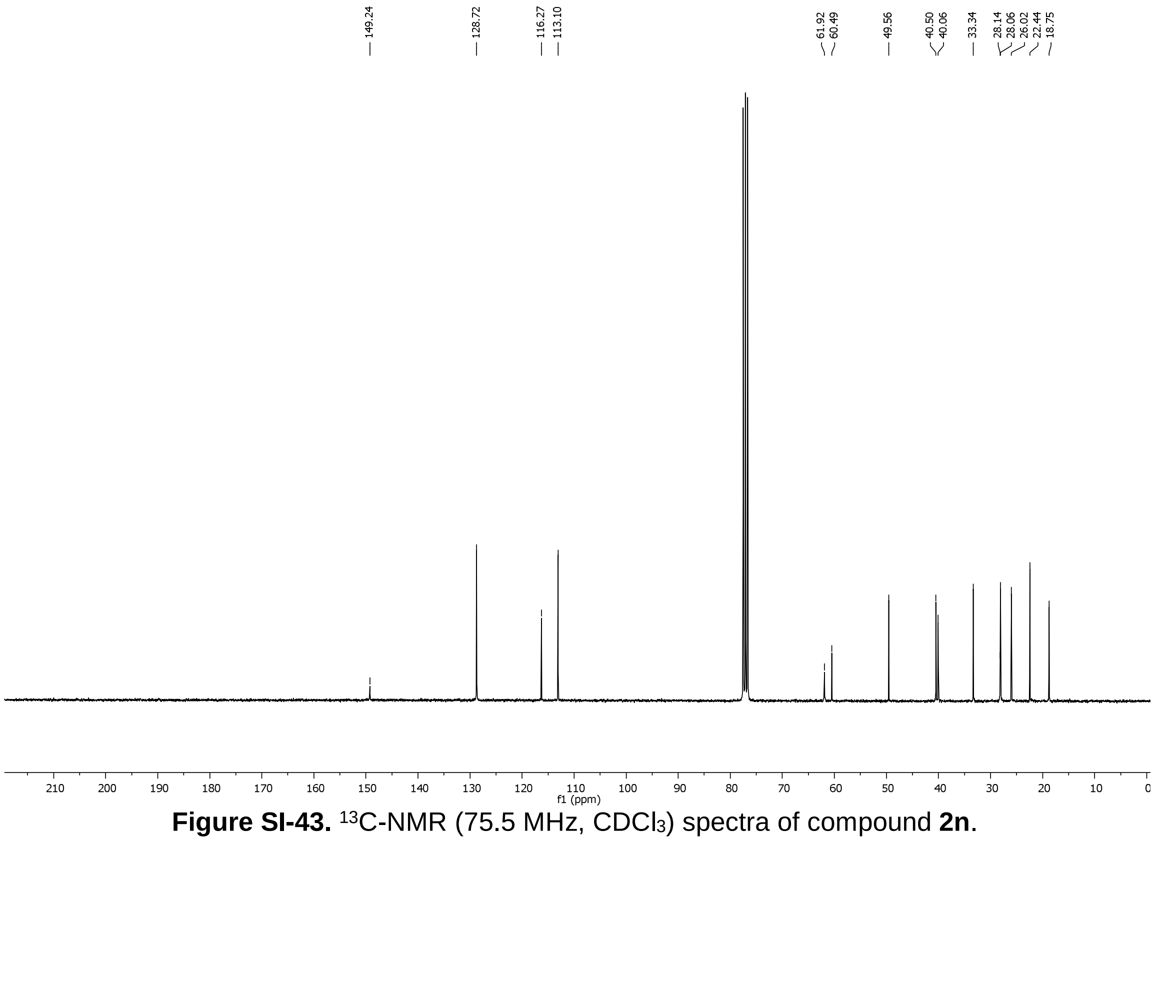

Figure SI-43. ^{13}C-NMR (75.5 MHz, $CDCl_3$) spectra of compound **2n**.

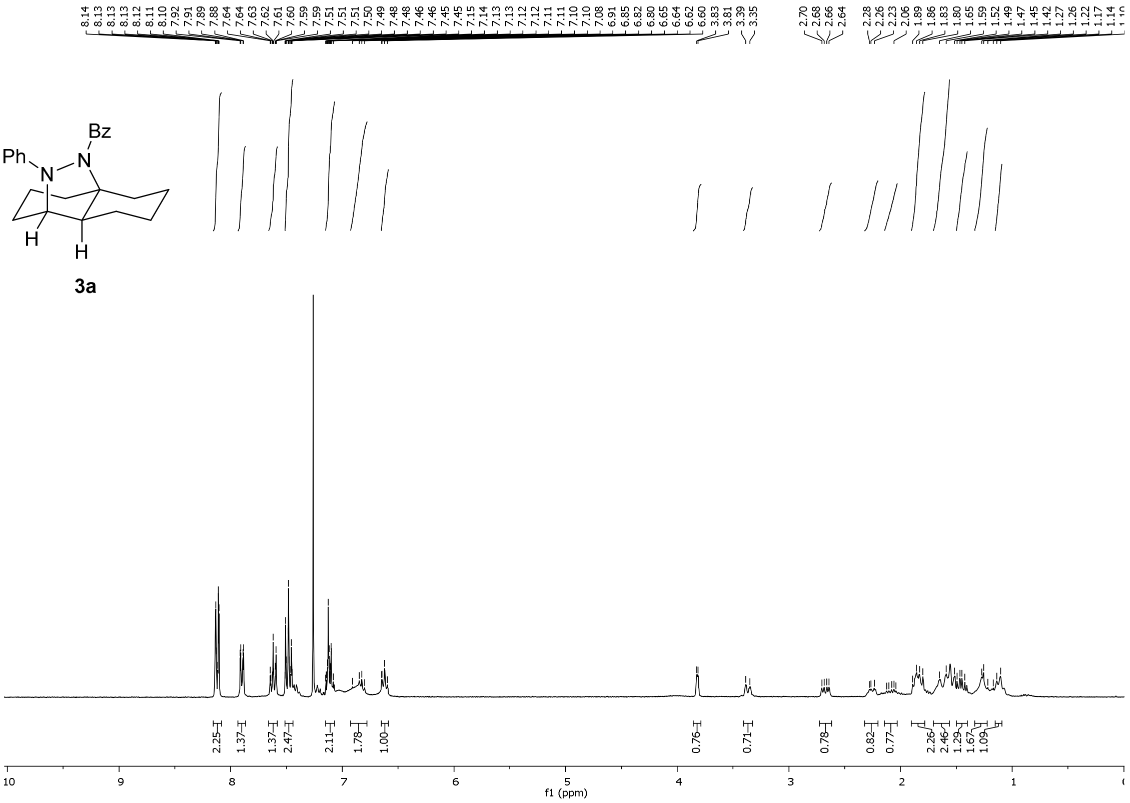

Figure SI-44. ^{1}H-NMR (300 MHz, $CDCl_3$) spectra of compound **3a**.

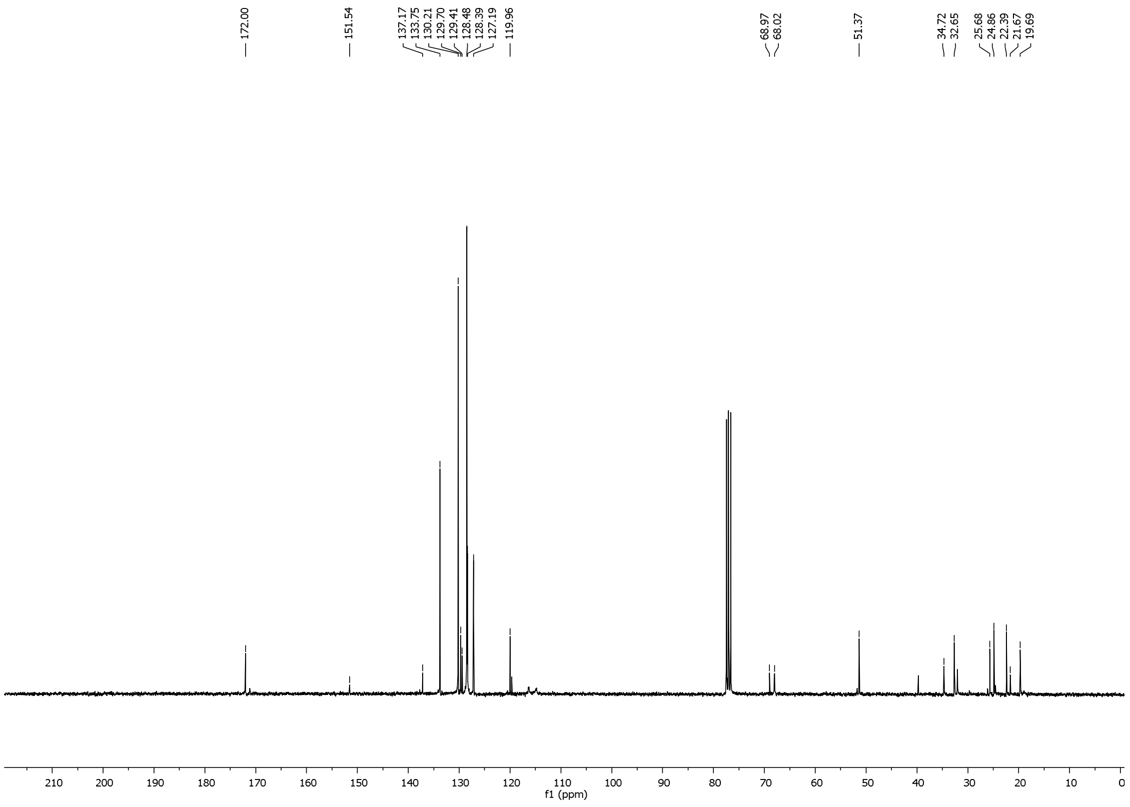

Figure SI-45. ^{13}C-NMR (75.5 MHz, $CDCl_3$) spectra of compound **3a**.

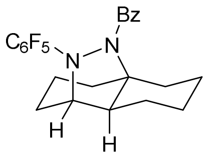

3b

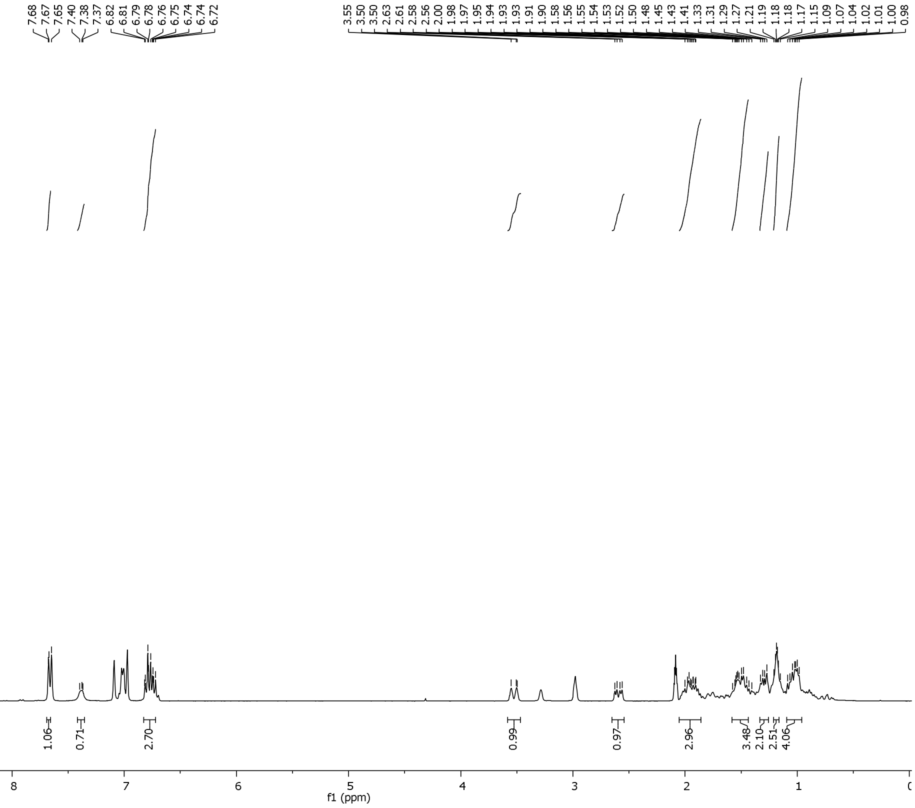

Figure SI-46. ^{1}H-NMR (300 MHz, $CDCl_3$) spectra of compound **3b**.

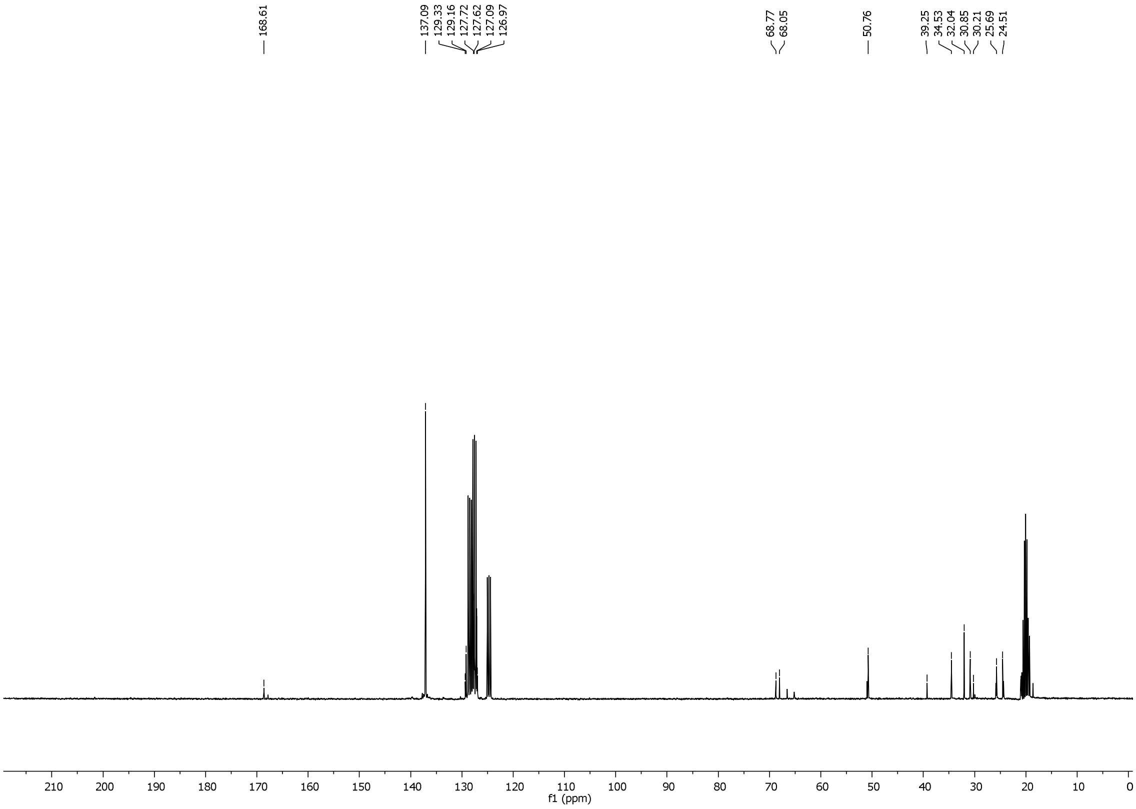

Figure SI-47. ^{13}C-NMR (75.5 MHz, $CDCl_3$) spectra of compound **3b**.

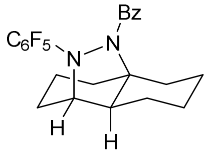

3b

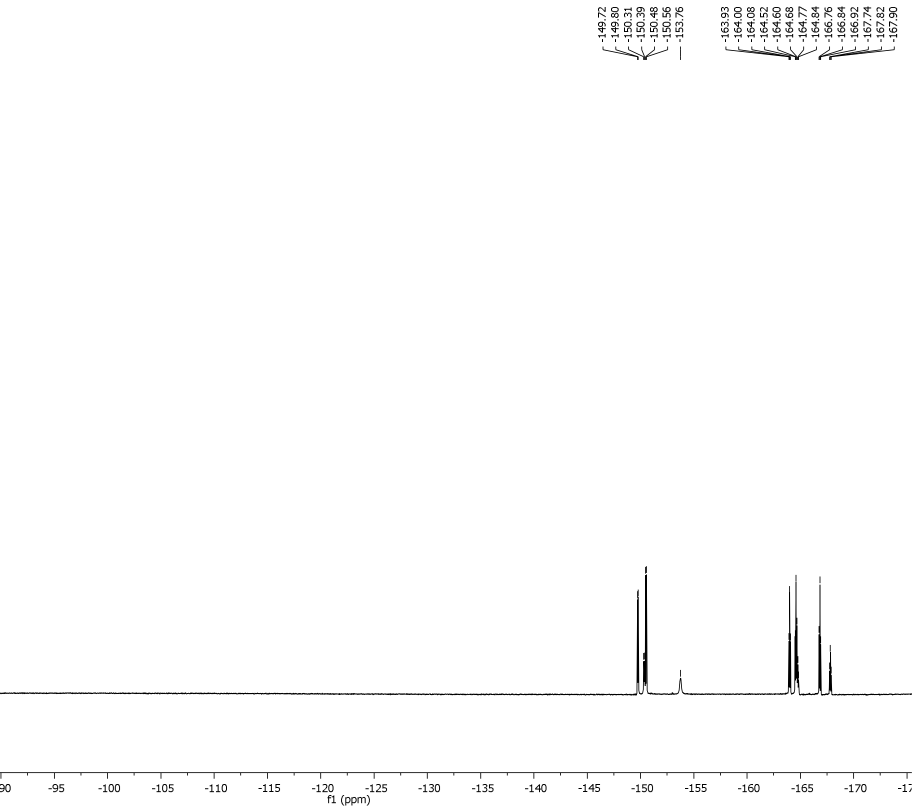

Figure SI-48. ^{19}F-NMR (282 MHz, $CDCl_3$) spectra of compound **3b**.

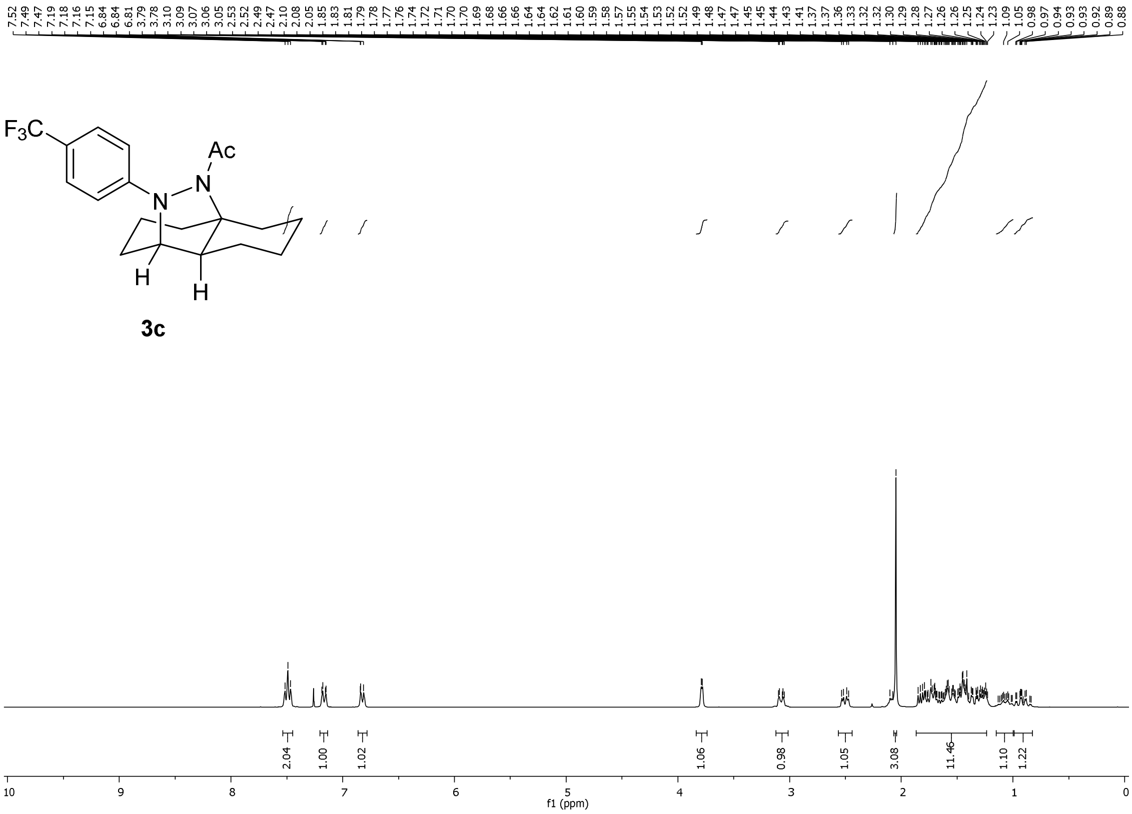

Figure SI-49. ^{1}H-NMR (300 MHz, $CDCl_3$) spectra of compound **3c**.

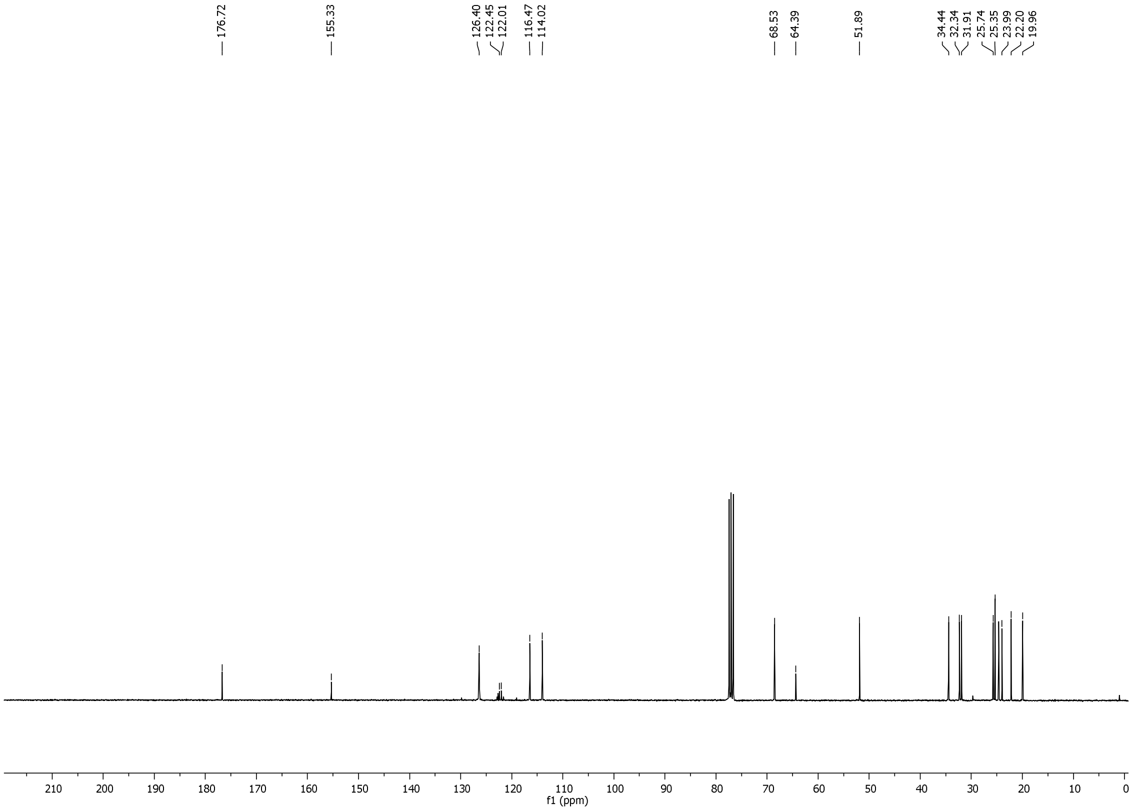

Figure SI-50. ^{13}C-NMR (75.5 MHz, $CDCl_3$) spectra of compound **3c**.

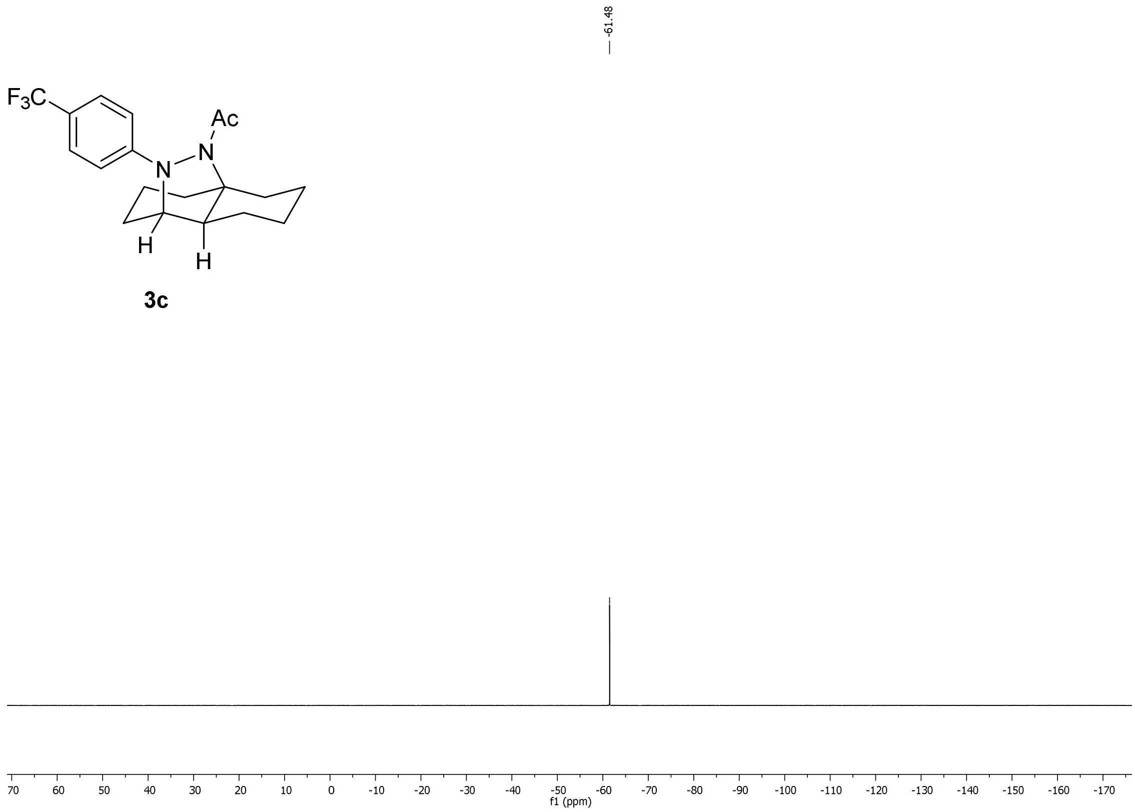

Figure SI-51. ^{19}F-NMR (282 MHz, $CDCl_3$) spectra of compound **3c**.

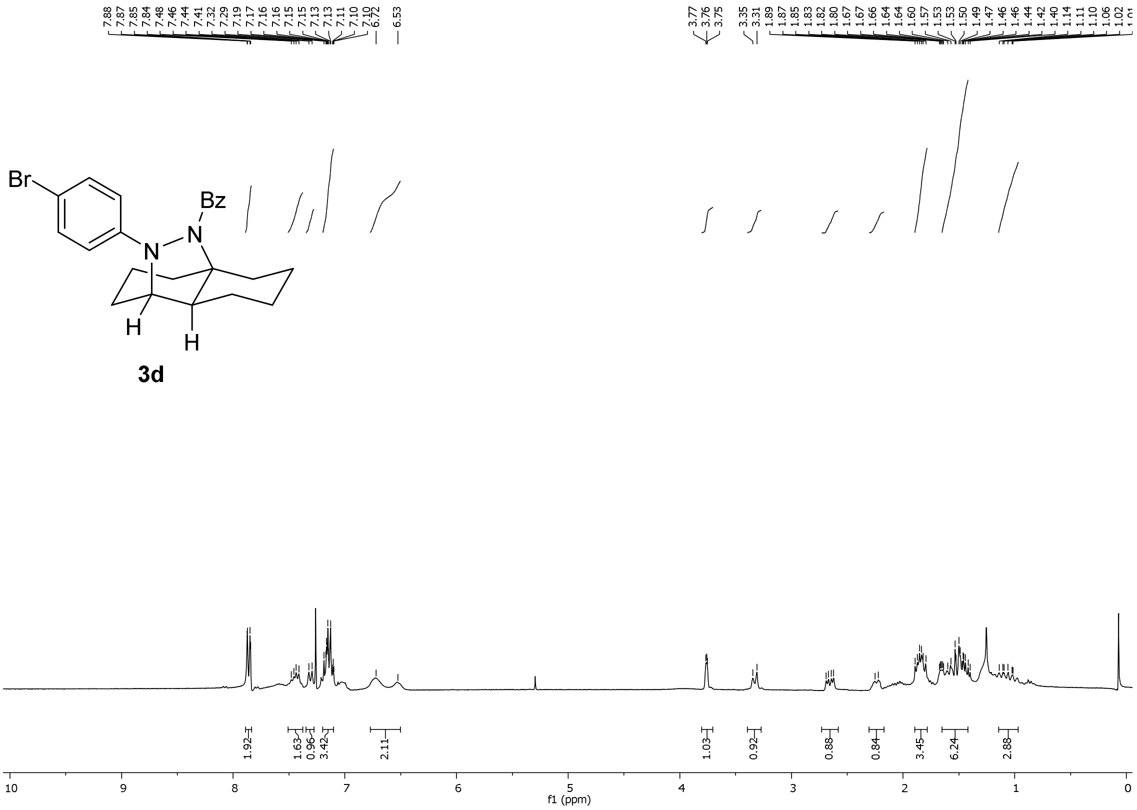

Figure SI-52. ^{1}H-NMR (300 MHz, $CDCl_3$) spectra of compound **3d**.

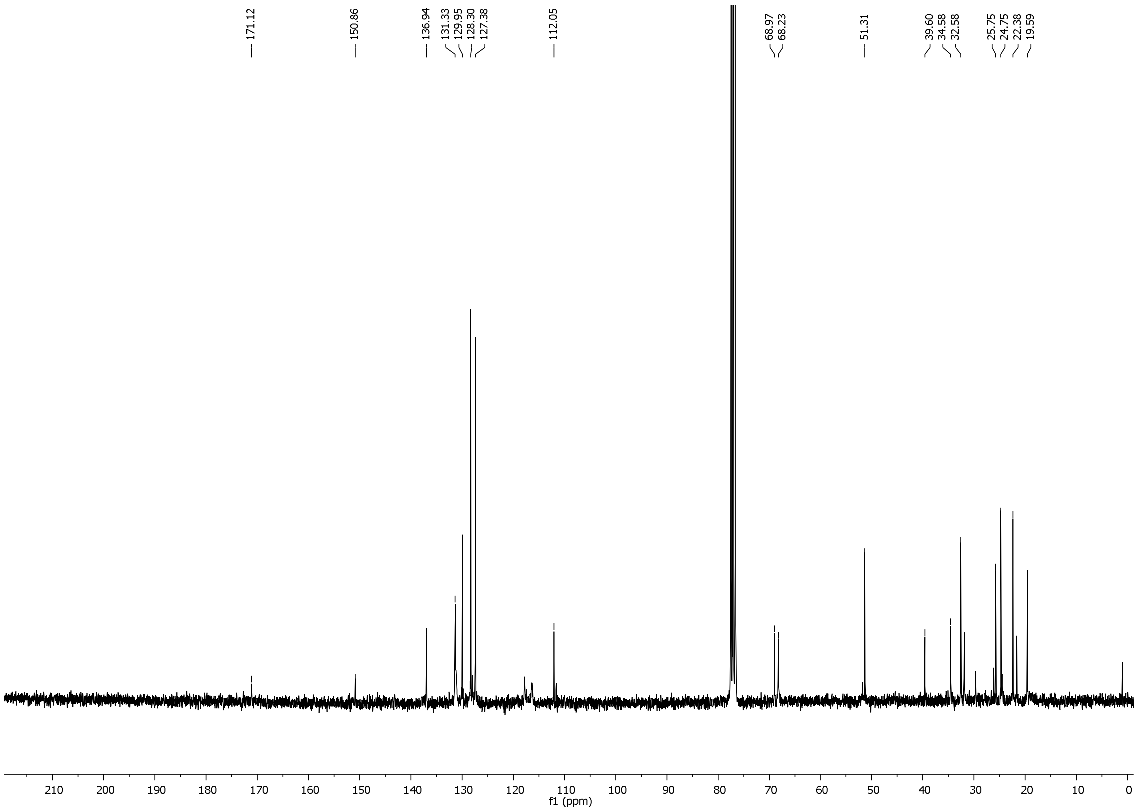

Figure SI-53. ^{13}C-NMR (75.5 MHz, $CDCl_3$) spectra of compound **3d**.

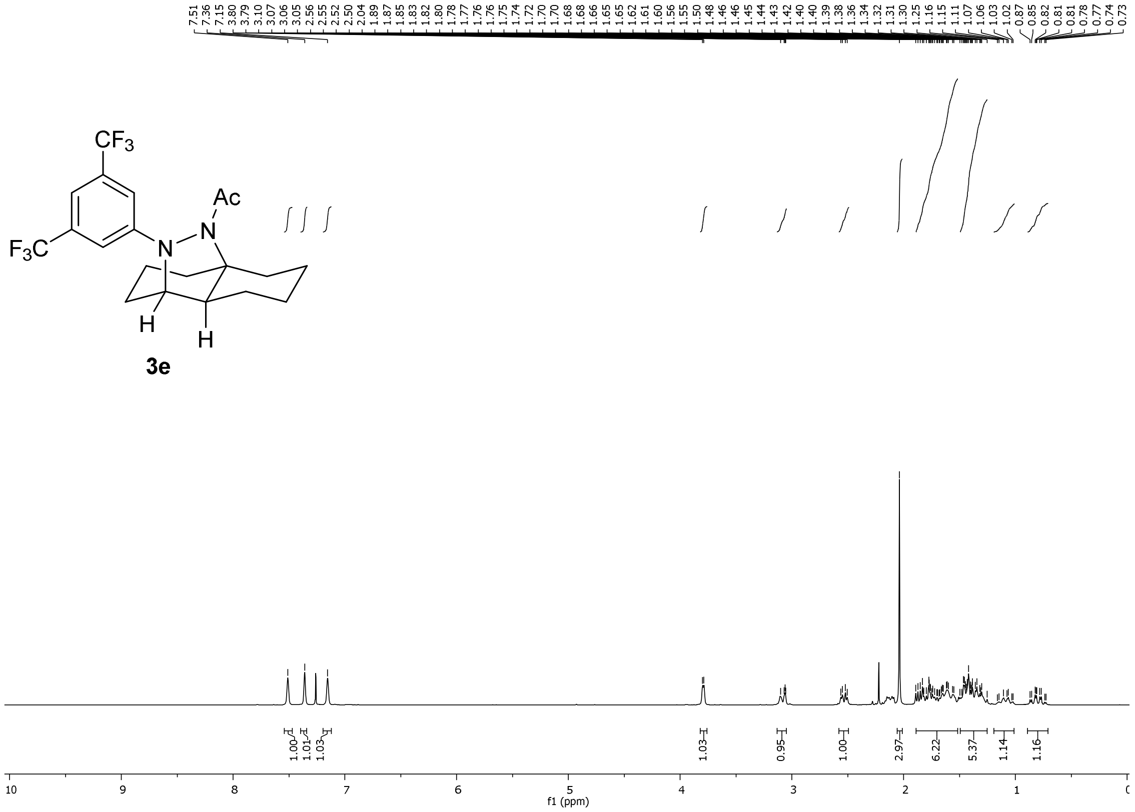

Figure SI-54. ^{1}H-NMR (300 MHz, $CDCl_3$) spectra of compound **3e**.

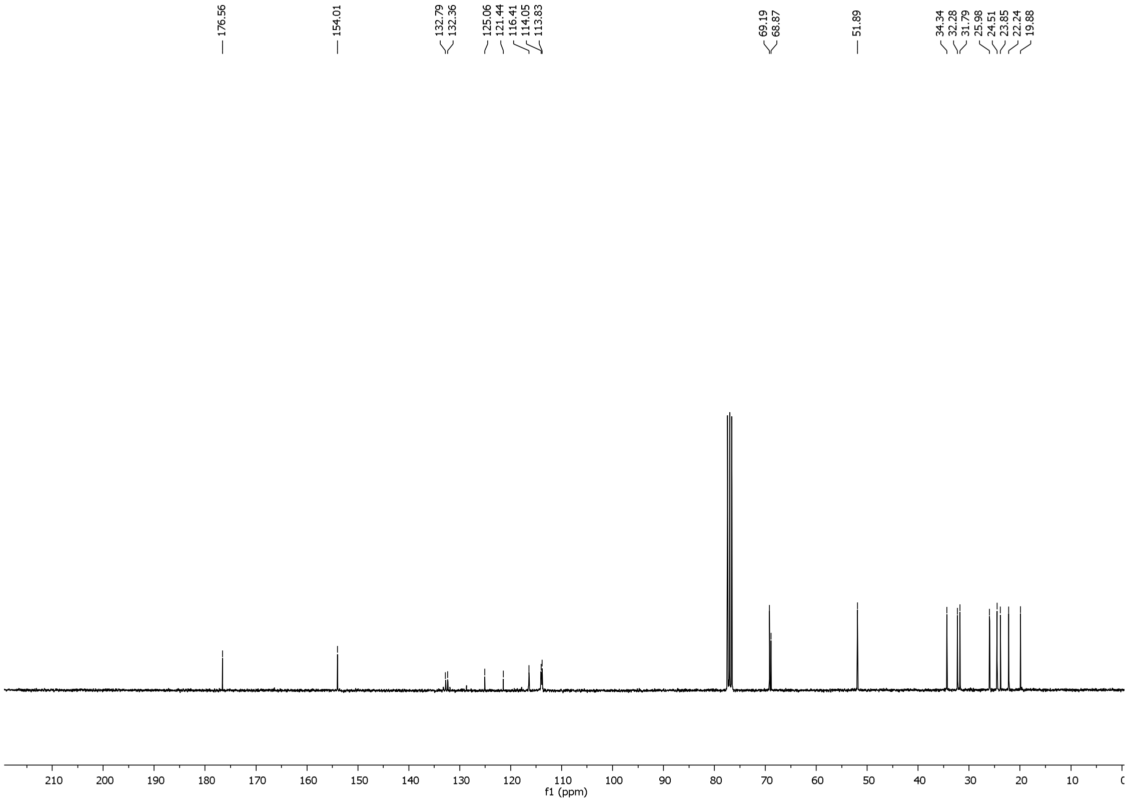

Figure SI-55. ^{13}C-NMR (75.5 MHz, $CDCl_3$) spectra of compound **3e**.

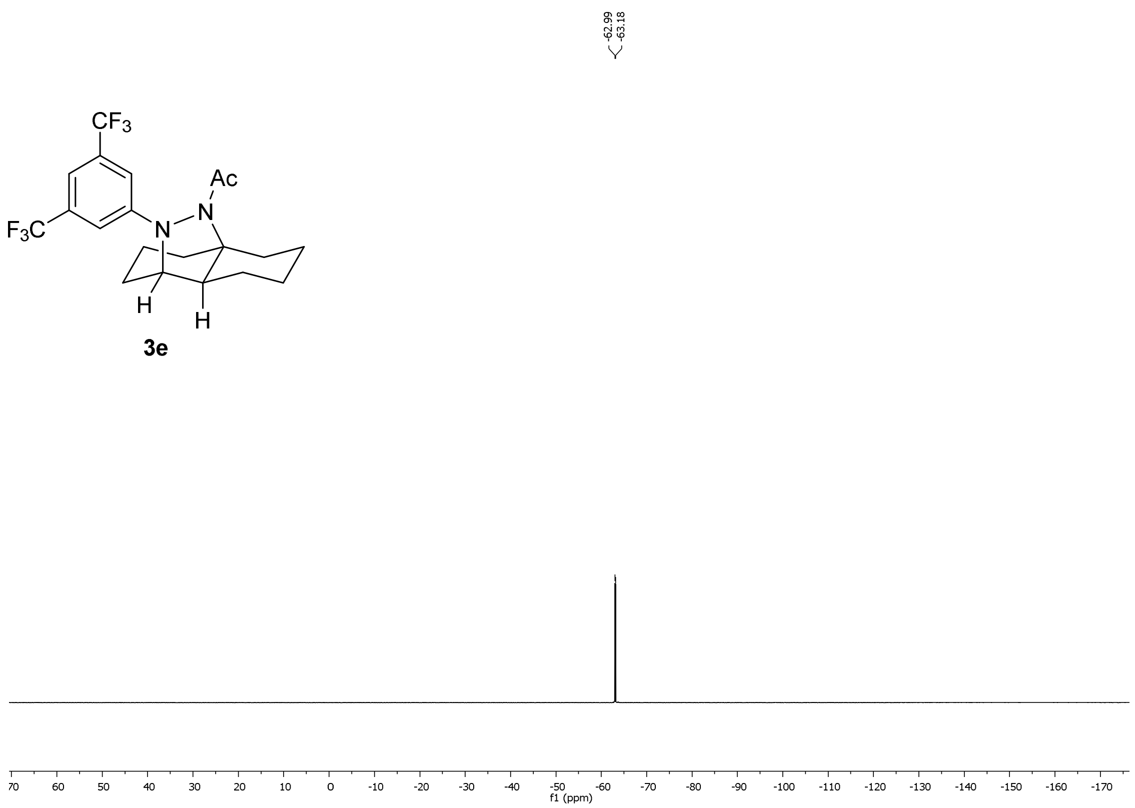

Figure SI-56. ^{19}F-NMR (282 MHz, $CDCl_3$) spectra of compound **3e**.

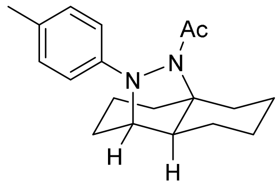

3f

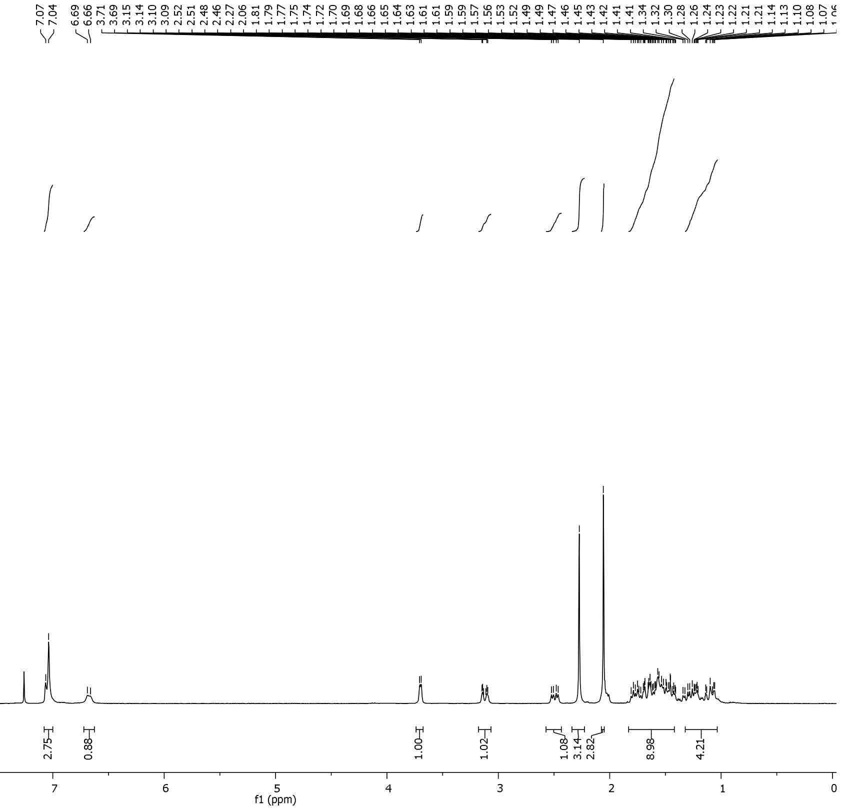

Figure SI-57. ^{1}H-NMR (300 MHz, $CDCl_3$) spectra of compound **3f**.

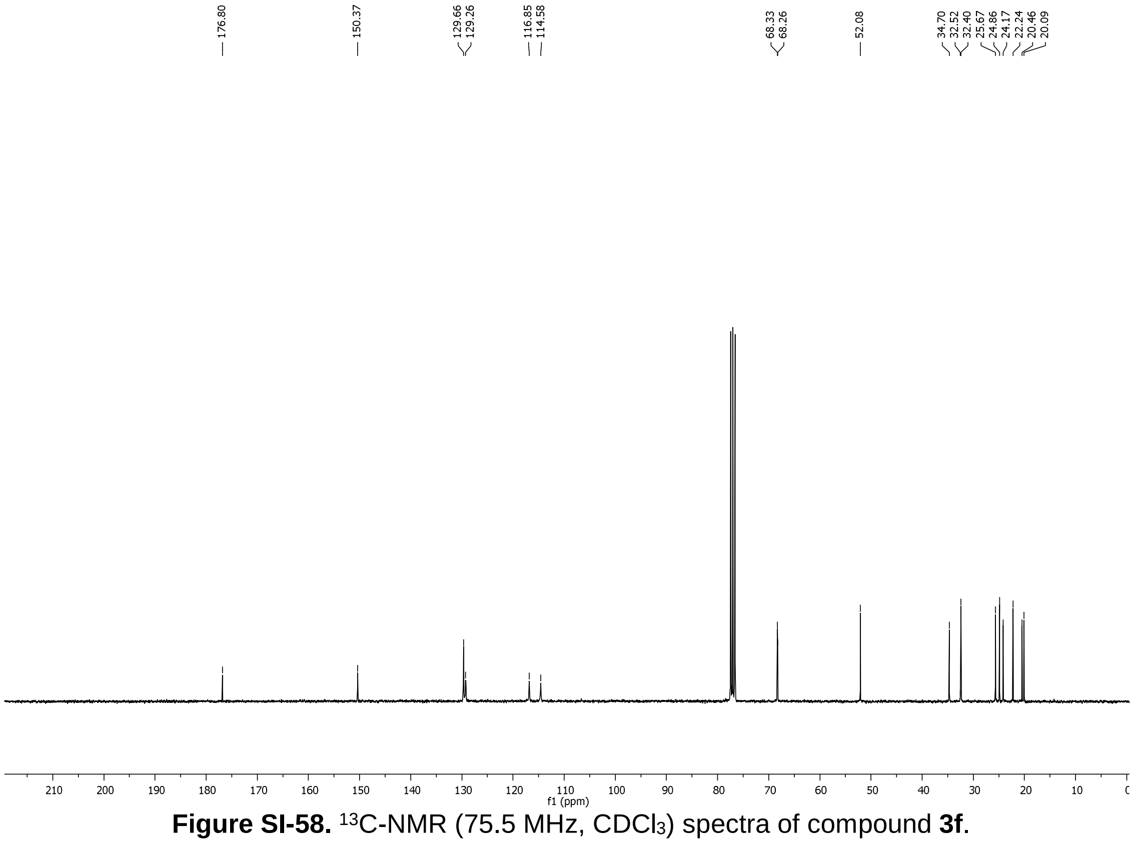

Figure SI-58. ^{13}C-NMR (75.5 MHz, $CDCl_3$) spectra of compound **3f**.

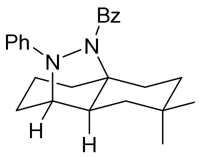

3i

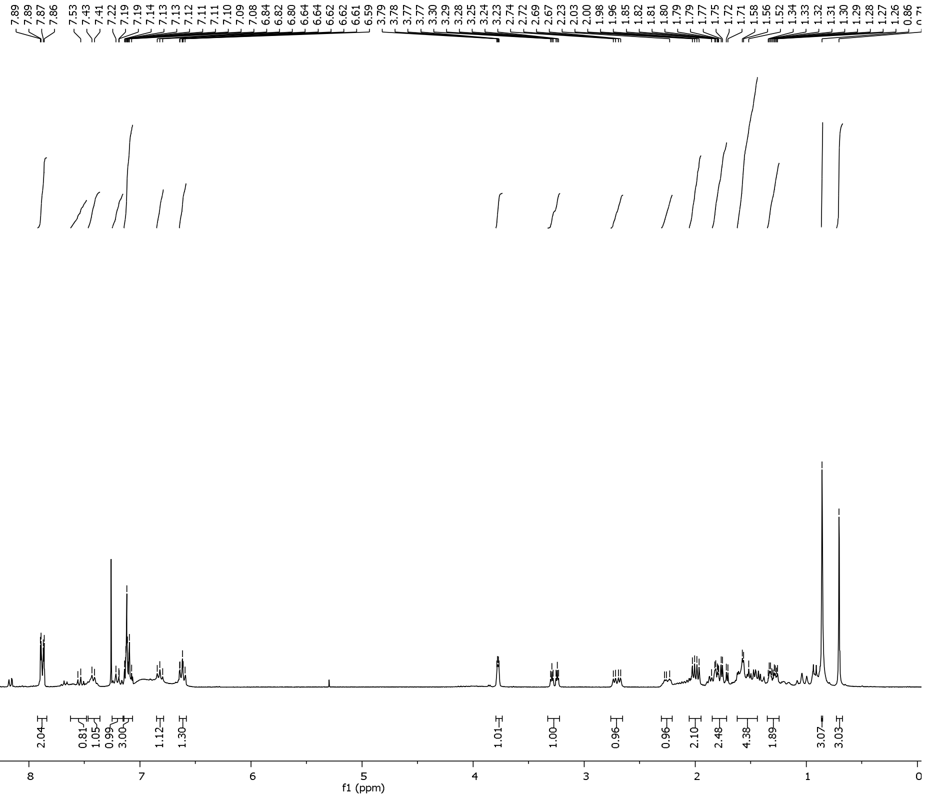

Figure SI-59. ^{1}H-NMR (300 MHz, $CDCl_3$) spectra of compound **3i**.

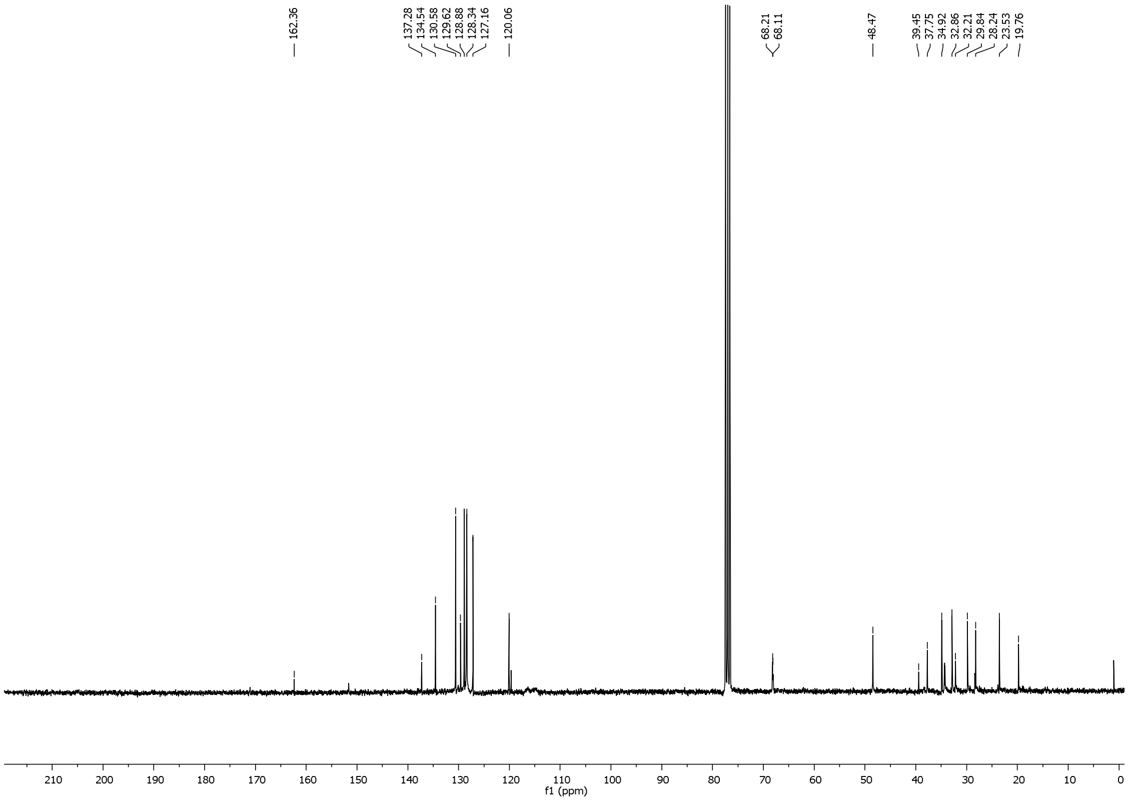

Figure SI-60. ^{13}C-NMR (75.5 MHz, $CDCl_3$) spectra of compound **3i**.

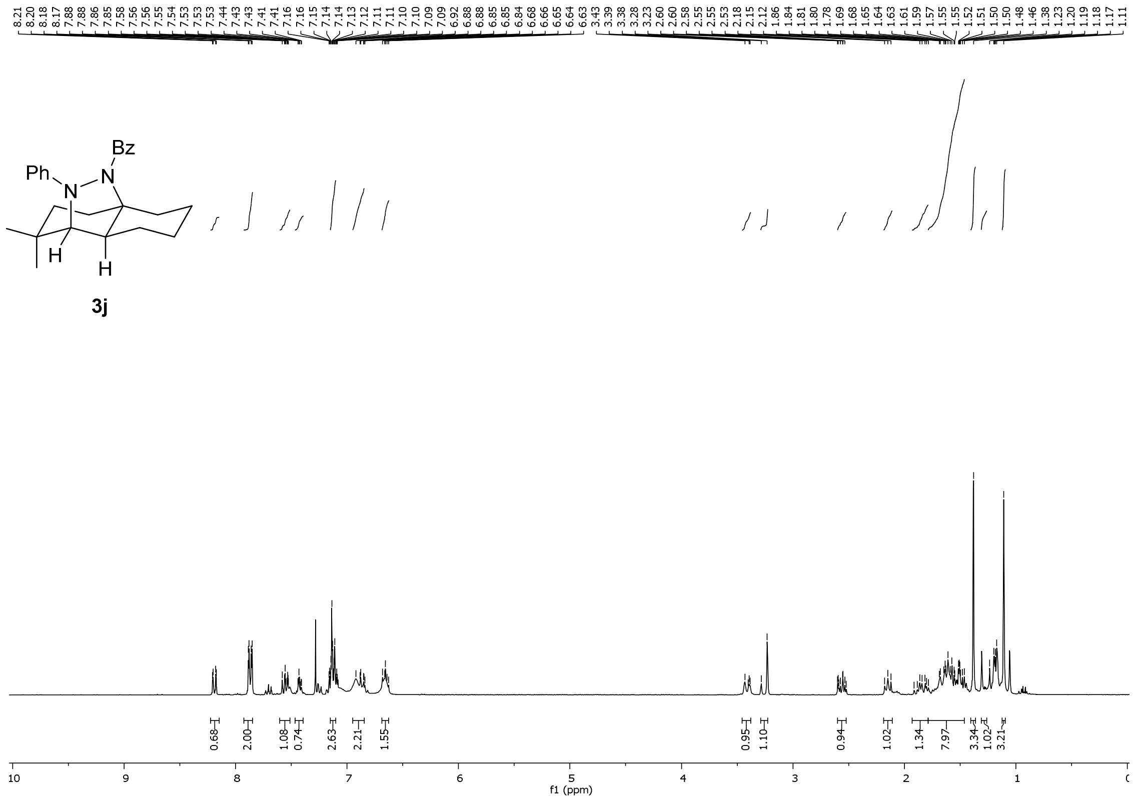

Figure SI-61. ^{1}H-NMR (300 MHz, $CDCl_3$) spectra of compound **3j**.

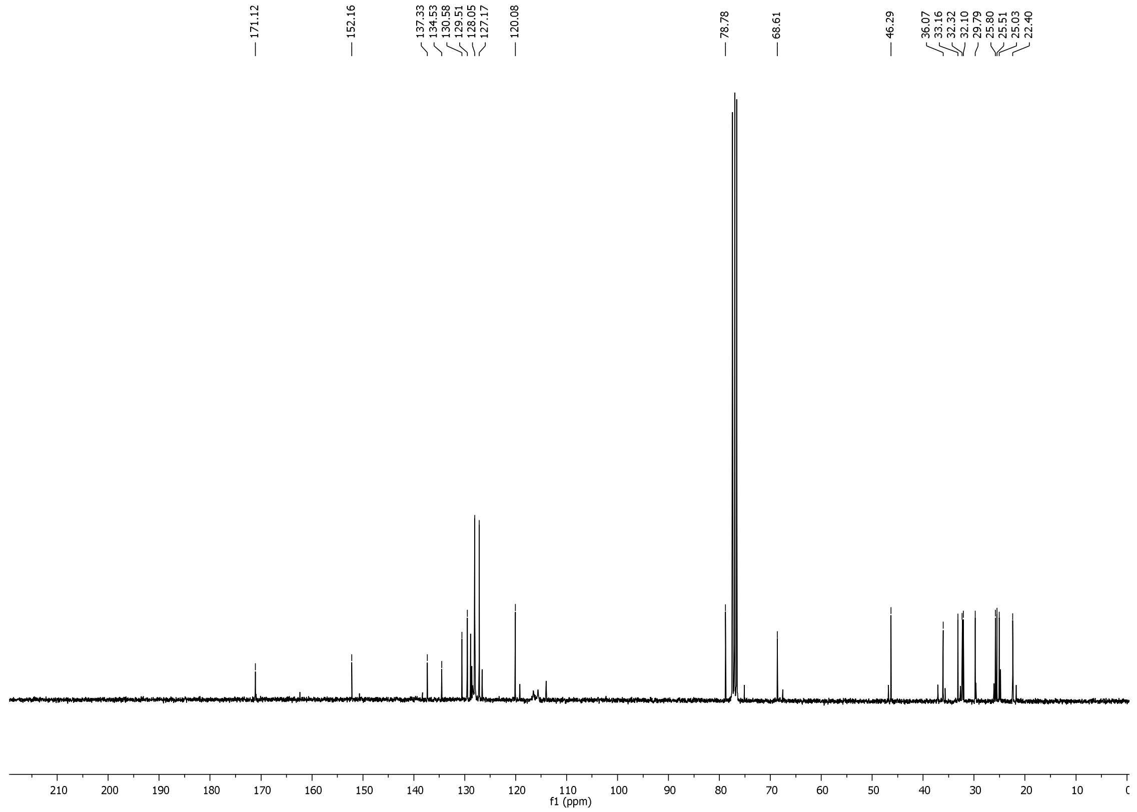

Figure SI-62. ^{13}C-NMR (75.5 MHz, $CDCl_3$) spectra of compound **3j**.

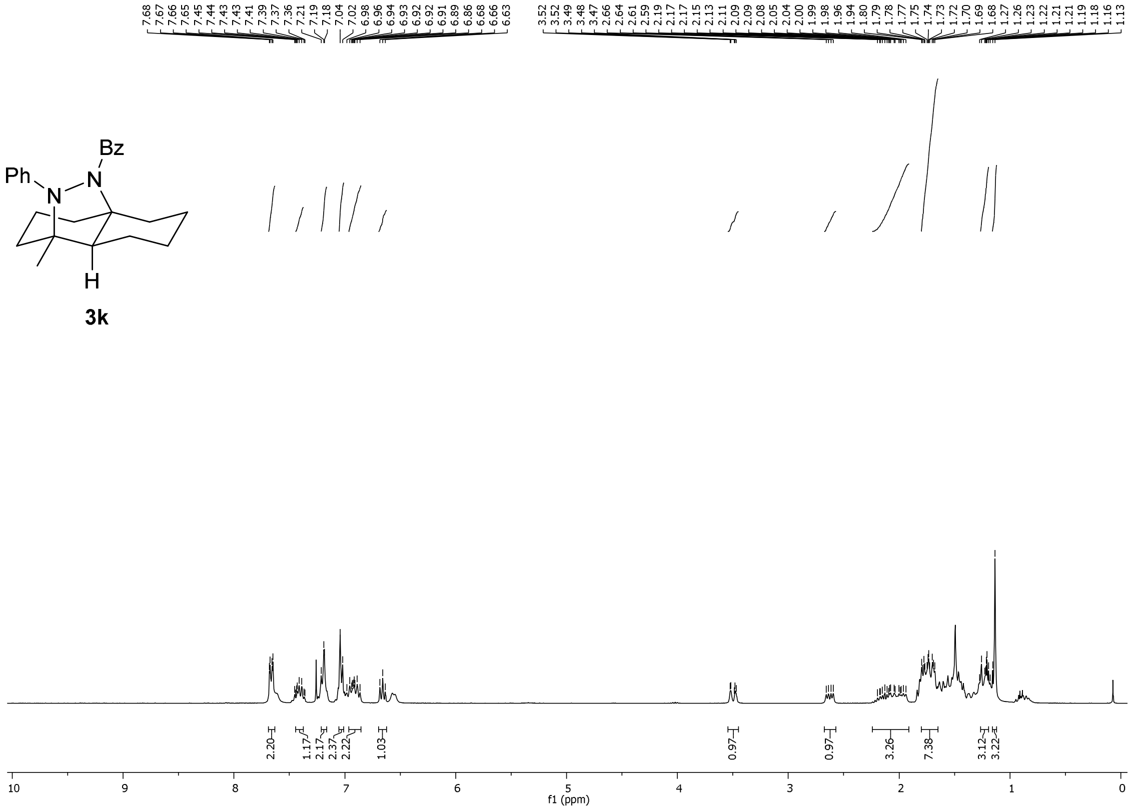

Figure SI-63. ^{1}H-NMR (300 MHz, $CDCl_3$) spectra of compound **3k**.

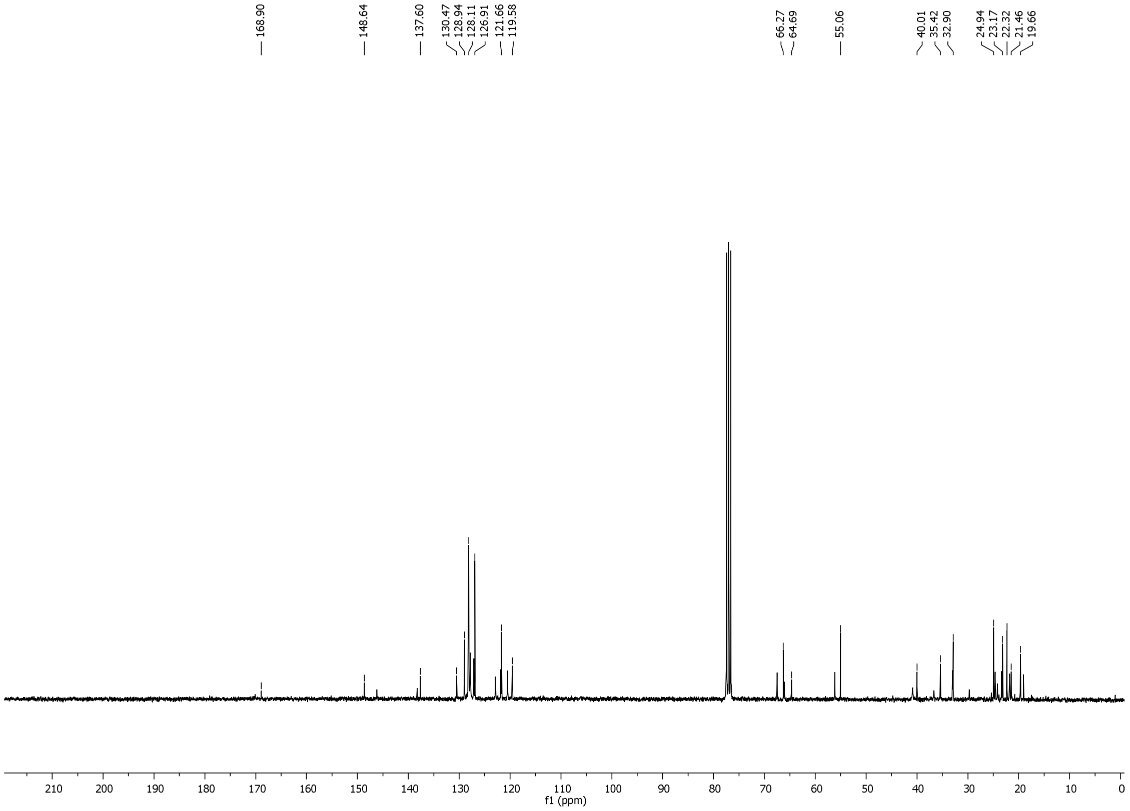

Figure SI-64. ^{13}C-NMR (75.5 MHz, $CDCl_3$) spectra of compound **3k**.

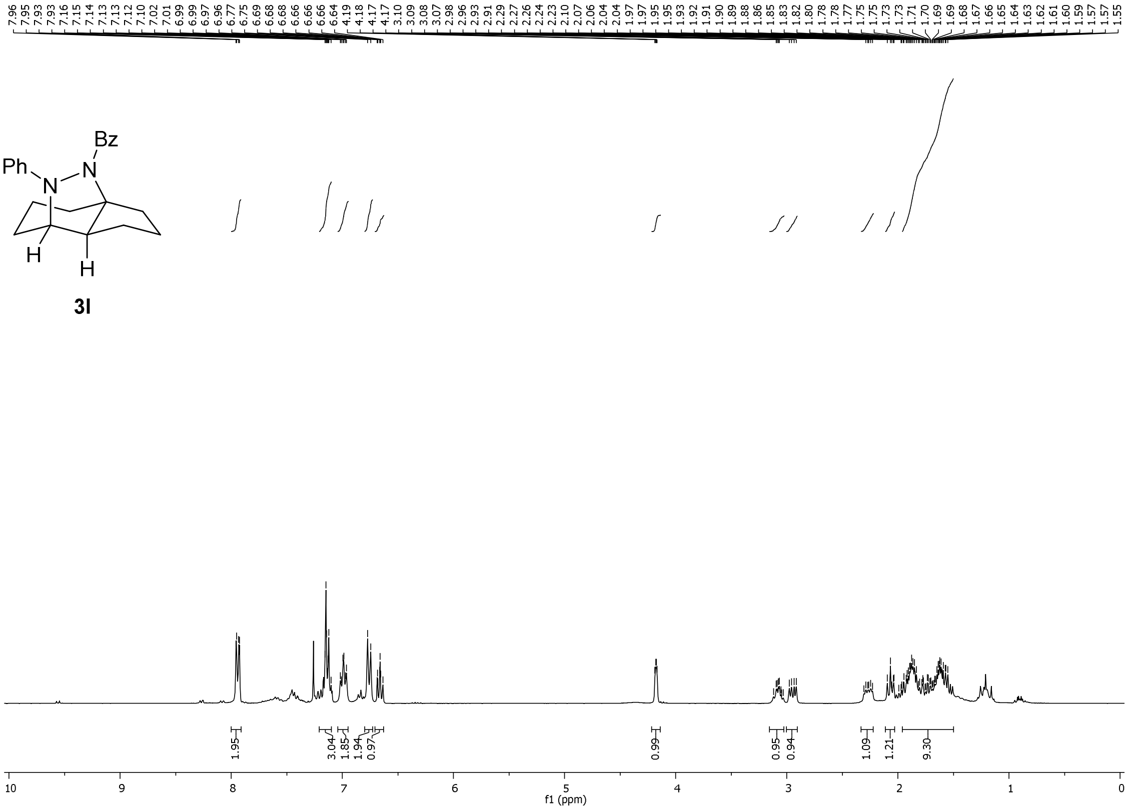

Figure SI-65. ^{1}H-NMR (300 MHz, $CDCl_3$) spectra of compound **3l**.

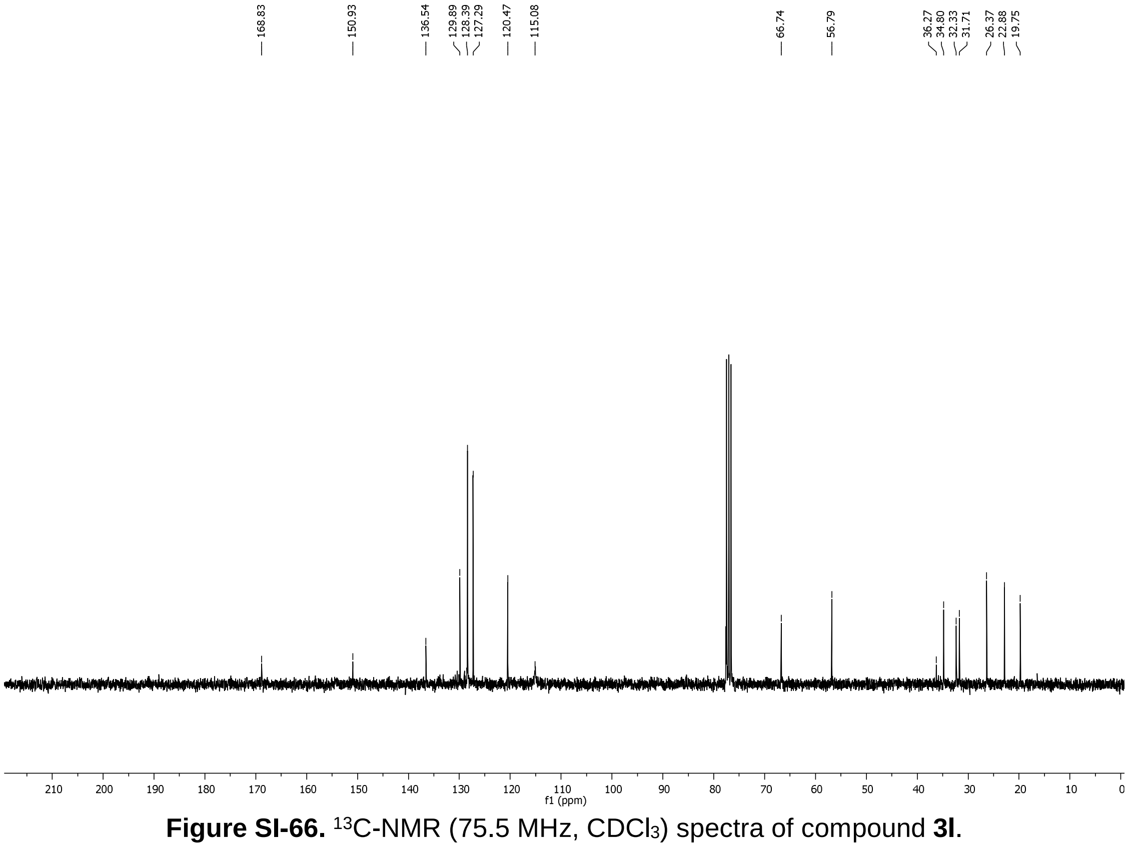

Figure SI-66. ^{13}C-NMR (75.5 MHz, $CDCl_3$) spectra of compound **3l**.

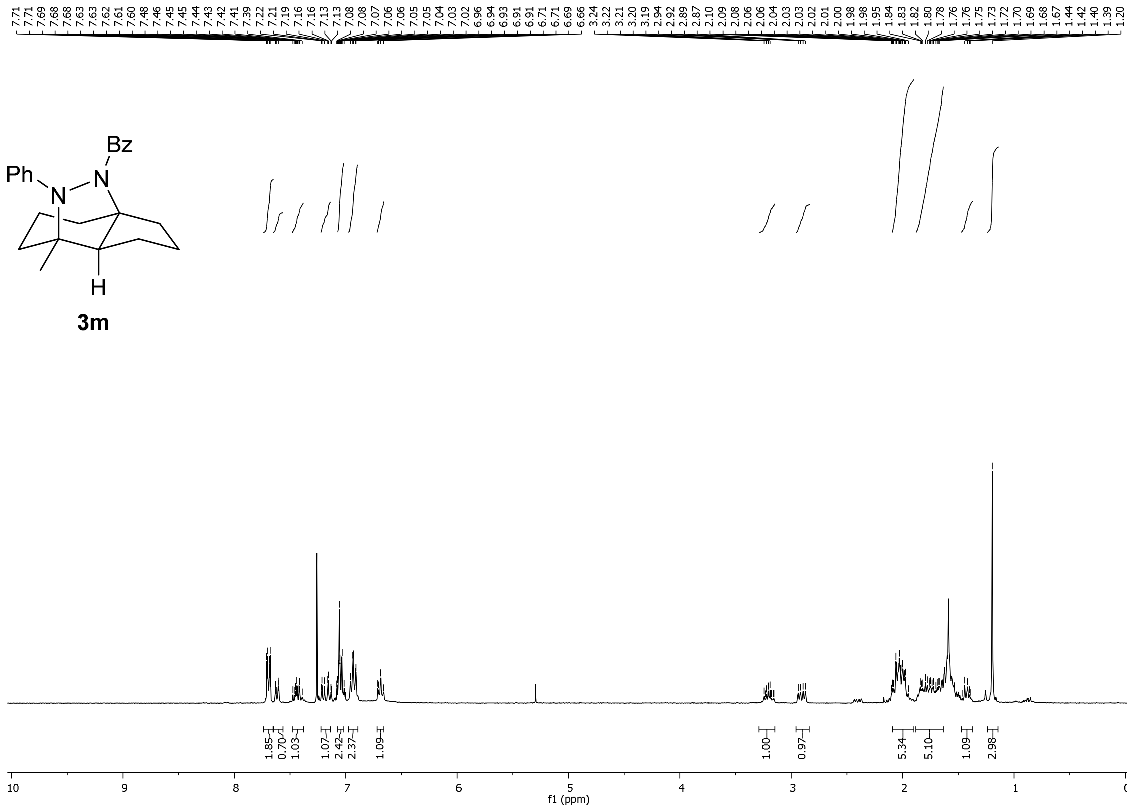

Figure SI-67. ^{1}H-NMR (300 MHz, $CDCl_3$) spectra of compound **3m**.

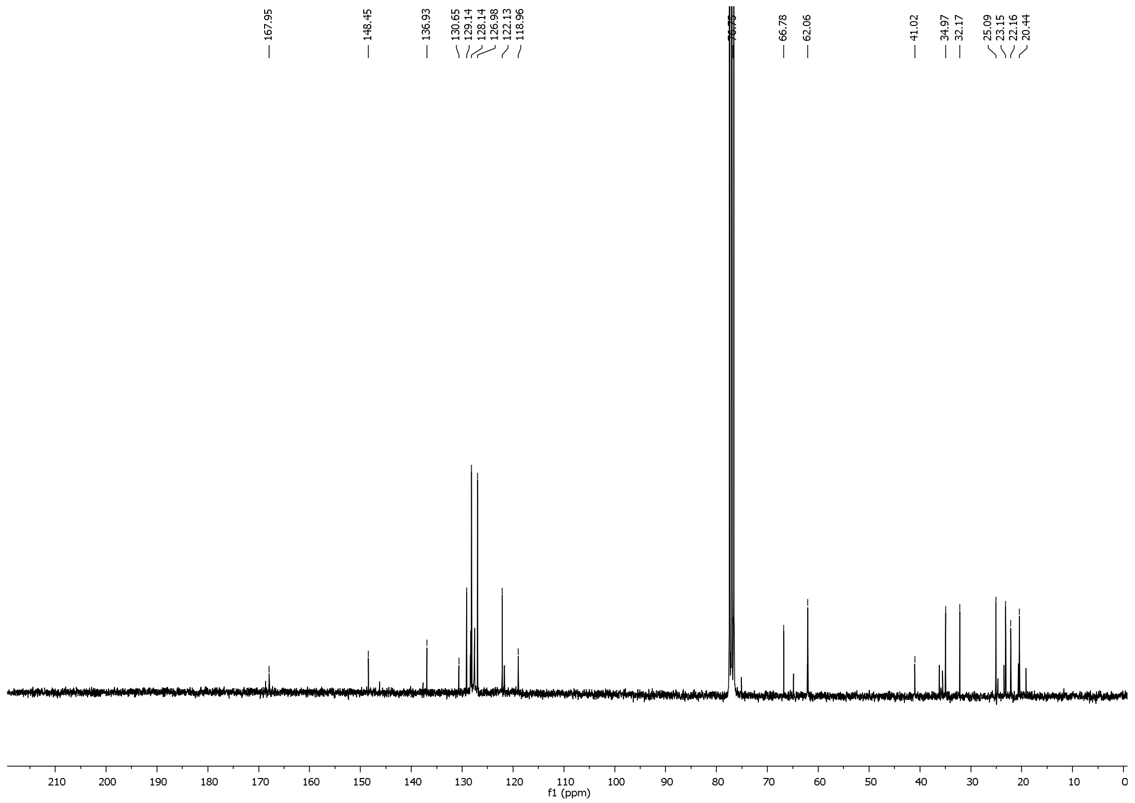

Figure SI-68. ^{13}C-NMR (75.5 MHz, $CDCl_3$) spectra of compound **3m**.

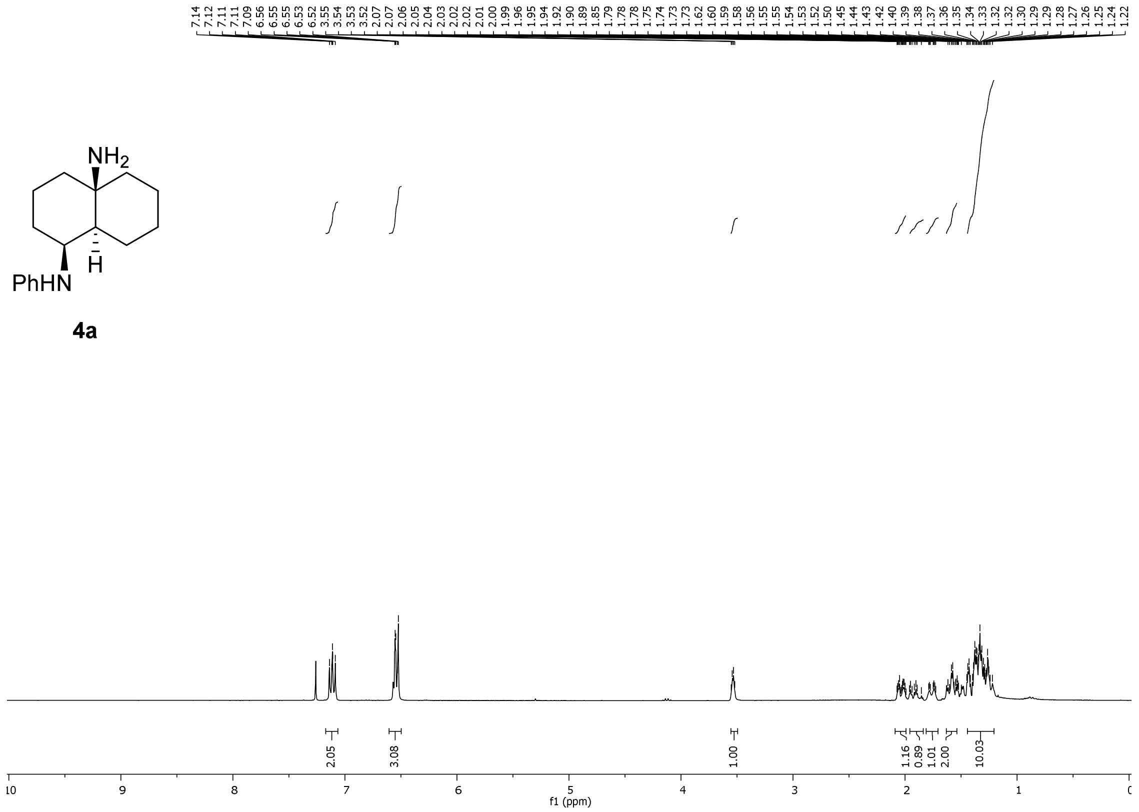

Figure SI-69. ^{1}H-NMR (300 MHz, $CDCl_3$) spectra of compound **4a**.

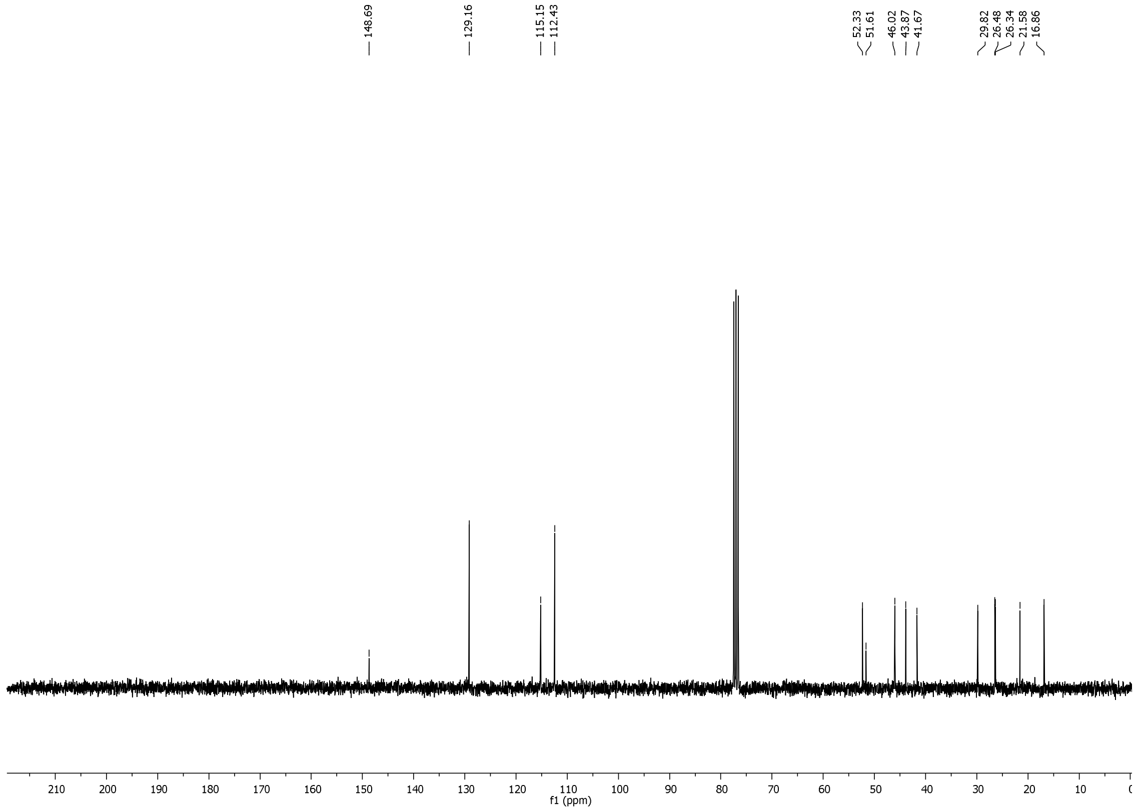

Figure SI-70. ^{13}C-NMR (75.5 MHz, $CDCl_3$) spectra of compound **4a**.

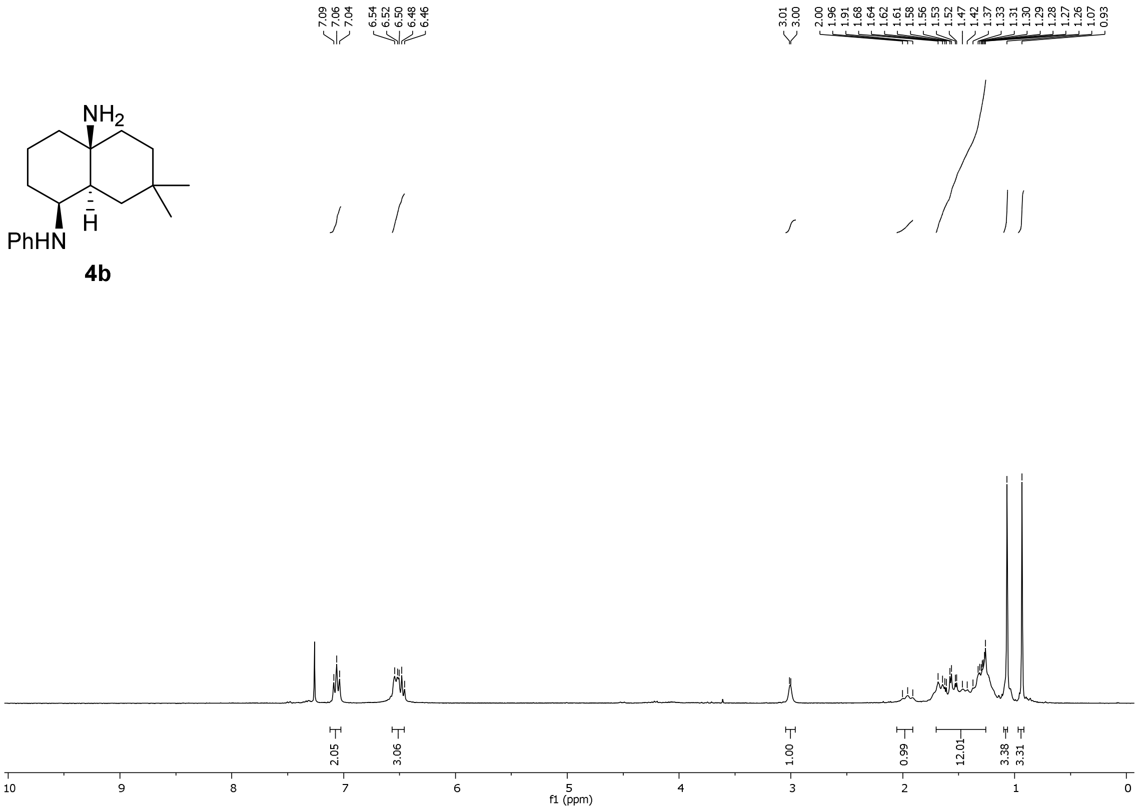

Figure SI-71. ^{1}H-NMR (300 MHz, $CDCl_3$) spectra of compound **4b**.

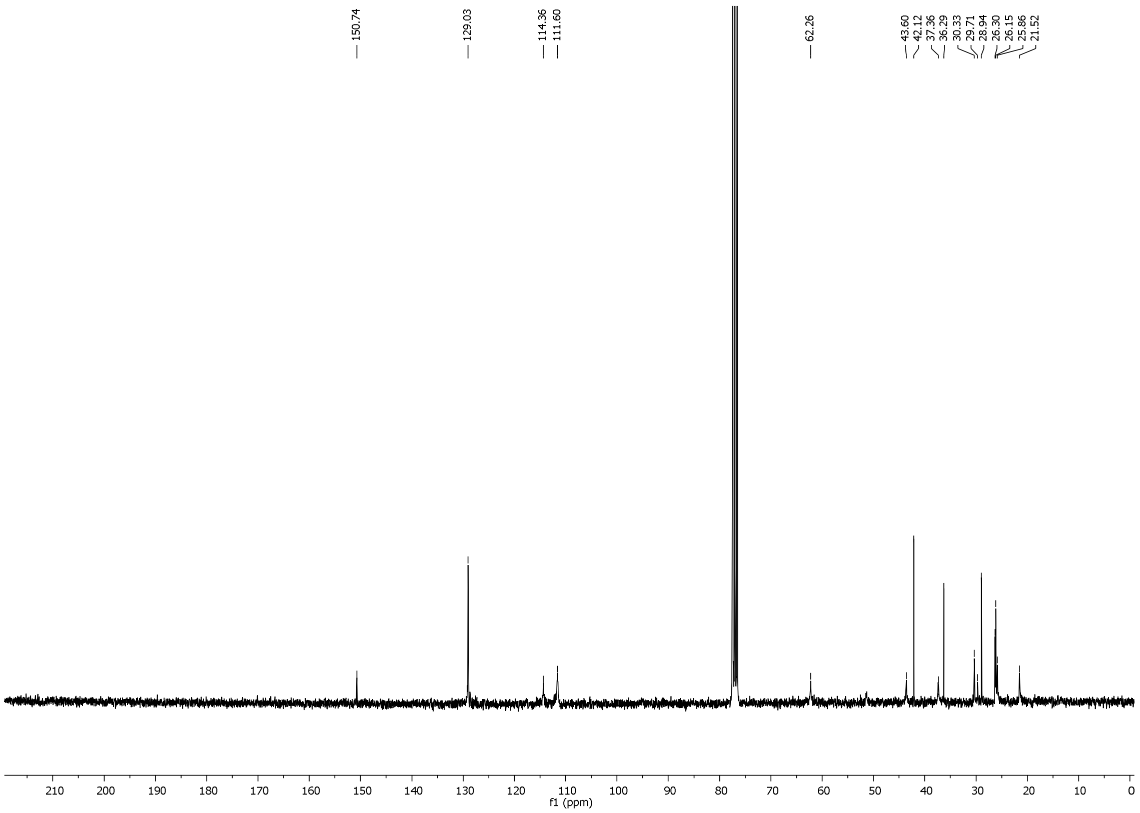

Figure SI-72. ^{13}C-NMR (75.5 MHz, $CDCl_3$) spectra of compound **4b**.

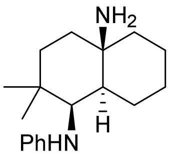

4c

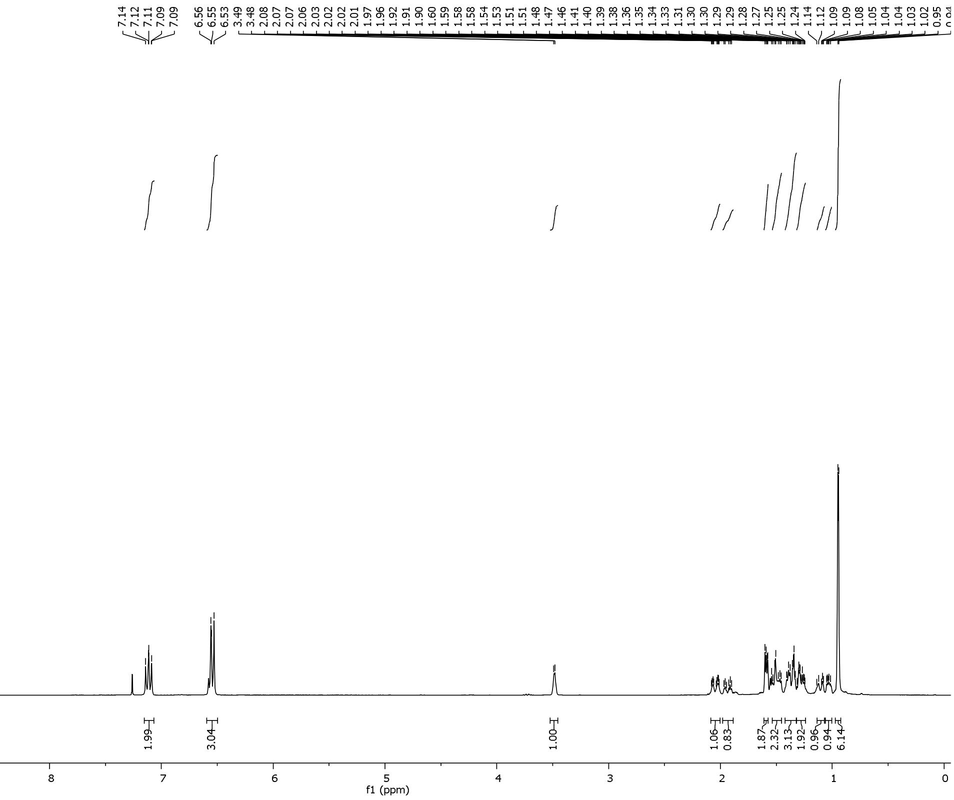

Figure SI-73. ^{1}H-NMR (300 MHz, $CDCl_3$) spectra of compound **4c**.

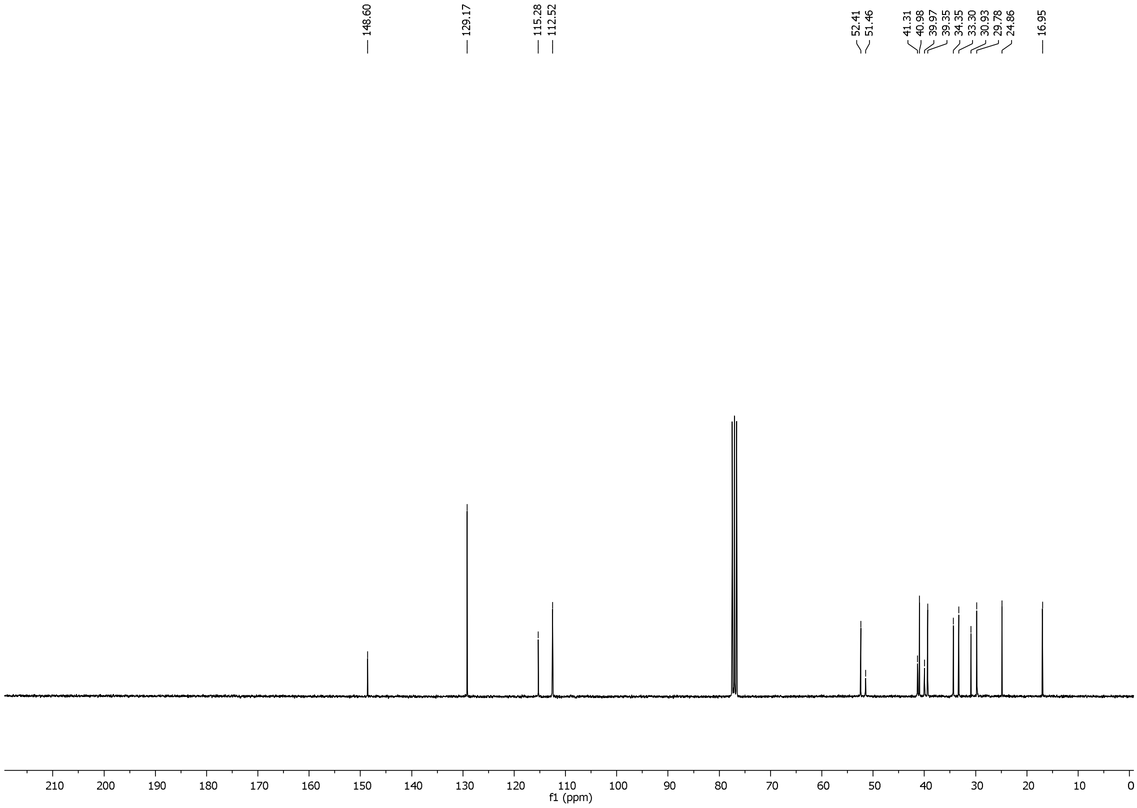

Figure SI-74. ^{13}C-NMR (75.5 MHz, $CDCl_3$) spectra of compound **4c**.

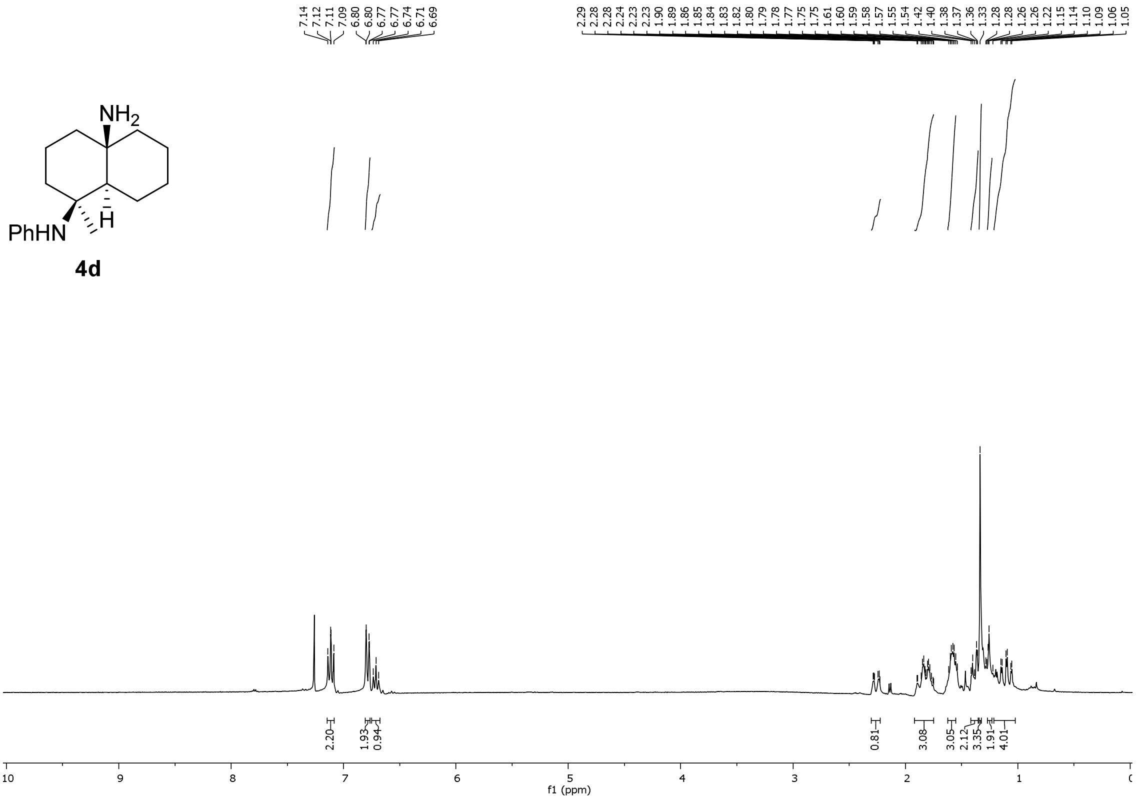

Figure SI-75. ^{1}H-NMR (300 MHz, CDCl$_3$) spectra of compound **4d**.

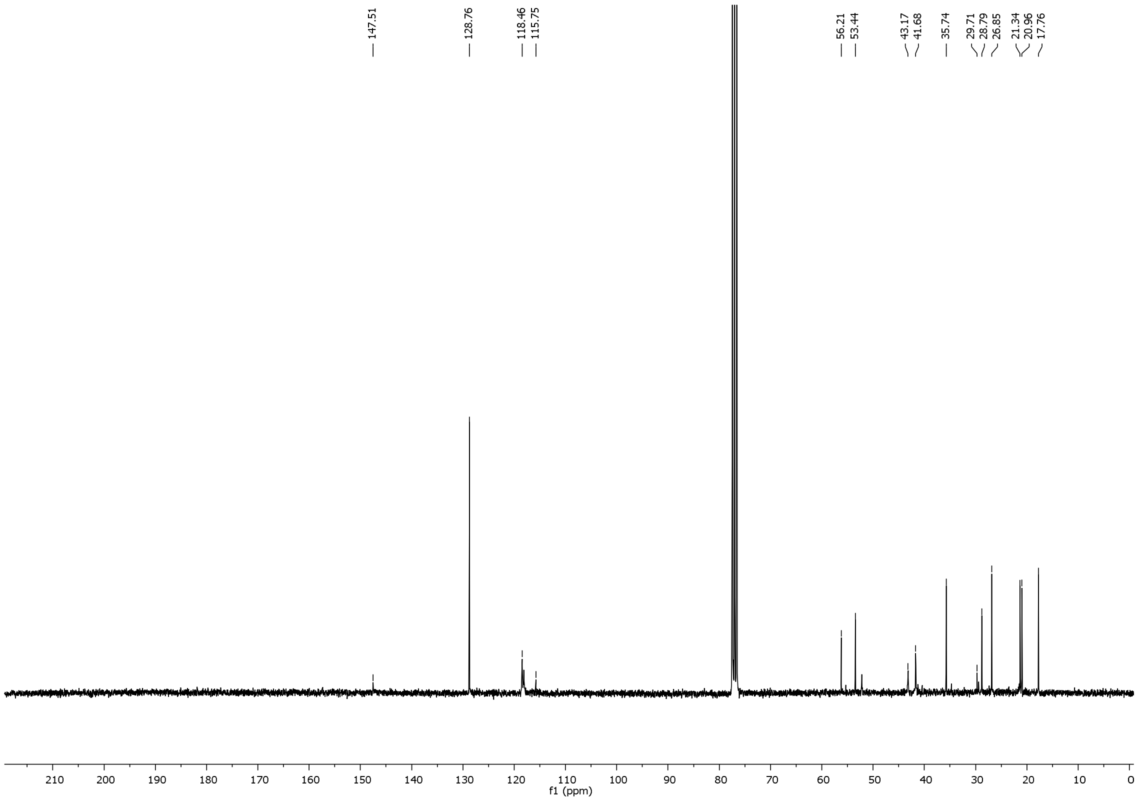

Figure SI-76. ^{13}C-NMR (75.5 MHz, CDCl$_3$) spectra of compound **4d**.

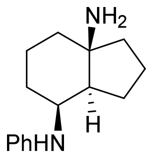

4e

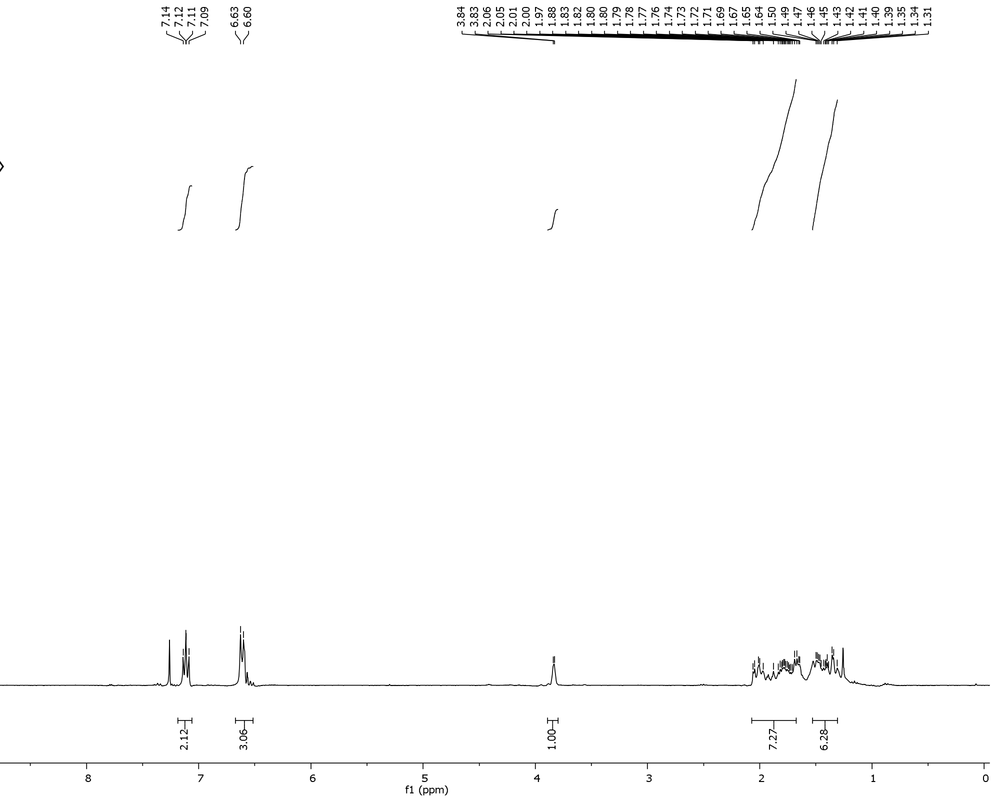

Figure SI-77. ^{1}H-NMR (300 MHz, $CDCl_3$) spectra of compound **4e**.

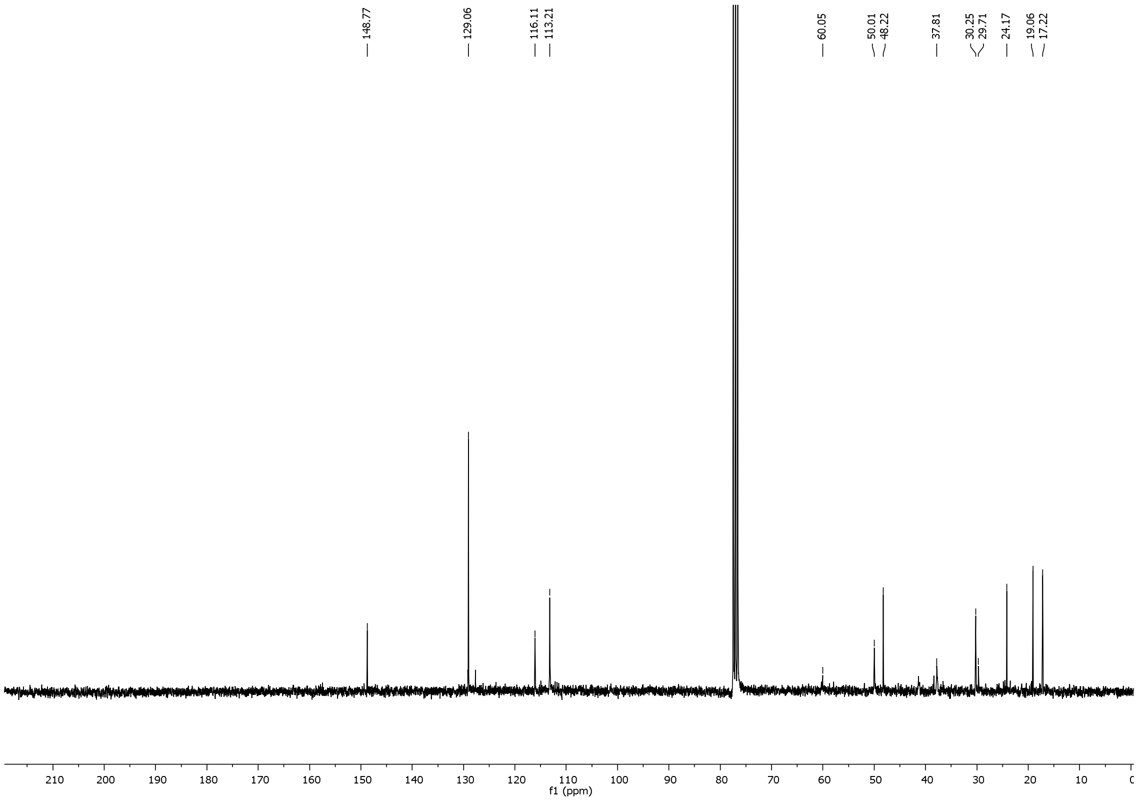

Figure SI-78. ^{13}C-NMR (75.5 MHz, $CDCl_3$) spectra of compound **4e**.

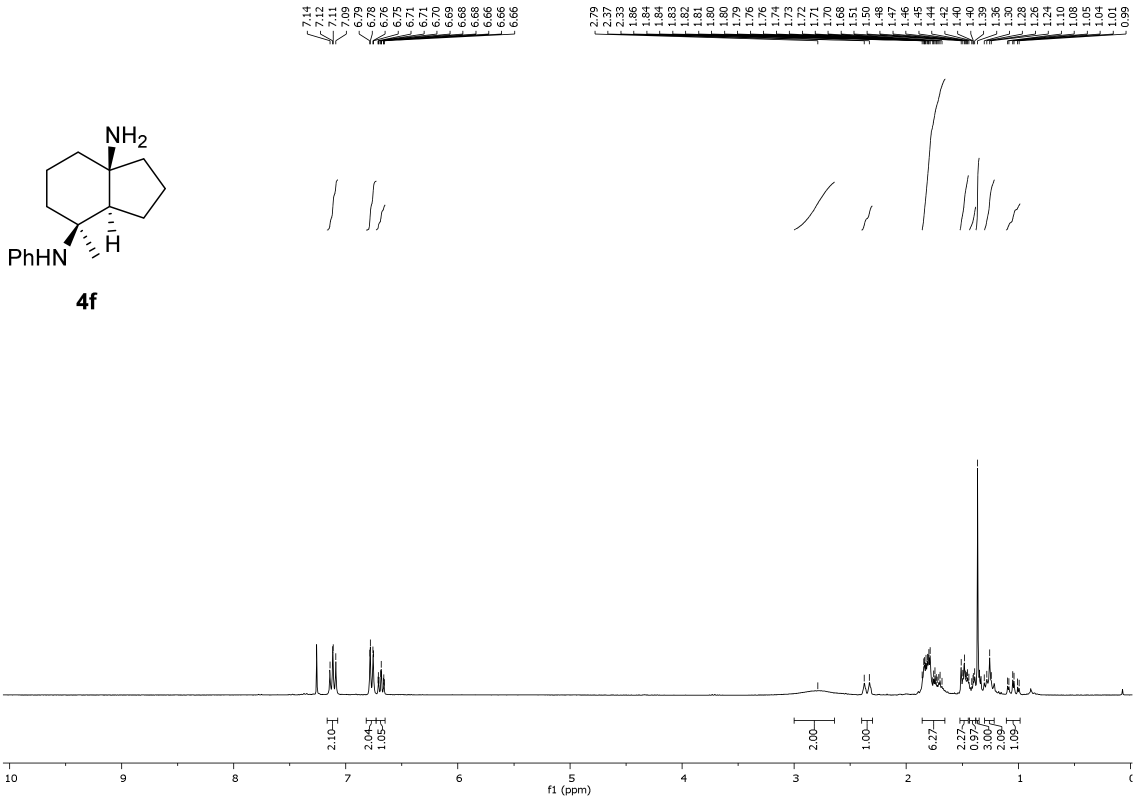

4f

Figure SI-79. ^{1}H-NMR (300 MHz, $CDCl_3$) spectra of compound **4f**.

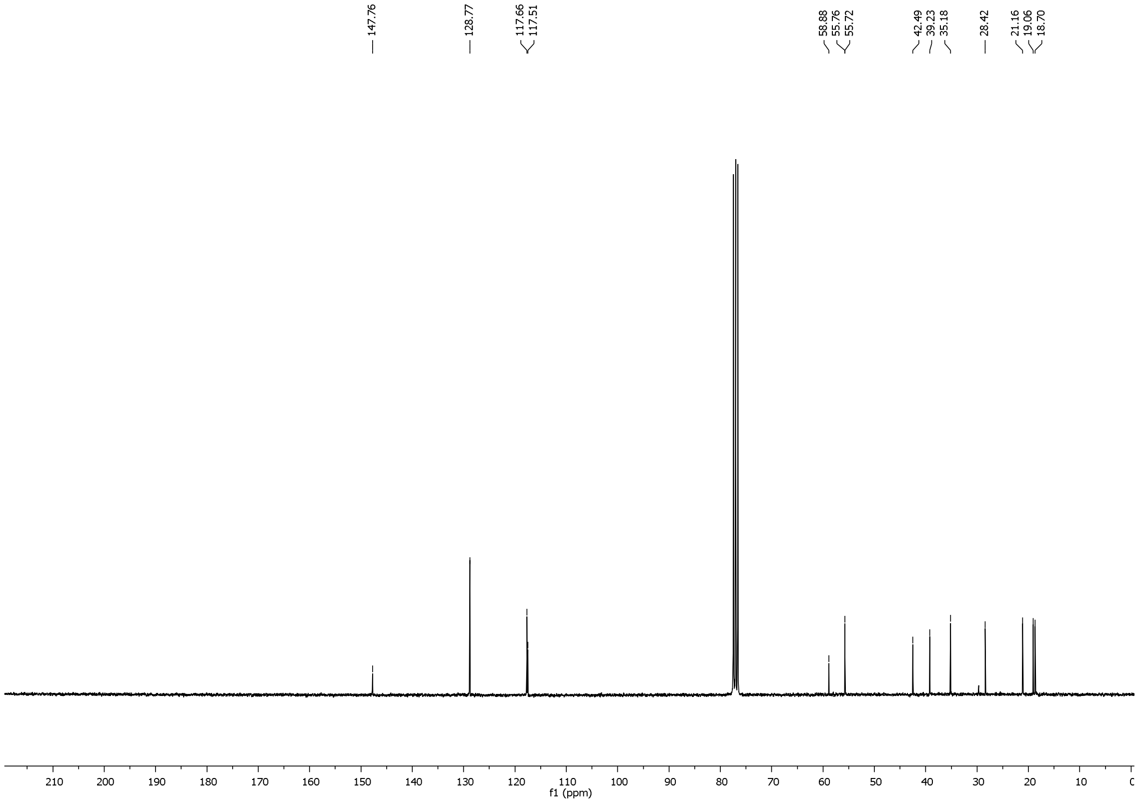

Figure SI-80. ^{13}C-NMR (75.5 MHz, $CDCl_3$) spectra of compound **4f**.

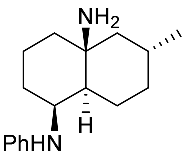

4g

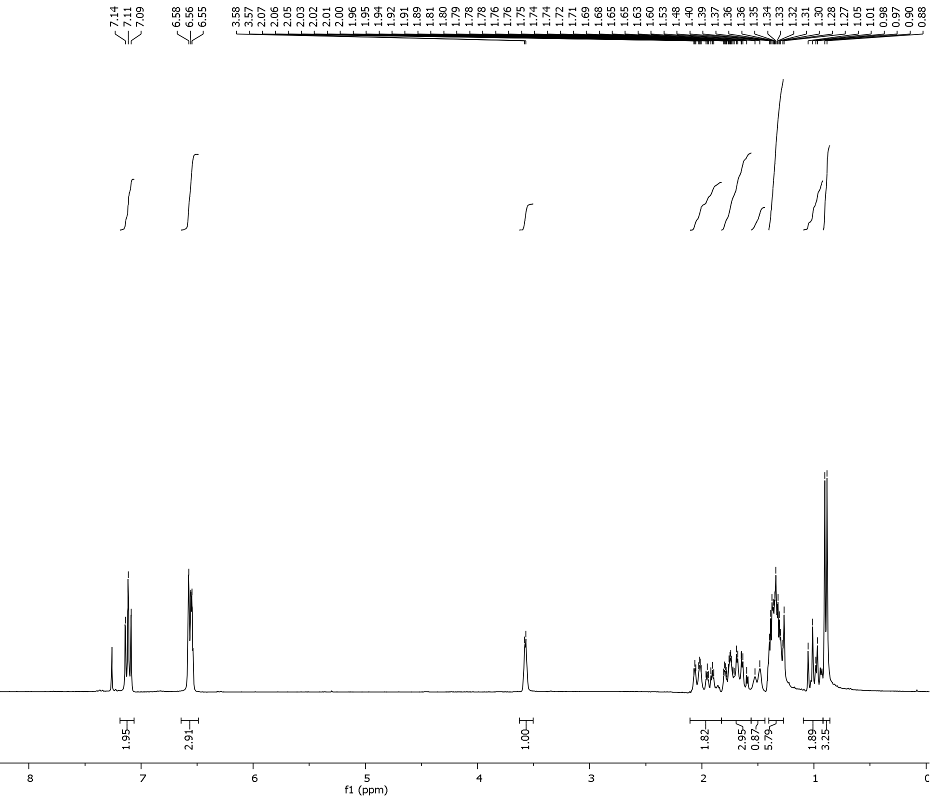

Figure SI-81. ^{1}H-NMR (300 MHz, $CDCl_3$) spectra of compound **4g**.

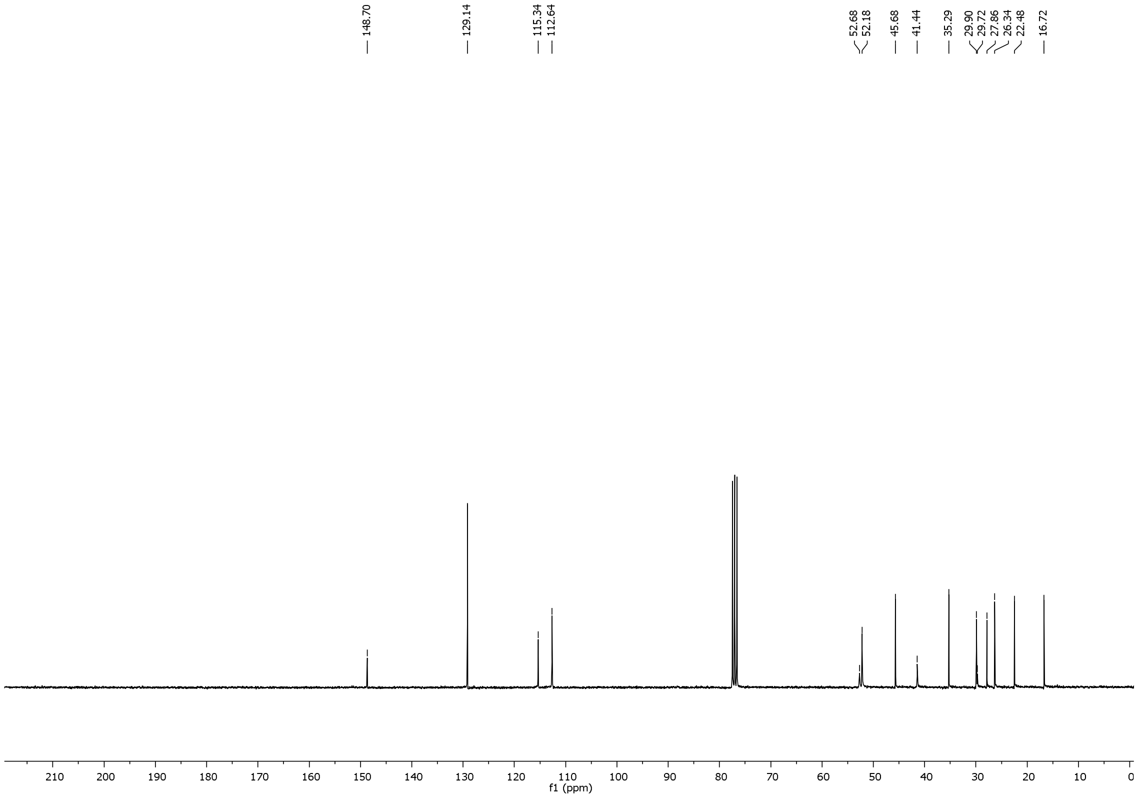

Figure SI-82. ^{13}C-NMR (75.5 MHz, $CDCl_3$) spectra of compound **4g**.

4. HPLC traces

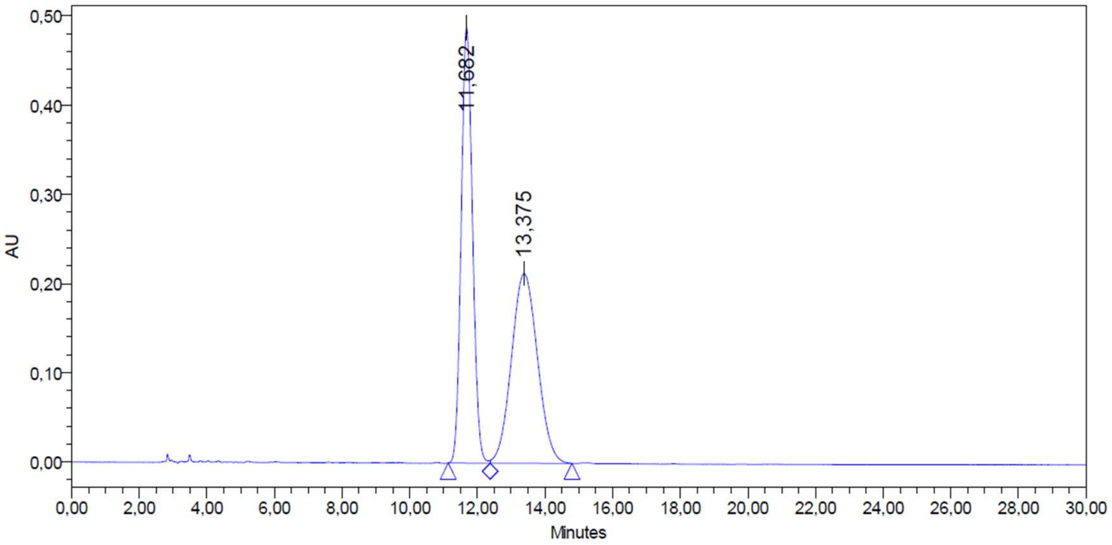

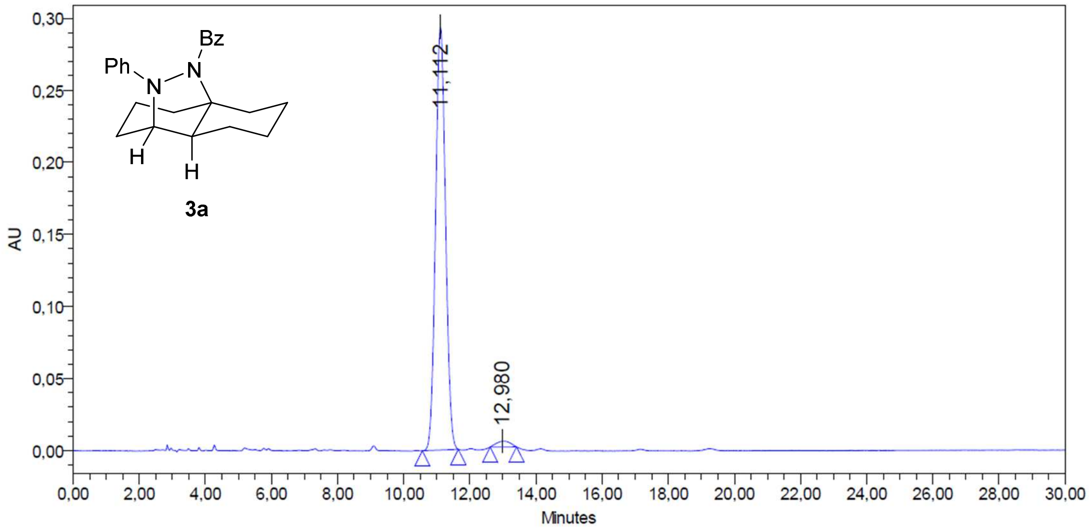

Peak Results

	RT	Area	Height	% Area
1	11,112	6118788	293941	98,12
2	12,980	117240	3961	1,88

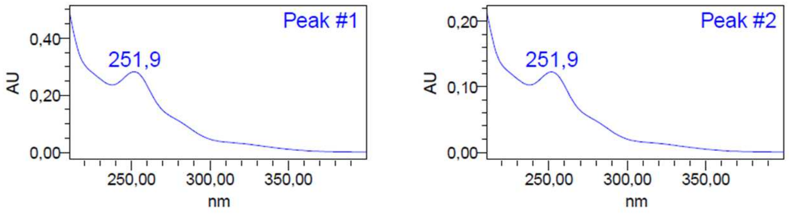

Figure SI-83. HPLC traces for racemic and compound **3a**.

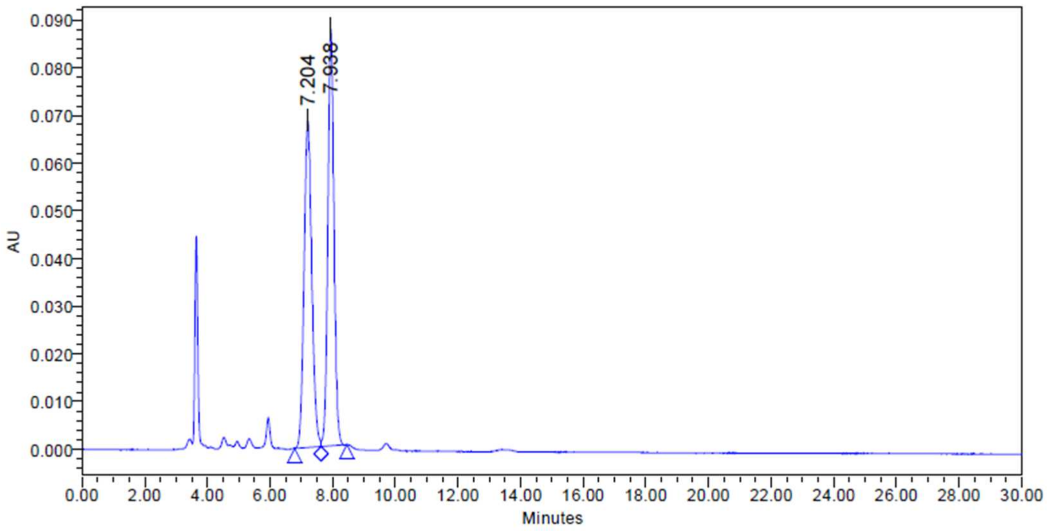

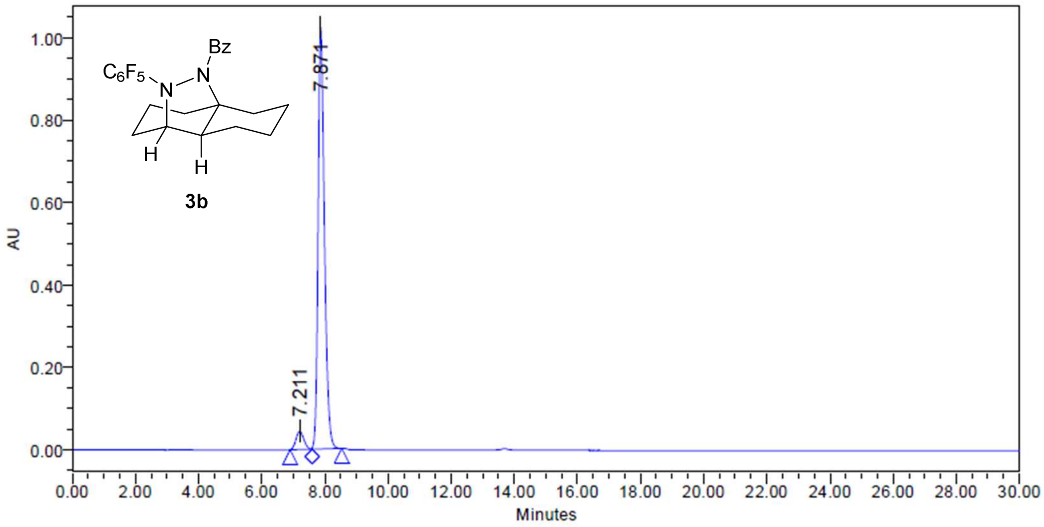

Peak Results

	RT	Area	Height	% Area
1	7.211	748560	43660	5.05
2	7.871	14067878	1023347	94.95

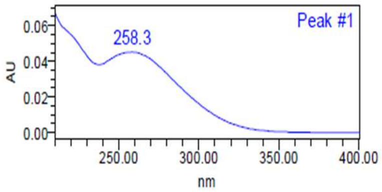

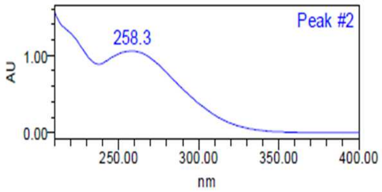

Figure SI-84. HPLC traces for racemic and compound **3b**.

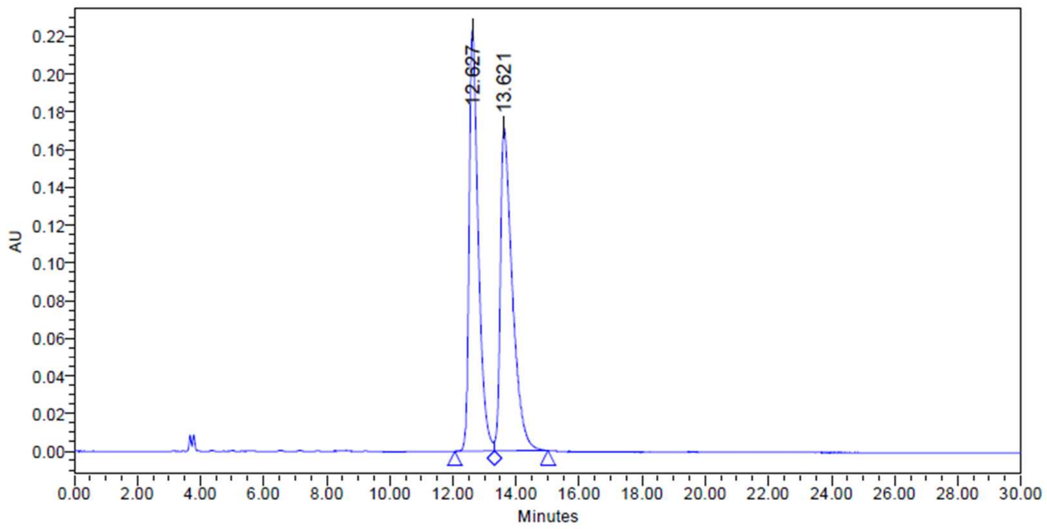

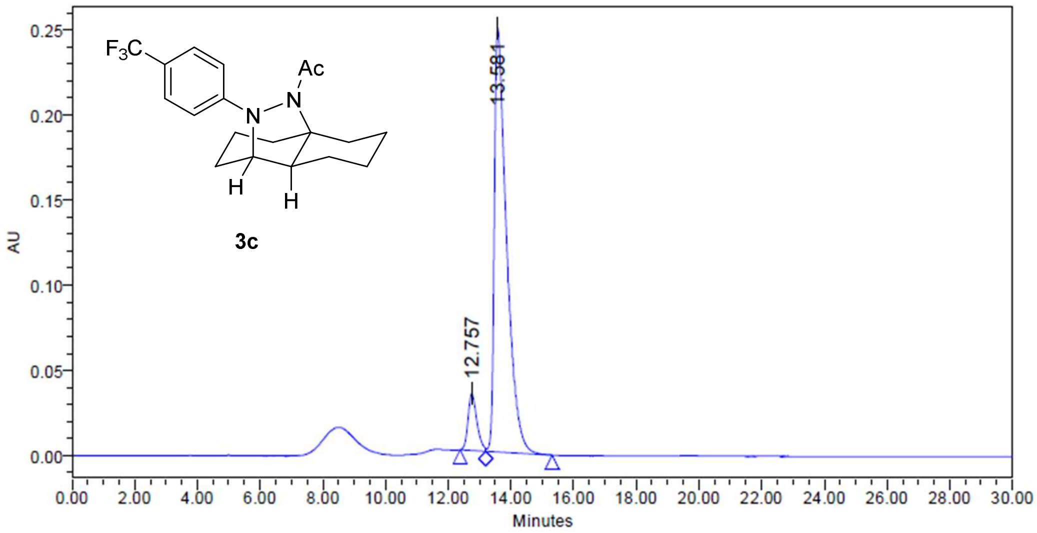

Peak Results

	RT	Area	Height	% Area
1	12.757	649885	33297	8.83
2	13.581	6708819	248571	91.17

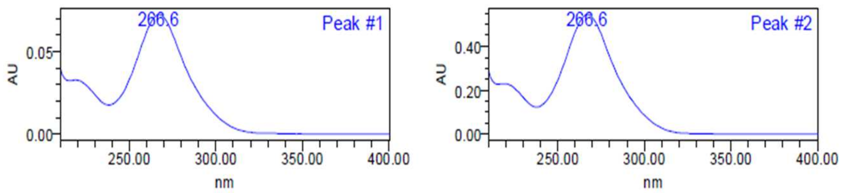

Figure SI-85. HPLC traces for racemic and compound **3c**.

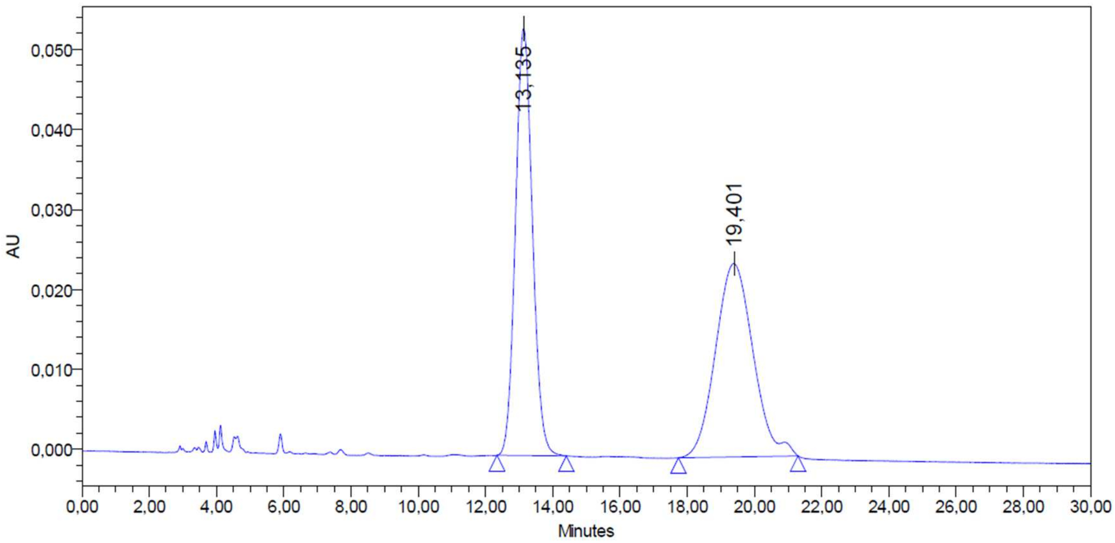

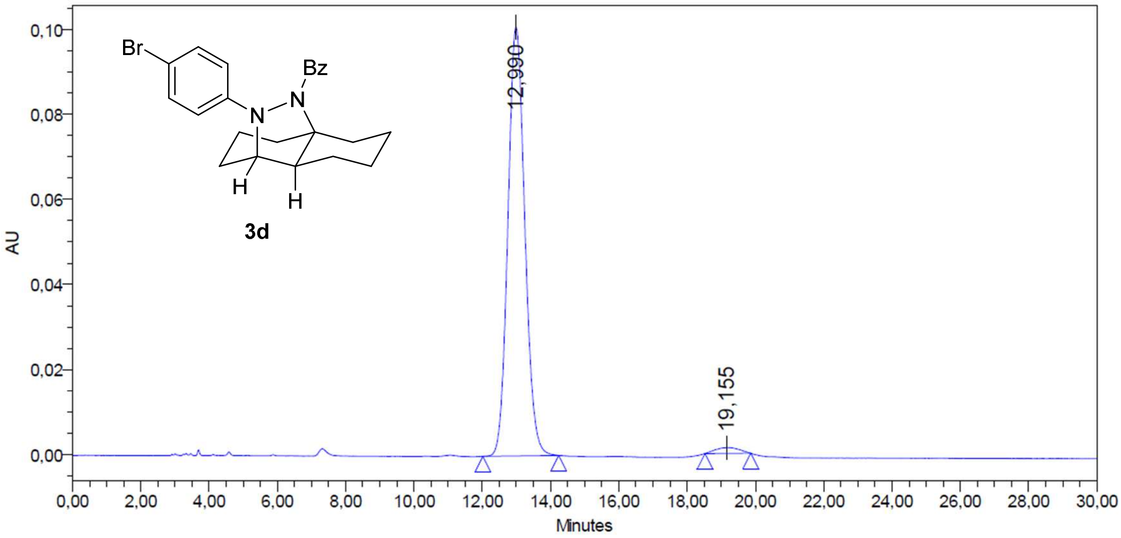

Peak Results

	RT	Area	Height	% Area
1	12,990	3463474	100964	98,14
2	19,155	65812	1374	1,86

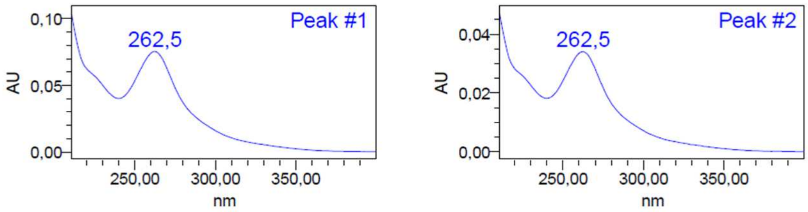

Figure SI-86. HPLC traces for racemic and compound **3d**.

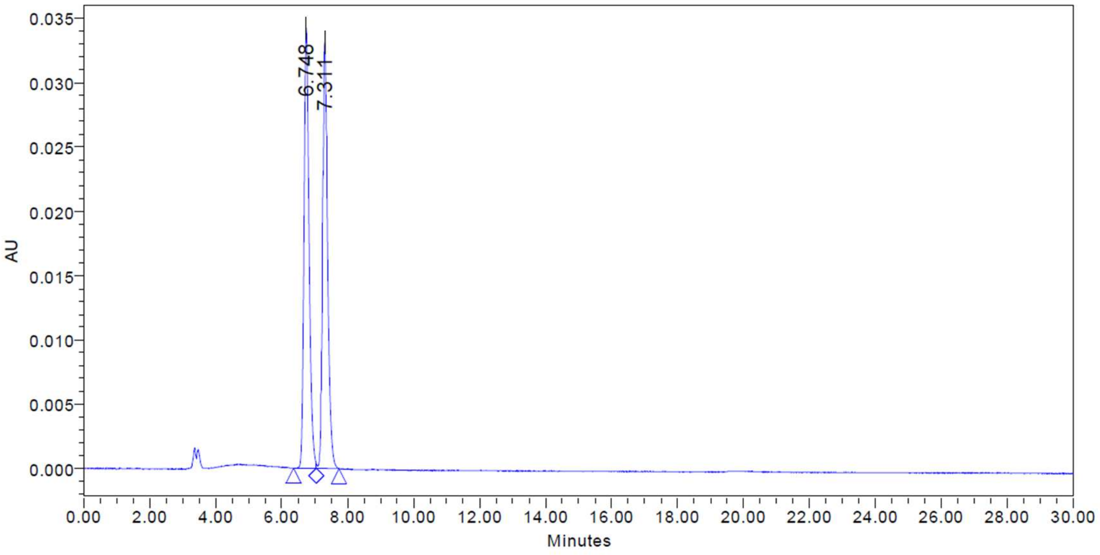

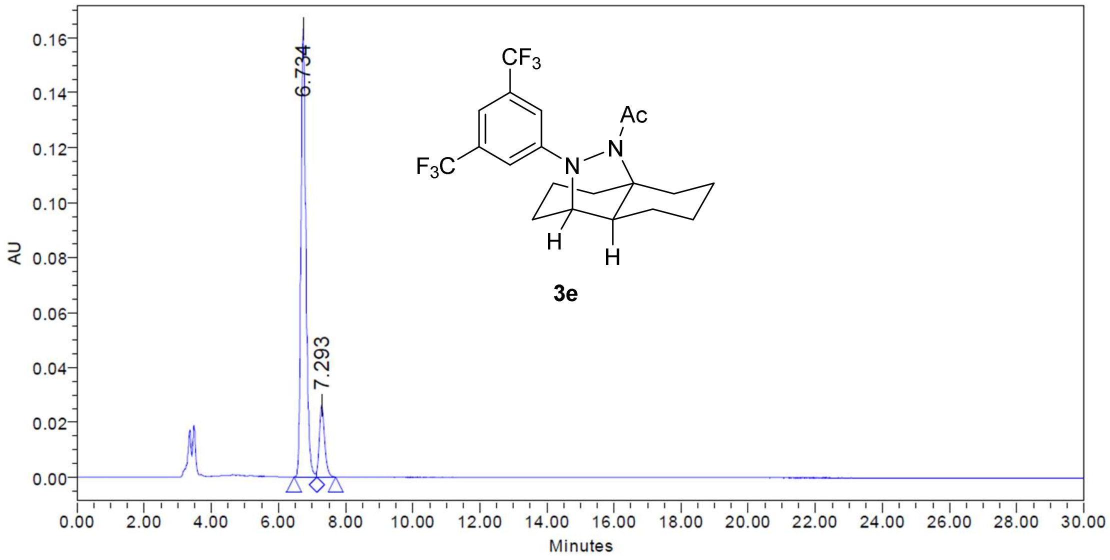

Peak Results

	RT	Area	Height	% Area
1	6.734	1722655	163423	85.95
2	7.293	281670	26139	14.05

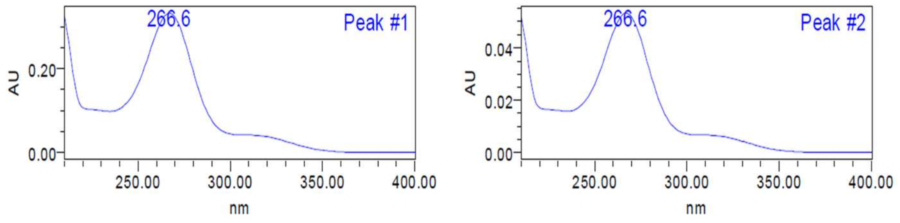

Figure SI-87. HPLC traces for racemic and compound **3e**.

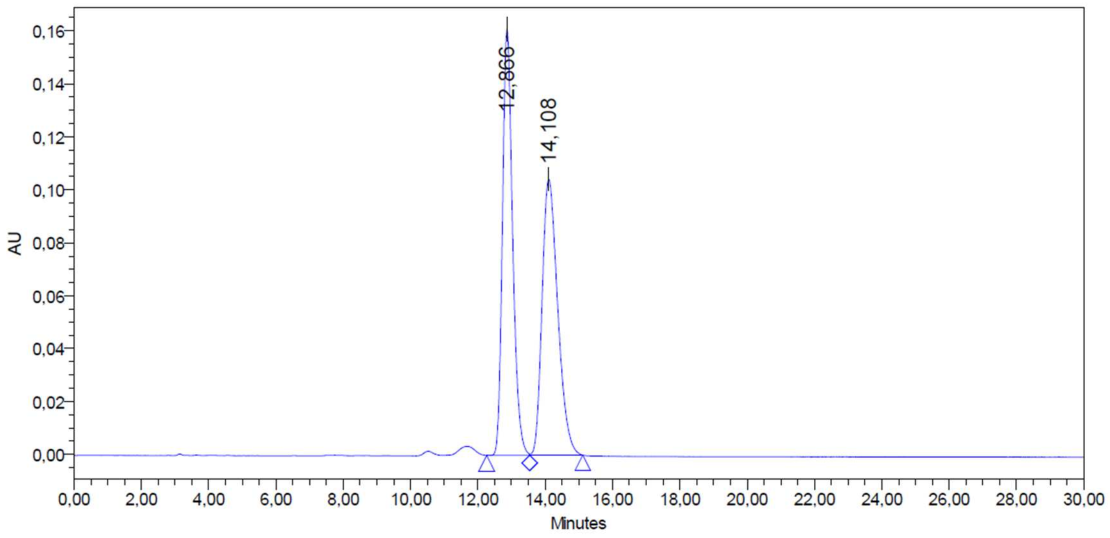

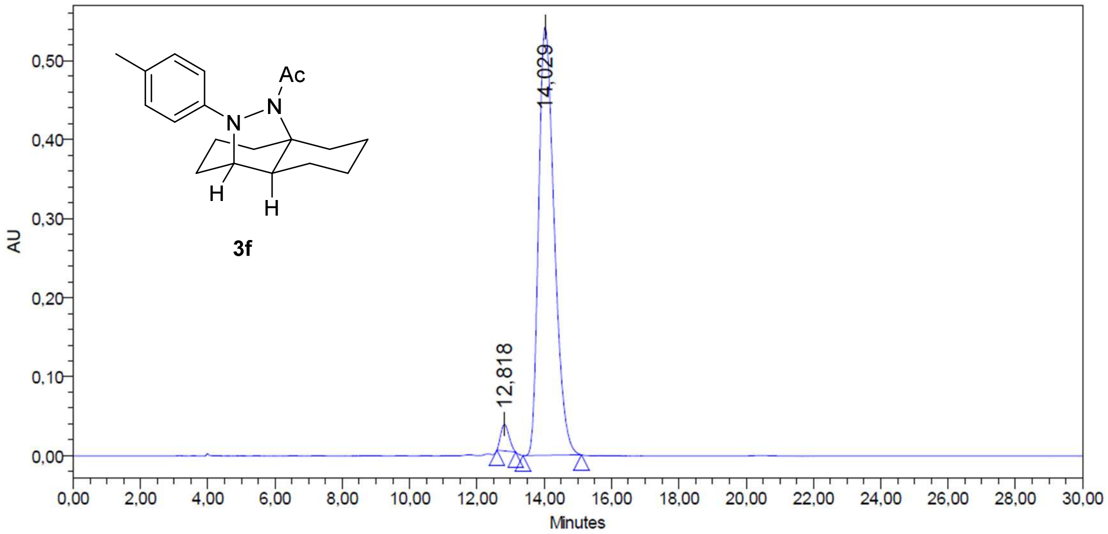

Peak Results

	RT	Area	Height	% Area
1	12,818	570287	33353	3,15
2	14,029	17519011	542380	96,85

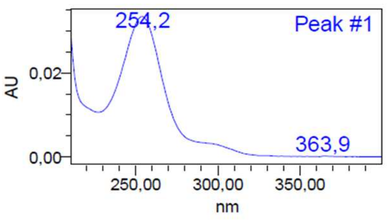

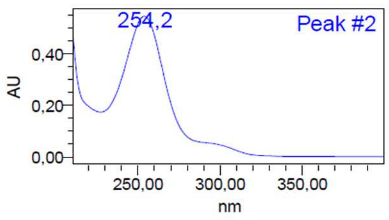

Figure SI-88. HPLC traces for racemic and compound **3f**.

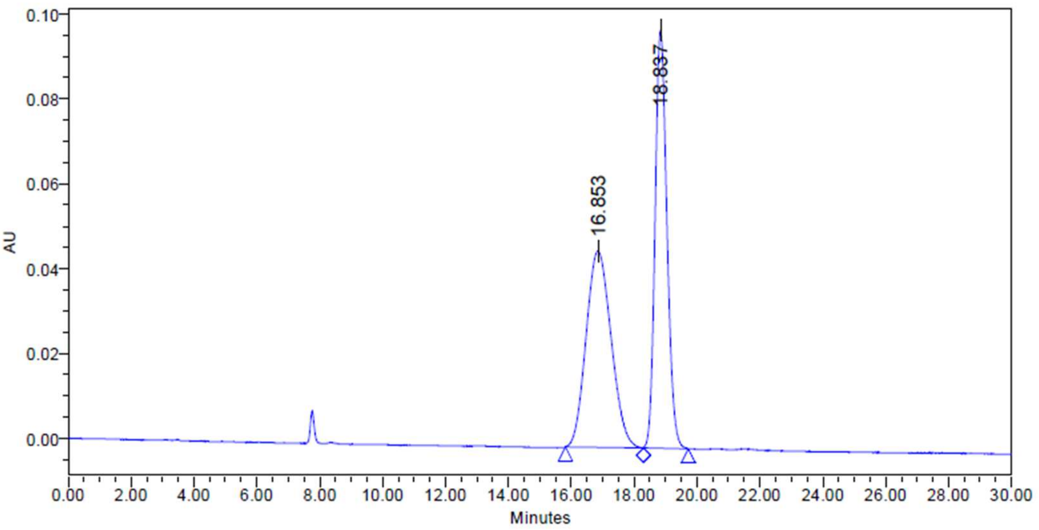

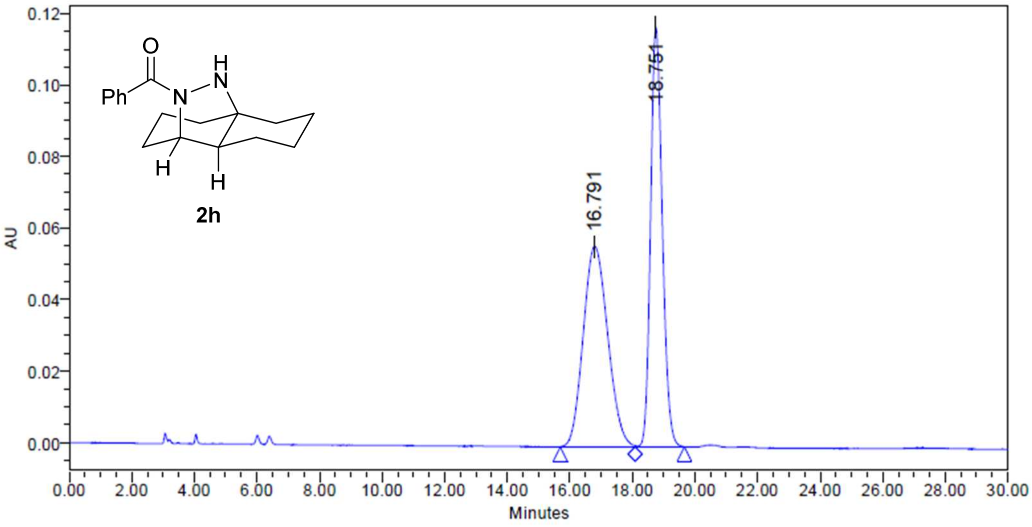

Peak Results

	RT	Area	Height	% Area
1	16.791	3022452	56020	49.98
2	18.751	3024633	117359	50.02

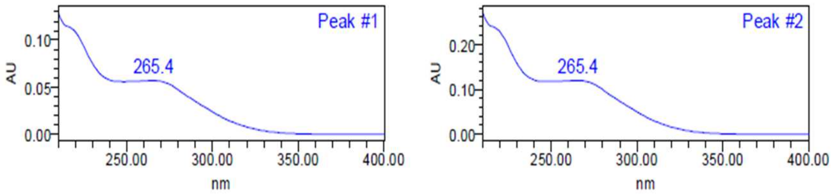

Figure SI-89. HPLC traces for racemic and compound **2h**.

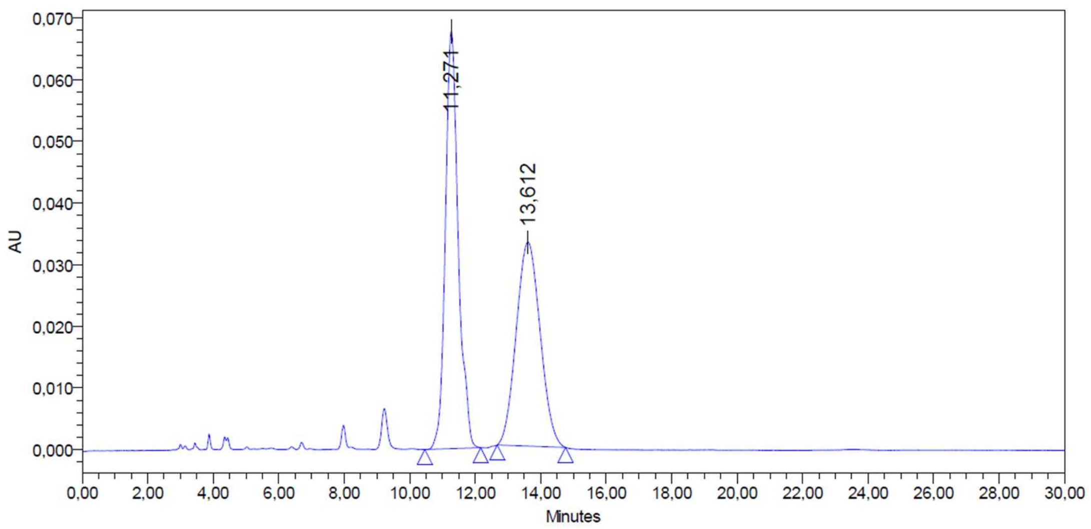

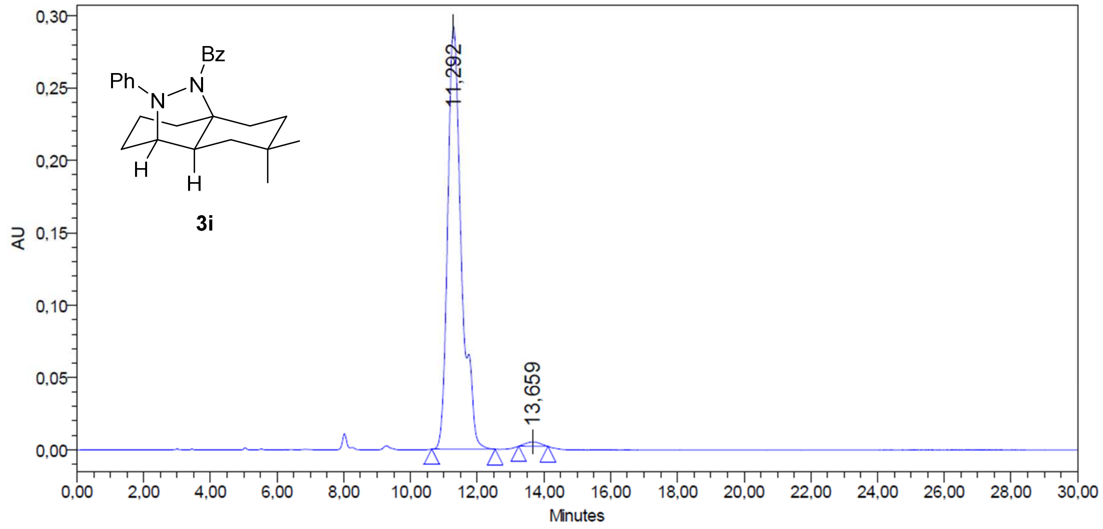

Peak Results

	RT	Area	Height	% Area
1	11,292	8320436	292327	98,87
2	13,659	95486	3086	1,13

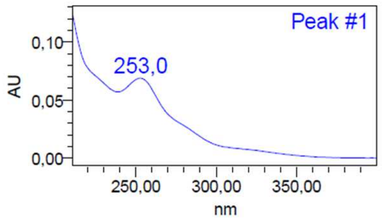

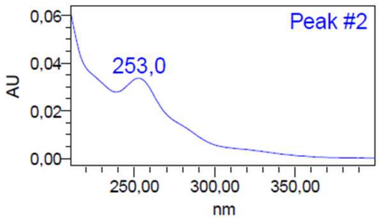

Figure SI-90. HPLC traces for racemic and compound **3i**.

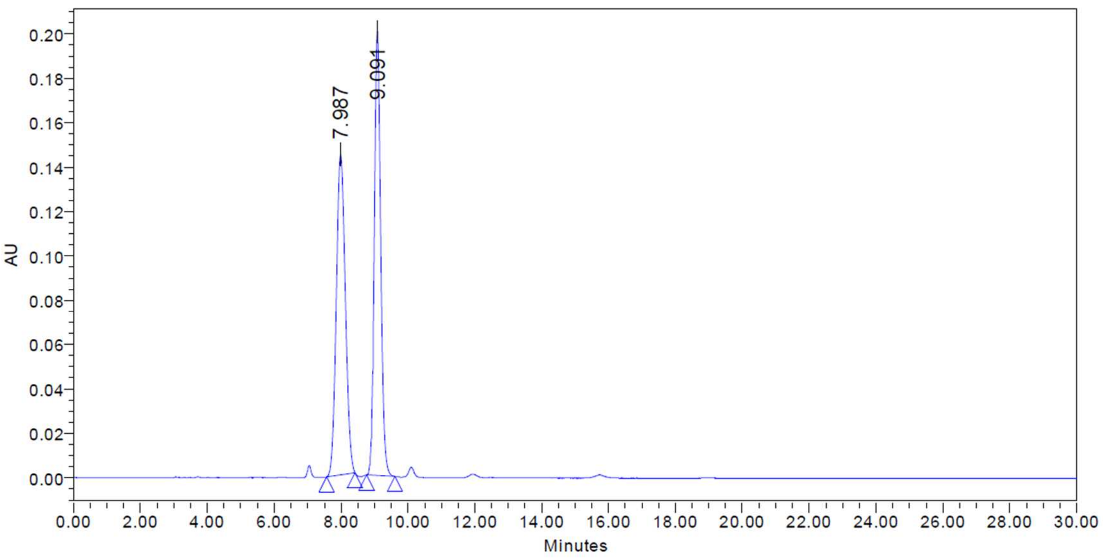

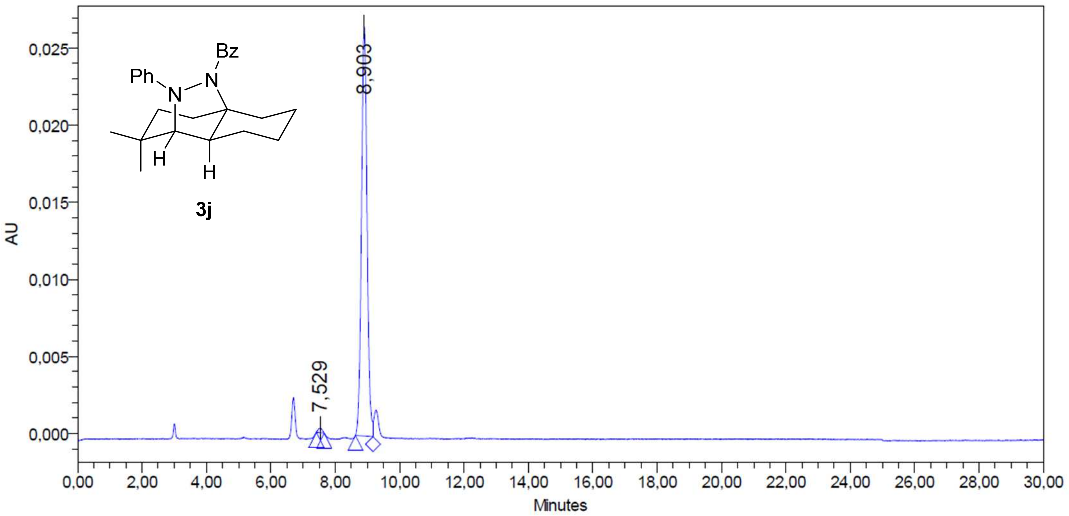

Peak Results

	RT	Area	Height	% Area
1	7,529	2576	317	0,80
2	8,903	319020	26579	99,20

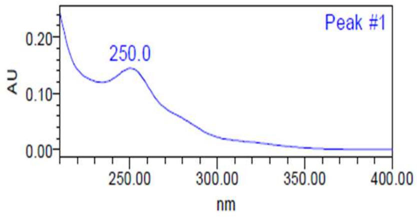

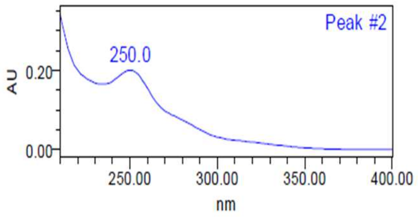

Figure SI-91. HPLC traces for racemic and compound **3j**.

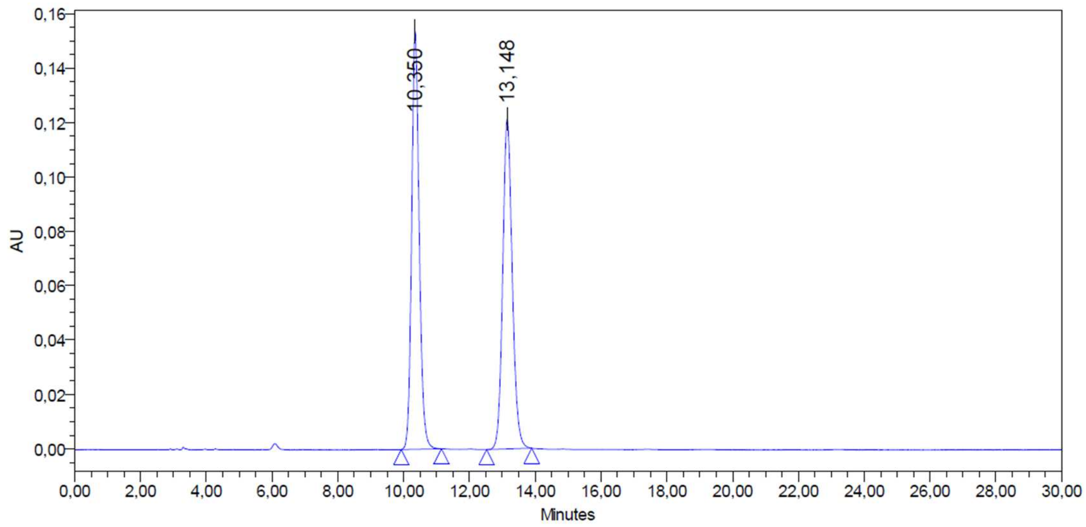

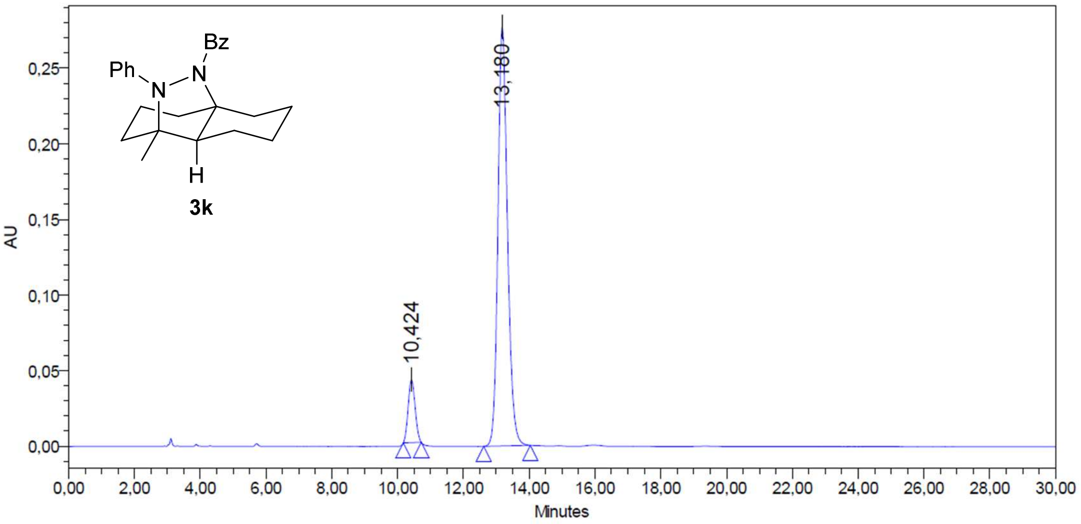

Peak Results

	RT	Area	Height	% Area
1	10,424	610017	41676	9,86
2	13,180	5577237	277322	90,14

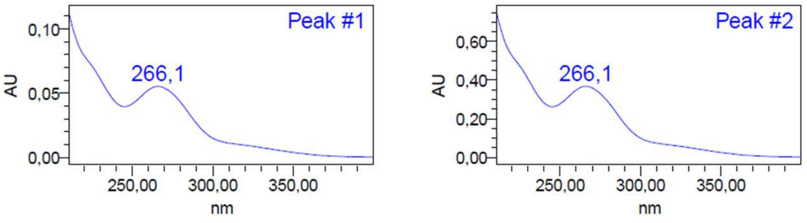

Figure SI-92. HPLC traces for racemic and compound **3k**.

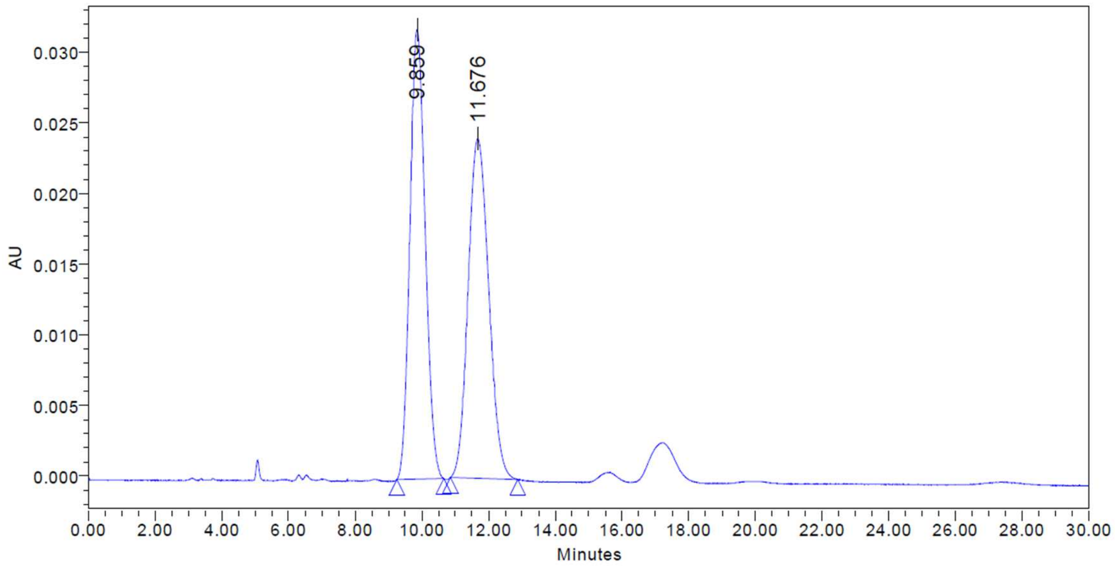

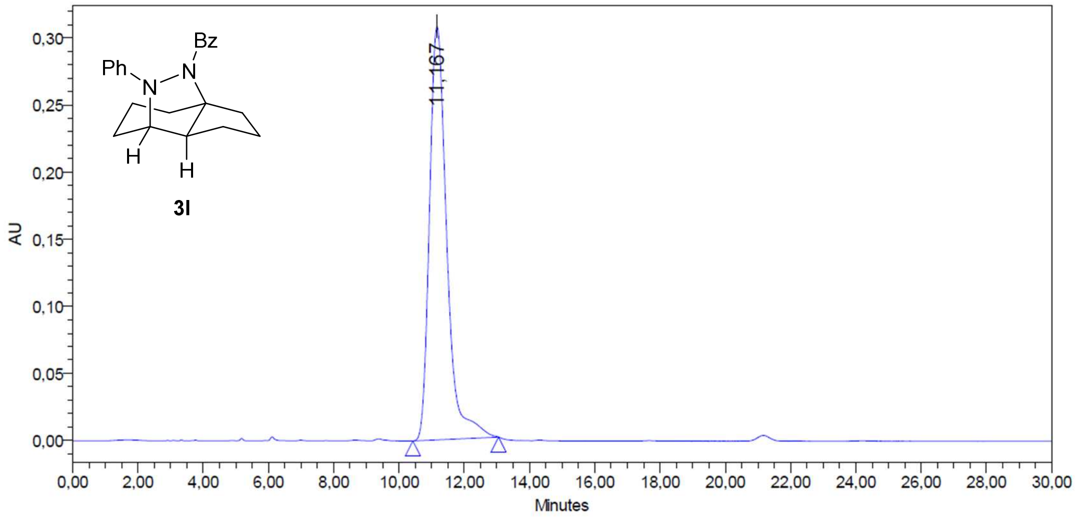

Peak Results

	RT	Area	Height	% Area
1	11,167	11318645	308517	100,00

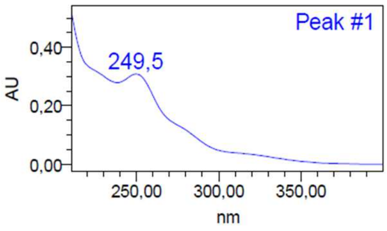

Figure SI-93. HPLC traces for racemic and compound **3l**.

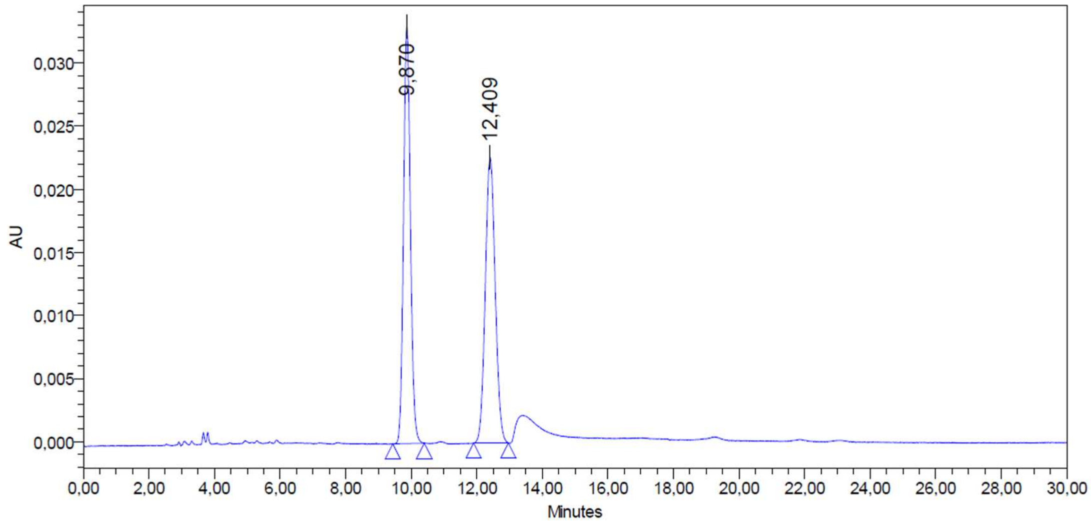

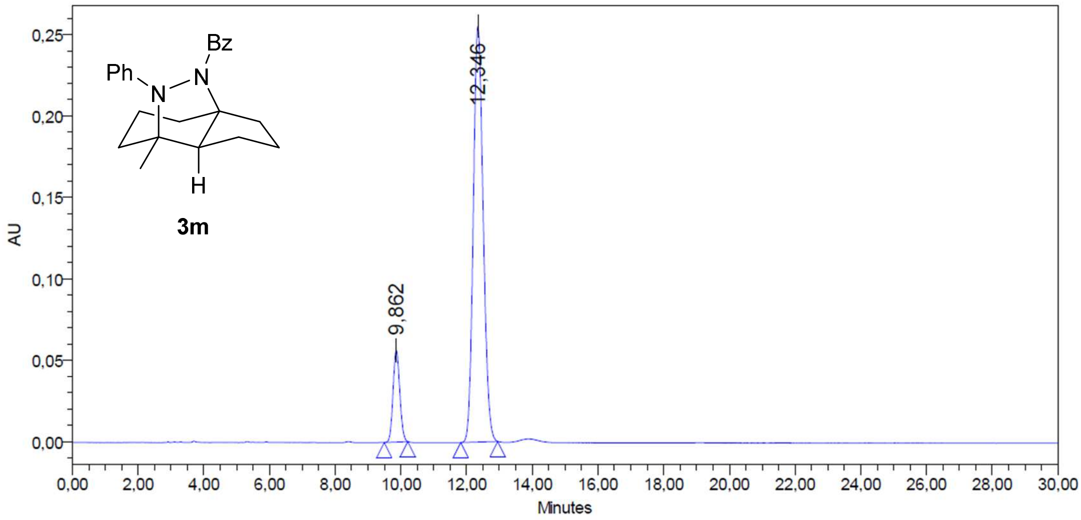

Peak Results

	RT	Area	Height	% Area
1	9,862	797434	56429	13,03
2	12,346	5321302	255278	86,97

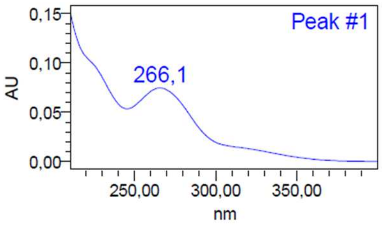

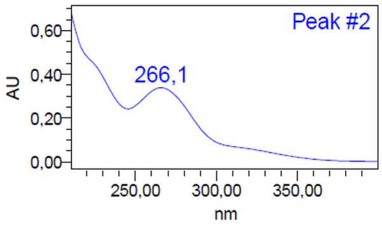

Figure SI-94. HPLC traces for racemic and compound **3m**.

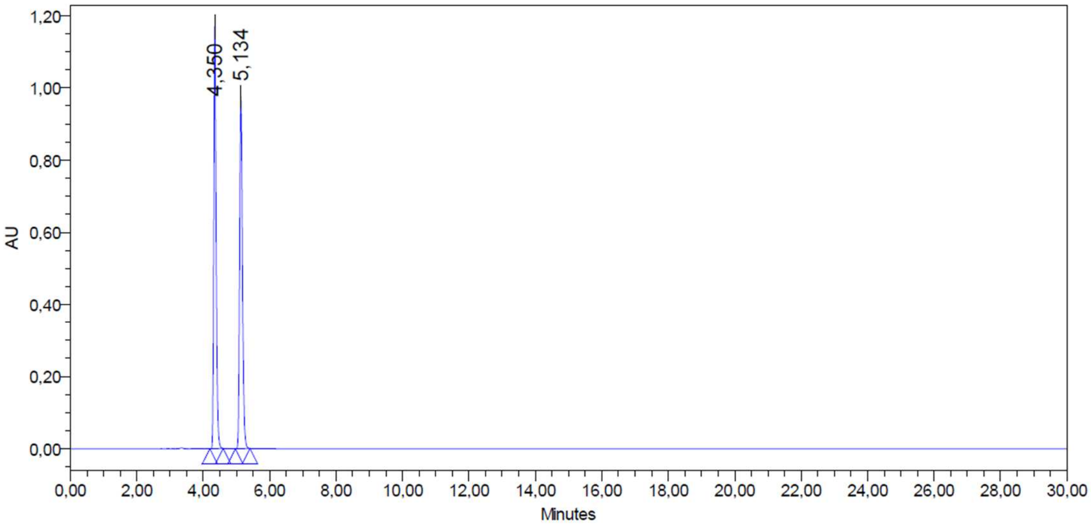

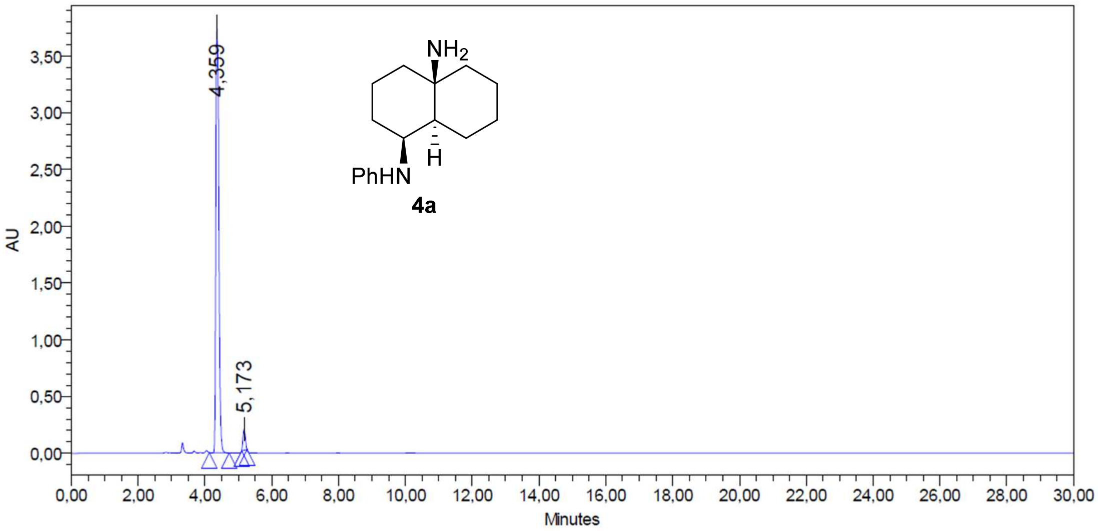

Peak Results

	RT	Area	Height	% Area
1	4,359	23521853	3748377	96,24
2	5,173	918361	185240	3,76

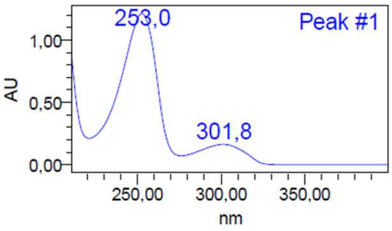

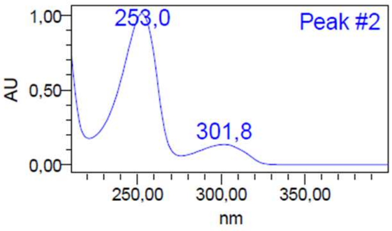

Figure SI-95. HPLC traces for racemic and compound **4a**.

5. X-ray details

The enantiopure sample of compound **(1*S*,4a*S*,8a*R*)-3j** was obtained following *General Procedure I* and *II* using catalyst **(*R*)-3f** (13 mg, 0.015 mmol, 0.10 equiv). Spectroscopic data matched with the one obtained for the other enantiomer of compound **(1*R*,4a*R*,8a*S*)-3j** except the optical rotation: $[\alpha]_D^{20}$: -9.2 (*c* 1.00, CH_2Cl_2). Crystals of the sample were obtained after slow evaporation of a mixture of CH_2Cl_2/Hexane in a screw-top vial.

Summary of Data CCDC 2091628

Crystal Data for **(1*S*,4a*S*,8a*R*)-3j** $C_{25}H_{30}N_2O$ (M =374.51 g/mol): orthorhombic, space group $P2_12_12_1$ (no. 19), a = 10.09480(5) Å, b = 12.79812(6) Å, c 15.78055(7) Å, V = 2038.760(17) $Å^3$, Z = 4, T = 150.01(10) K, μ(CuKα) = 0.572 mm^{-1}, *Dcalc* 1.220 g/cm^3, 38523 reflections measured (8.896° ≤ 2Θ ≤ 145.932°), 4064 unique (R_{int} = 0.0309, R_{sigma} = 0.0134) which were used in all calculations. The final R_1 was 0.0285 (I > 2σ(I)) and wR_2 was 0.0744 (all data).

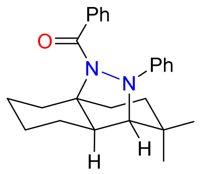

(1*S*,4a*S*,8a*R*)-3j

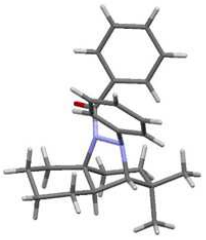

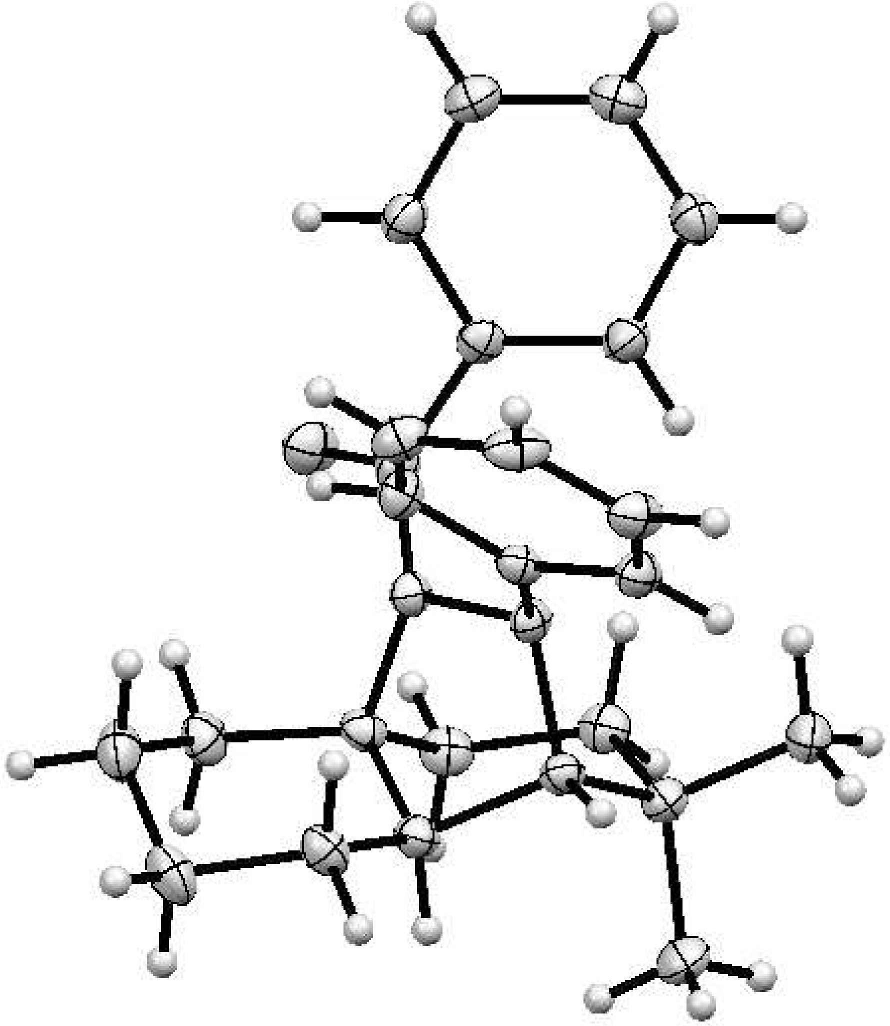

Figure SI-96. X-Ray structure and ORTEP diagram (50% probability) for **(1*S*,4a*S*,8a*R*)-3j**.

Table SI-2. Crystal data and structure refinement for **(1*S*,4a*S*,8a*R*)-3j.**

Identification code	a20200290_JS250Brem
Empirical formula	$C_{25}H_{30}N_2O$
Formula weight	374.51
Temperature/K	150.01(10)
Crystal system	orthorhombic
Space group	$P2_12_12_1$
a/Å	10.09480(5)
b/Å	12.79812(6)
c/Å	15.78055(7)
α/°	90
β/°	90
γ/°	90
Volume/Å^3	2038.760(17)
Z	4
ρ_{calc}g/cm^3	1.220
μ/mm^{-1}	0.572
F(000)	808.0
Crystal size/mm^3	0.542 × 0.260 × 0.240
Radiation	CuKα (λ = 1.54184)
2Θ range for data collection/°	8.896 to 145.932
Index ranges	-12 ≤ h ≤ 12, -15 ≤ k ≤ 15, -19 ≤ l ≤ 19
Reflections collected	38523
Independent reflections	4064 [R_{int} = 0.0309, R_{sigma} = 0.0134]
Data/restraints/parameters	4064/0/255
Goodness-of-fit on F^2	1.085
Final R indexes [I>=2σ (I)]	R_1 = 0.0285, wR_2 = 0.0741
Final R indexes [all data]	R_1 = 0.0287, wR_2 = 0.0744
Largest diff. peak/hole / e Å^{-3}	0.17/-0.17
Flack parameter	0.00(6)
